# inSPIRE: An Open-Source Tool for Increased Mass Spectrometry Identification Rates Using Prosit Spectral Prediction

**DOI:** 10.1016/j.mcpro.2022.100432

**Published:** 2022-10-21

**Authors:** John A. Cormican, Yehor Horokhovskyi, Wai Tuck Soh, Michele Mishto, Juliane Liepe

**Affiliations:** 1 Max-Planck-Institute for Multidisciplinary Sciences (MPI-NAT), Göttingen, Germany; 2 Centre for Inflammation Biology and Cancer Immunology (CIBCI) & Peter Gorer Department of Immunobiology, King's College London, London, United Kingdom; 3 The Francis Crick Institute, London, United Kingdom

**Keywords:** mass spectrometry, target-decoy, large search space, inSPIRE, immunopeptidomics, rescoring, Prosit, Percolator, CPU, central processing unit, FDR, false discovery rate, GPU, graphics processing unit, HCD, high collision–induced dissociation, HLA, human leukocyte antigen, inSPIRE, *in silico* Spectral Predictor Informed REscoring, iRT, indexed retention time, MS, mass spectrometry, PPV, positive predictive value, PR, precision–recall, PSM, peptide spectrum match, PTM, post-translational modification, VZV, varicella-zoster virus

## Abstract

Rescoring of mass spectrometry (MS) search results using spectral predictors can strongly increase peptide spectrum match (PSM) identification rates. This approach is particularly effective when aiming to search MS data against large databases, for example, when dealing with nonspecific cleavage in immunopeptidomics or inflation of the reference database for noncanonical peptide identification. Here, we present inSPIRE (*in silico* Spectral Predictor Informed REscoring), a flexible and performant open-source rescoring pipeline built on Prosit MS spectral prediction, which is compatible with common database search engines. inSPIRE allows large-scale rescoring with data from multiple MS search files, increases sensitivity to minor differences in amino acid residue position, and can be applied to various MS sample types, including tryptic proteome digestions and immunopeptidomes. inSPIRE boosts PSM identification rates in immunopeptidomics, leading to better performance than the original Prosit rescoring pipeline, as confirmed by benchmarking of inSPIRE performance on ground truth datasets. The integration of various features in the inSPIRE backbone further boosts the PSM identification in immunopeptidomics, with a potential benefit for the identification of noncanonical peptides.

## Highlights

• inSPIRE (*in silico* Spectral Predictor Informed REscoring).
• inSPIRE is a flexible and performant open-source rescoring pipeline.
• inSPIRE allows large-scale rescoring with multiple MS search files.
• inSPIRE can be applied to various search engines.
• inSPIRE has better performance than the original Prosit rescoring pipeline.

## Graphical Abstract

**Figure:**
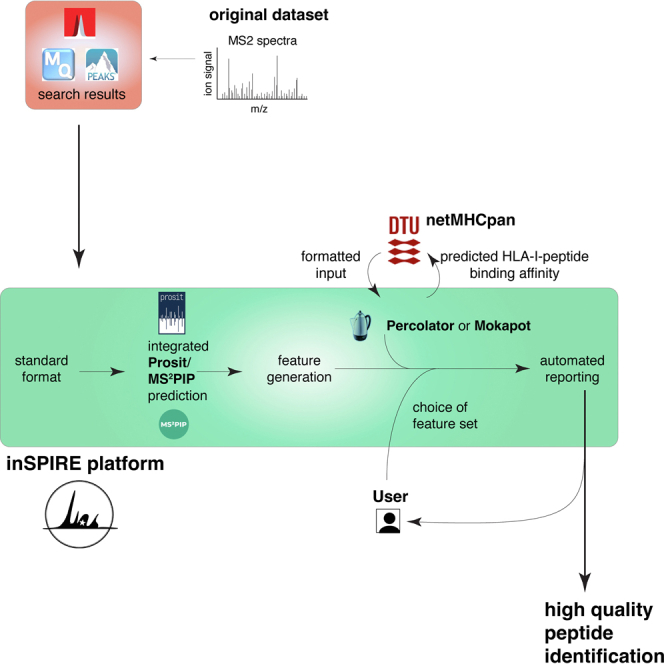

## In Brief

inSPIRE (*in silico* Spectral Predictor Informed REscoring) is a flexible and performant open-source rescoring pipeline built on Prosit or MS^2^PIP MS spectral prediction. inSPIRE is compatible with several search engines, allows large-scale rescoring with data from multiple MS search files, enables Prosit prediction without specialized GPU hardware, increases sensitivity to minor differences in amino acid residue position, and can be applied to various MS sample types, including tryptic proteome digestions, but is specifically optimized for immunopeptidomes.

Tandem mass spectrometry (MS) has been a successful tool for large-scale identification in proteomics and peptidomics (1, 2, 3). In tandem MS, peptides in a sample are first ionized and separated by their *m/z*, resulting in MS1 spectra. Selected ions are then fragmented, often by collision-induced dissociation or high collision–induced dissociation (HCD), resulting in MS2 spectra. Commonly, peptide identifications are performed *via* database search engines comparing the experimentally observed MS2 spectra to the theoretical fragments produced by all possible peptides in a reference proteome (*i.e.*, the search space) (4). The highest scoring match between theoretical and experimental spectra is then assigned to produce a peptide spectrum match (PSM). In order to quantify the probability that a PSM is correct, it is of importance to compute the false discovery rate (FDR). This is commonly estimated by searching a decoy database of reversed or randomized sequences, with a similar composition to the reference (or target) database (5). The decoy database should contain no true peptide sequences present in the analyzed sample, and so the number of false discoveries above a given scoring threshold can be estimated by the number of PSMs from the decoy database with scores above that threshold.

This approach is adopted by many traditional search engines, such as Mascot, MaxQuant, or PEAKS DB, and it has been successful, particularly when dealing with a small well-informed reference proteome (6). For example, in proteomics experiments, the digestion of a protein-containing sample with a specific protease such as trypsin results in a reduced search space compared with the digestion of the same sample with an unspecific protease. However, it is not always possible to achieve this reduced search space. One such case is the field of immunopeptidomics, where MS-mediated identifications of peptides presented by human leukocyte antigen class I and II (HLA-I and HLA-II) complexes can provide valuable insights into specific immune responses and potentially aid the development of targeted immunotherapies (7). These immunopeptides are generated within the cell *via* various processing steps, including proteasomal processing (8). Proteasomes are proteases that, in contrast to trypsin, can cleave after any amino acid, following complex dynamics (9, 10), thereby impacting on the database search space. The size of the search space can be expanded even further with increasing interest in noncanonical peptides outside the commonly used reference proteomes (11, 12, 13, 14). These enlarged search spaces can create a number of issues to traditional target-decoy search approaches, with a significant negative impact on both peptide yield and FDR estimation (15, 16).

Postprocessing or rescoring approaches, which perform additional validation on the target and decoy PSMs outputted from the original database search, have been developed to achieve high peptide yield at low FDR estimates, even when confronted with enlarged search spaces. These algorithms have been used for many years as a method of validating peptide identifications and increasing identification rates in MS search results (17, 18, 19). Percolator is one such algorithm that uses a semisupervised machine learning approach, which considers features beyond the original search engine score using Support Vector Machine models, and it has become the dominant approach for MS2 postprocessing (20). Percolator makes use of a subset of high-confidence target PSMs as positive samples and all decoy PSMs as negative samples for training its model. The trained model then provides a better separation between target and decoy peptides, allowing a larger number of peptides to be identified at a similar FDR. Throughout this process, Percolator employs a crossvalidation mechanism to avoid overfitting (21).

Percolator is highly flexible and allows its user to consider any set of features to describe a dataset of PSMs. Multiple researchers have taken advantage of this by integrating features from newly developed predictors to increase the number of high-confidence peptides identified. Such applications include the use of retention time predictors (22) and the use of predictors of HLA-peptide binding affinities in immunopeptidomics (23). One particularly fruitful approach has been the use of spectral predictors, as proposed by Silva *et al.* (24).

Accurate prediction of the MS2 spectrum of a peptide has been an active area of research for a number of years (25). While classic database search engines considered only the presence or the absence of possible MS2 fragment ions, that is, y- or b-ions for HCD, in an experimental MS2 spectrum, modern spectral predictors can accurately predict the relative intensities of the fragment ions (26, 27, 28, 29). One of the most significant accomplishments in this field is the Prosit spectral predictor (28, 29). Prosit is a deep learning tool that was trained on more than 20 million high-quality experimental MS2 spectra, and that has demonstrated state-of-the-art performance on both tryptic and immunopeptidomics datasets.

Wilhelm *et al.* (29) combined metrics describing the match between the Prosit-predicted spectrum and the experimentally observed spectrum as Percolator input features to increase discovery rates on immunopeptidome search spaces. Significant increases in performance have also been demonstrated for the MS^2^Rescore software (Compomics), which uses predictions from the MS^2^PIP spectral predictor rather than from Prosit (30). Both tools have also been compared when applied to a tryptic digest with an enlarged search space, showing a similar performance (31).

Despite the clear potential of using Prosit-predicted spectra in rescoring pipelines, its use is limited by the fact that no fully open-source pipeline is available. The Prosit rescoring pipeline presented by Wilhelm *et al.* (29) and recently updated (32) is only available *via* a web server, which only allows search results from a single MS RAW file and only processes search results from the MaxQuant search engine. Reproducibility is also limited with this system as no versioning information is available. The alternative INFERYS pipeline removes some of these technical limitations but is only available as part of the commercial Thermo Fisher Proteome Discoverer software (33).

**Fig. 1:**
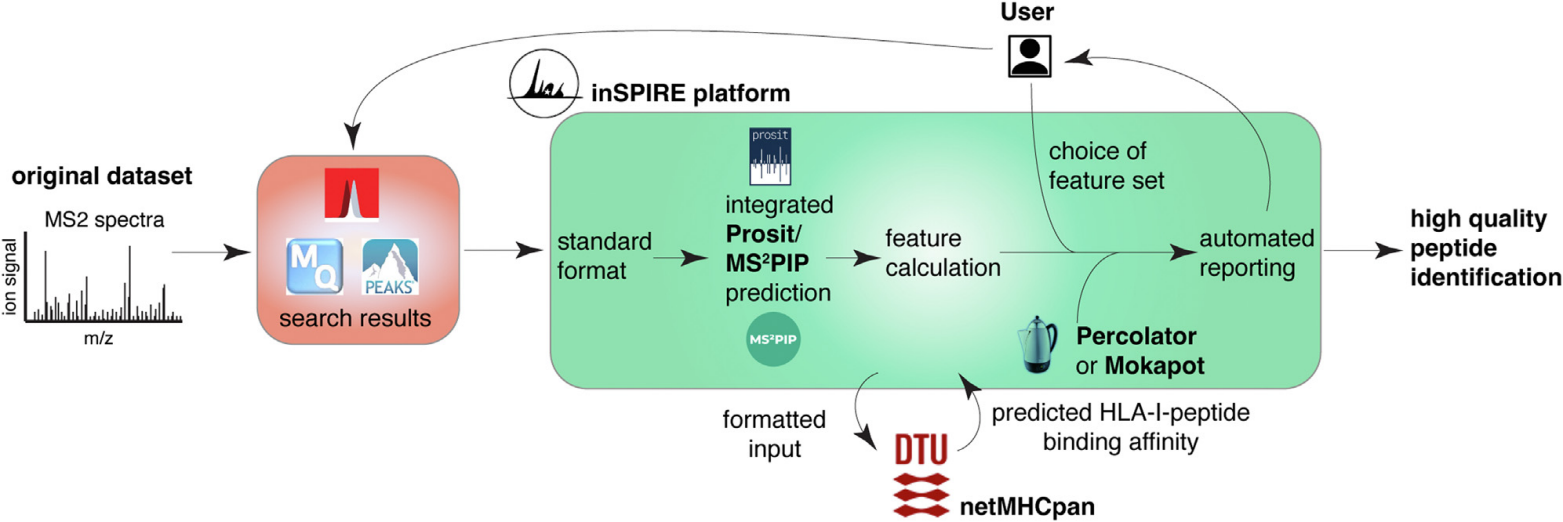
**Schematic of the inSPIRE pipeline.** Shown is an overview of the flow of execution for the inSPIRE pipeline. inSPIRE takes inputs from Mascot, PEAKS DB, or MaxQuant and interacts with Prosit, MS^2^PIP, and NetMHCpan, providing formatted inputs and using their respective predictions for rescoring. inSPIRE calls Percolator internally to execute the PSM rescoring. The user provides a configuration file for inSPIRE and must work with *Prosit* (and optionally NetMHCpan) to provide the predictions to inSPIRE. inSPIRE, *in silico* Spectral Predictor Informed REscoring; PSM, peptide spectrum match.

To address these gaps, we developed inSPIRE, which stands for *in silico* Spectral Predictor Informed REscoring. inSPIRE is a flexible and performant open-source rescoring pipeline, primarily built on Prosit retention time and MS spectral prediction. In contrast to the Prosit rescoring pipeline, inSPIRE is compatible with multiple major database search engines and allows large-scale rescoring with data from multiple search files. Furthermore, the inSPIRE pipeline can perform spectral prediction with Prosit on standard central processing unit (CPU) hardware, whereas the original Prosit release required a specialized graphics processing unit (GPU). For added flexibility, inSPIRE can also use MS^2^PIP as the spectral predictor instead of Prosit and Pyteomics for retention time prediction (34, 35), though this was not our primary focus given that the MS^2^Rescore pipeline already provides fully open-source rescoring using MS^2^PIP predictions. inSPIRE can be applied to various sample types including tryptic proteome digestions and immunopeptidomes. It is specifically optimized for enlarged immunopeptidome search spaces with increased sensitivity to minor differences in amino acid residue position (Fig. 1).

In addition, we developed an inSPIRE variant, specifically for HLA-I immunopeptidomics—that is, inSPIRE-affinity—that allows the integration of NetMHCpan (DTU Health Tech) predictions to the inSPIRE backbone. NetMHCpan also uses a deep learning framework, in this case, to predict the binding affinity of a given peptide to given HLA molecules (36, 37, 38). All details and information related to inSPIRE performance and the study outcomes are reported in supplemental Files S1-S11, supplemental Figs. S1–S14 and Tables S1–S4.

## Experimental Procedures

### Cell Lines

K562-B∗07:02 and K562-A∗02:01 cell line clones express either the single HLA-B∗07:02 or HLA-A∗02:01 alleles. They derive from the leukemia K562 cell line (American Type Culture Collection CCL-243), which does not express endogenous HLA-I and HLA-II molecules, and its generation and growing conditions are described elsewhere (13).

### HLA-I Immunopeptidome Elution and Tryptic Proteome Digestion

HLA-I-bound peptides were isolated from 10^9^ cells of K562-B∗07:02 and K562-A∗02:01 cell line clones, through HLA-I-peptide elution using W6/32 antibody, as described elsewhere (13). The MS files were already published (13).

The HLA-I immunopeptidomes used as training datasets were previously published by Paes *et al.* (39) and Bassani-Sternberg *et al.* (40).

Tryptic digestions of cell proteome obtained from the K562 cell line were carried out as follows: cell pellet was lysed in cell lysis buffer (50 mM Hepes, pH 7.5, 150 mM NaCl, 4% SDS, 2 mM DTT, and 0.5% NP-40) and heated at 95 °C for 10 min. The cell lysate was then diluted to a final concentration of 1% SDS with 50 mM Hepes, pH 7.5. Pierce Universal Nuclease (Thermo Fisher Scientific) was added according to the manufacturer’s recommendations and incubated at 37 °C for 30 min under shaking condition (300 rpm). Protein concentration was determined using Pierce BCA protein assay kit (Thermo Fisher Scientific), and 50 μg of protein was used for proteome digestion. Proteins were reduced with 5 mM DTT for 30 min at 37 °C and alkylated by the addition of 20 mM iodoacetamide and incubation for 30 min at room temperature in the dark. The reaction was quenched by incubation with 20 mM DTT for 15 min at room temperature before purification with SP3 beads (41) and elution for proteome digestion with trypsin (Promega) at protease to proteome weight ratio of 1:25 at 37 °C for 16 h.

### Synthetic Peptide Library

The synthetic peptide library contained 9, 10, or 15 amino acid long peptides (n = 6876 unique peptide sequences and 13,868 PSMs) related to CD4^+^ and CD8^+^ T-cell response to dengue virus and varicella-zoster virus (VZV), as described elsewhere (42). The dengue virus and VZV synthetic peptides utilized in this study were selected for analysis because they were already available in-house and synthesized for separate epitope identification studies. The selection and characterization of these peptides has been described previously (43, 44, 45, 46, 47, 48, 49, 50). Each of the peptides in synthetic peptide libraries was derived from respective dengue and VZV proteomes. Peptides were originally selected for other studies based on bioinformatic analyses of predicted capacity to bind various common HLA-I and HLA-II alleles in the general worldwide population. The set of dengue protein sequences of provenance represents all four dengue serotypes and several different variant isolates. The VZV peptides were primarily derived from the attenuated varicella vaccine strain vOka and a few variant isolates. Peptides were grouped in eight library batches, with each peptide measured at the concentration of 0.0625 pmol/μl. For each pool, 8 μl was injected in the instrument, thereby measuring 500 fmol of each peptide. The synthetic peptide libraries are reported in supplemental File S5.

### MS

MS data of HLA-I immunopeptidomes were collected using Orbitrap Fusion Lumos mass spectrometer coupled to an Ultimate 3000 RSLC nano pump (both from Thermo Fisher Scientific), as described elsewhere (13). The same method and instrument were used for the synthetic peptide library measurement. MS data of tryptic digestions of cell proteome were measured through Thermo Scientific Orbitrap Exploris 480 mass spectrometer. Digested proteome samples were injected using an Ultimate 3000 RSLC nano pump (both from Thermo Fisher Scientific). Briefly, 0.5 μg of each sample was loaded and separated by a nanoflow HPLC (RSLC Ultimate 3000) on an Easy-spray C18 nano column (30 cm length, 75 μm internal diameter). Peptides were eluted with a linear gradient of 5 to 45% buffer B (80% acetonitrile and 0.1% formic acid) at a flow rate of 300 nl/min over 58 min at 50 °C. The instrument was programmed within Xcalibur 3.1.66.10 (Thermo Fisher Scientific) to acquire MS data in a data-dependent acquisition mode using top 30 precursor ions. We acquired one full-scan MS spectrum at a resolution of 60,000 with a normalized automatic gain control target value of 300% and a scan range of 350 to 1600 *m/z*. The MS/MS fragmentation was conducted using HCD collision energy (28%) with an orbitrap resolution of 15,000. The normalized automatic gain control target value was set up at 100% with a maximum injection time of 40 ms. A dynamic exclusion of 22 s and 2 to 6 included charged states were defined within this method. The MS files used in each figure are reported in supplemental Table S4.

### MS Software Settings

For all sections where MaxQuant was used, RAW MS files were searched using MaxQuant GUI, version 1.6.17. First search peptide tolerance was set to 20 ppm, and the main search peptide tolerance was set to 4.5 ppm. Minimum peptide length was set to 7, and maximum peptide mass was set to 4600 Da. The mass tolerance for the fragment ions was set to 20 ppm. For identification, both PSM FDR and protein FDR were set to 1.0, allowing the maximum possible number of PSMs to be exported.

For the tryptic searches, we performed a specific search against the reference proteome with enzyme set to trypsin, allowing cleavage after proline. Up to two missed cleavages were allowed. Oxidation of methionine was set as the only variable post-translational modification (PTM), and carbamidomethylation of cysteine was set as a fixed modification. For the immunopeptidome searches, we performed an unspecific search against the reference proteome. Oxidation of methionine was set as the only variable PTM, and no fixed PTMs were set, with the exception of the datasets from Sarkizova *et al.* (51) where carbamidomethylation of cysteine was also set. Before rescoring with inSPIRE or Prosit-rescoring, all hits containing unmodified cysteine were removed as Prosit assumes carbamidomethylation of cysteine. For the ground truth dataset, a nonspecific search was used. In this case, no modifications were selected, and again, the hits containing unmodified cysteine residues were removed before rescoring with inSPIRE or Prosit-rescoring.

For the identification of the synthetic peptides used in the synthetic peptide library, we searched RAW files using PEAKS, version 10.6 with precursor mass tolerance of 5 ppm and fragment ion mass tolerance of 0.02 Da. No PTMs were allowed, and the results were exported at an FDR of 1%.

For the comparison between rescoring on different search engines, we searched RAW files using PEAKS, version 10.6 with precursor mass tolerance of 5 ppm and fragment ion mass tolerance of 0.02 Da. As with the MaxQuant searches, the tryptic searches were performed with two missed cleavages allowed, and cleavage was allowed after proline. The same PTM settings were used, and again, all hits containing cysteine were filtered out before rescoring. Results were exported for all PSMs with PEAKS −10lgP score greater than 0.

In the case of Mascot, we used Mascot Distiller, version 2.8.0.1 to process the MS RAW files. To allow the detection of chimeric spectra with Mascot, we set the maximum number of precursor *m/z* values to 2 and set *Allow multiple precursors per scan* to true. Precursor mass tolerance was set to 5 ppm, and fragment ion mass tolerance was set to 0.02 Da for both tryptic and immunopeptidome searches. The tryptic searches were performed with two missed cleavages allowed, and cleavage was allowed after proline. The same PTMs were allowed as with PEAKS and MaxQuant, and hits containing unmodified cysteine residues were filtered out before rescoring. We used Mascot’s automatic decoy search and exported both target and decoy results with a homology significance threshold set to 0.999999 (*i.e.*, exporting essentially all hits).

In order to provide the experimental spectra to the inSPIRE pipeline, RAW files were converted to mgf format using the msConvertGUI. The ThermoRawFileParser, version 1.4.0 was used to generate data for mgf input in Prosit-*delta* training pipeline (52).

Percolator, version 3.0.5 was for all rescoring jobs. All Prosit-rescoring jobs were submitted to the web server between March 10 and August 16, 2022. Rescoring with MS^2^Rescore was performed with version 2.1.2.

The search result files used for each figure are reported in supplemental Table S5. The final identifications for all pipelines for all datasets are provided in supplemental Files S6–S9.

### Application of MS^2^Rescore

For both tryptic and immunopeptidome datasets, the general settings for MS^2^Rescore were set with “pipeline” to “infer,” “feature_sets” to a list of “searchengine,” “ms2pip,” and “rt,” “run_percolator” to false, “id_decoy_patter” to null, “num_cpu” to −1, “config_file” to null, “tmp_path” to null, “mgf_path” to null, “output_filename” to null, “log_level” to info, and “plotting” to false. The “ms2pip” settings were set with “model” as “Immuno-HCD” for the immunopeptidome datasets and “HCD2021” for the tryptic datasets. The “frag_error” was set to 0.02. Variable modification of oxidation of methionine was set in either case with the “modification_mapping” set with “Oxidation (M)” mapping to “Oxidation” for both datasets. In the case of the tryptic proteome digestion, “fixed_modifications” was also set with “C” mapping to “Carbamidomethyl.”

### Application of Percolator

We reran Percolator for all pipelines because of the use of --subset-max-train command line argument in the Prosit rescoring pipeline. This command line argument can lead to a breakdown of the Percolator crossvalidation algorithm and should not be applied on small datasets according to The *et al.* (20), as confirmed by the Percolator team *via* GitHub (personal communication). We have communicated this issue to the Prosit team *via* GitHub. In reapplying Percolator with the same command line arguments to all pipelines, we ensured that the only variation in the PSMs identified by a rescoring pipeline was not because of different applications of Percolator.

MS^2^Rescore allows the user to select the command line arguments passed to Percolator, but for convenience, we simply reran Percolator on the .pin file produced by MS2Rescore *via* terminal with the same command line arguments used for inSPIRE and Prosit rescoring.

### RNA Sequencing and Reference Databases

The K562 RNA was extracted from K562 cell line pellets, processed for polyA enrichment, and sequenced by using NEBNext Ultra RNA Library Preparation Kit with random priming. Sequencing was performed using HiSeq 2x150 PE HO with a depth of 20 to 25 million reads per sample. Details about reads trimming, quantification, and data processing are described elsewhere (13).

The RNA sequencing dataset generated by Paes *et al.* (39) was generated as described in the original article and is available upon request to the authors.

RNA-informed reference databases were generated by imposing an expression cutoff of 10 estimated counts per transcript in Gencode transcriptome main annotation, release 33 (GRCh38.p13) (53). Protein-coding transcript translation sequences from these transcripts were kept in an RNA-informed reference database.

The Gencode transcriptome main annotation, release 33 (GRCh38.p13) (53) was searched alongside the RNA-informed reference database so that performance across different database size could be compared.

The UniProt *Homo Sapiens* proteome reference database used for PEAKS DB searches to generate PSMs for the Prosit-*delta* training data was downloaded on July 14, 2022.

### HLA-I-Peptide Binding Affinity Prediction

HLA-I-peptide binding affinity was predicted by applying NetMHCpan 4.1. Specifically, we used a custom Docker image. The NetMHCpan input file is provided as part of the inSPIRE “prepare” pipeline, provided that “useBindingAffinity” setting in the configuration file is specified as “asValidation” or “asFeature.” When using binding affinity predictions as a validation (*i.e.*, comparing number of predicted HLA-I-peptide binders for inSPIRE compared with Prosit-rescoring), we only considered NetMHCpan predictions for peptides with length between 8 and 14 residues because of software limitations. For inSPIRE-affinity, we generated predictions for all peptides as null values were not allowed in the Percolator input file.

For our validation and reporting pipelines, we defined a peptide predicted by NetMHCpan to bind a given HLA-I complex, by evaluating against the %Rank value, according to Reynisson *et al.* (37). The %Rank is a transformation on the original prediction, allowing comparison across HLA-I-peptide binding specificities. This system defined a “strong HLA-I binder” as a peptide with a %Rank <0.5% for a given HLA-I allele and a “HLA-I binder” as a peptide with a %Rank <2% for a given HLA-I allele.

We also used the values for the positive predictive value (PPV) reported by Reynisson *et al.* (37) as a metric to understand the variation between NetMHCpan performance on different alleles. Reynisson *et al.* (37) defined PPV as the number of positive binding peptides correctly predicted divided by 0.95 times the number of ligands predicted. By considering this metric for the different alleles analyzed, we could study how the strength of the NetMHCpan predictor for an HLA-I allele impacted the use of predicted HLA-I-peptide binding affinity both as an evaluation metric and as a feature for rescoring.

### Experimental Design and Statistical Rationale

This study aimed to benchmark inSPIRE performance against other state-of-the-art tools, in particular the Prosit rescoring pipeline, to demonstrate its value on datasets that the original Prosit rescoring pipeline could not allow rescoring, and to demonstrate the value of our novel Prosit-*delta* predictor.

In benchmarking, we focused our analysis on HLA-I immunopeptidome datasets of the K562-A∗02:01 and K562-B∗07:02 cell lines, for which we had an RNA-informed dataset and for which the NetMHCpan predictor performs strongly (37).

Since the Prosit web server only allowed the analysis of a single RAW file, and given that all the immunopeptidomics dataset came from previously published studies, we generally did not favor running multiple replicates of the same allele. This allowed us to explore a wider variety of HLA alleles with differing motifs rather than focusing on many replicates of a limited variety.

**Fig. 2:**
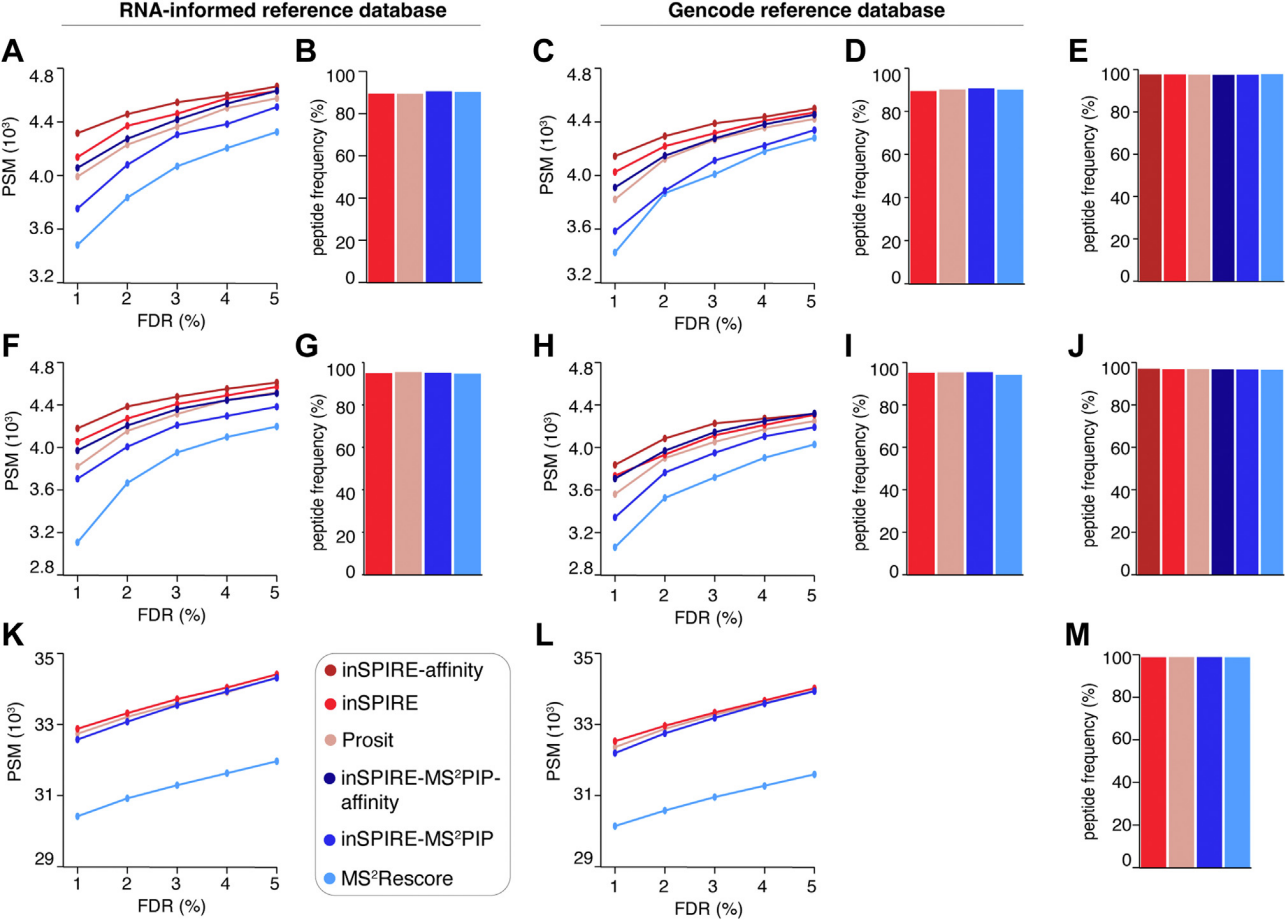
**PSM identification by inSPIRE compared with the Prosit rescoring pipeline on tryptic proteome digestions and HLA-I immunopeptidomes.***A*–*M*, analysis of the PSMs identified in HLA-I immunopeptidomes of K562-A∗02:01 (*A*–*E*) and K562-B∗07:02 (*F*–*J*) cell lines as well as the tryptic proteome digestion of the K562 cell line (*K*–*M*) by different pipelines. In (*A*, *B*, *F*, *G*, and *K*), the RNA-informed and in (*C*, *D*, *H*, *I*, and *L*) the full Gencode reference databases have been used. *A*, *C*, *F*, *H*, *K*, and *L*, number of PSMs identified by applying the pipelines on MaxQuant search results. *B*, *D*, *G*, and *I*, comparison of the percentage of identified peptides also predicted to bind the HLA-A∗02:01 (*B* and *D*) and HLA-B∗07:02 (*G* and *I*) complexes. Only peptides with lengths between 8 and 14 residues were included to allow the NetMHCpan HLA-I-peptide binding prediction. *E*, *J*, and *M*, the percentage of peptides identified by each pipeline on search results using the Gencode reference database, which were also found at any confidence level in the MaxQuant search of the RNA-informed database of the cognate cell line. HLA, human leukocyte antigen; inSPIRE, *in silico* Spectral Predictor Informed REscoring; PSM, peptide spectrum match.

In Figure 2, the MaxQuant search results of the K562-A∗02:01-derived immunopeptidome datasets searched with the RNA-informed and Gencode reference databases contained 14,689 and 15,065 PSMs, respectively. The equivalent datasets for the K562-B∗07:02-derived immunopeptidome contained 14,738 and 14,741 PSMs, respectively, and for the tryptic proteome digestion, they contained 55,396 and 56,560 PSMs, respectively.

**Fig. 3:**
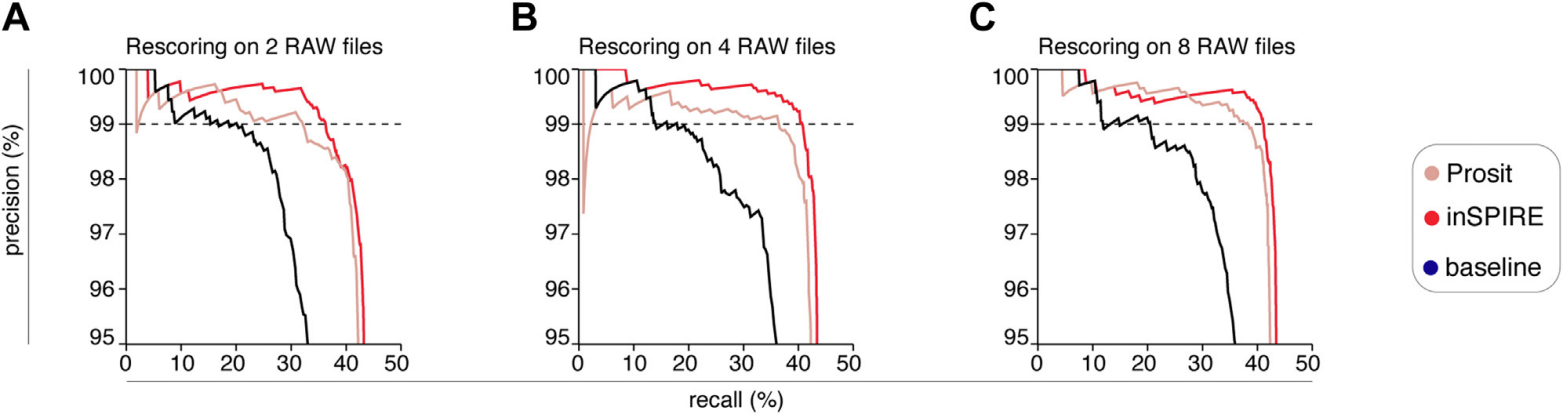
**Performance of inSPIRE compared with the Prosit rescoring pipeline on synthetic peptides’ ground truth dataset.***A*–*C*, shown is the precision (number of correctly identified peptides over number of identified peptides) against the recall (number of correctly identified peptides over number of correct peptides) of different rescoring pipelines. *A*, performance of different rescoring pipelines on ground truth data from two MS files (mean of 2108 target PSMs and 824 decoy PSMs). *B*, performance of different rescoring pipelines on ground truth data from four MS files (mean 4216 target PSMs and 1647 decoy PSMs). *C*, performance of different rescoring pipelines on ground truth data from eight MS files (8631 target PSMs and 3293 decoy PSMs). *Dash line* represents a precision of 0.99, which approximately corresponds to 1% FDR. FDR, false discovery rate; inSPIRE, *in silico* Spectral Predictor Informed REscoring; MS, mass spectrometry; PSM, peptide spectrum match.

In Figure 3, in all cases, the total number of PSMs used to generate the figures was 12,924 PSMs.

**Fig. 4:**
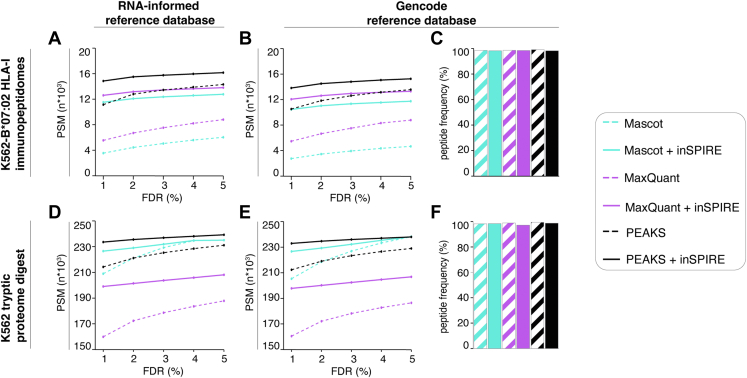
**inSPIRE increases the PSM yield preferentially benefiting MaxQuant searches.***A*–*F*, analysis of the PSMs identified in either the HLA-I immunopeptidomes of K562-B∗07:02 cell line (one biological and three technical replicates; *A*–*C*) or the tryptic proteome digestion of the K562 cell line (three biological and two technical replicates; *D*–*F*) by different pipelines. *A* and *D*, the RNA-informed and in (*B*, *C*, *E*, and *F*) the full Gencode reference databases have been used. *A*, *B*, *D*, and *E*, number of PSMs identified by the three search engines with or without inSPIRE rescoring. *C* and *F*, the percentage of peptides identified by each pipeline on search results using the Gencode reference database, which were also found at any confidence level in the MaxQuant search of the RNA-informed database for the two datasets. HLA, human leukocyte antigen; inSPIRE, *in silico* Spectral Predictor Informed REscoring; PSM, peptide spectrum match.

In Figure 4, the immunopeptidome rescoring for RNA-informed and Gencode reference database searches was based, respectively, on 41,188 and 40,929 PSMs for MaxQuant, 29,802 and 30,833 PSMs for Mascot, and 22,958 and 22,504 PSMs for PEAKS DB. The tryptic proteome digestion rescoring using the same reference databases was based, respectively, on 339,609 and 339,470 PSMs for MaxQuant, 244,697 and 244,698 PSMs for Mascot, and 316,095 and 310,219 PSMs for PEAKS DB. The *p* values relevant to supplemental Fig. S10 were calculated using Student’s *t* test.

**Fig. 5:**
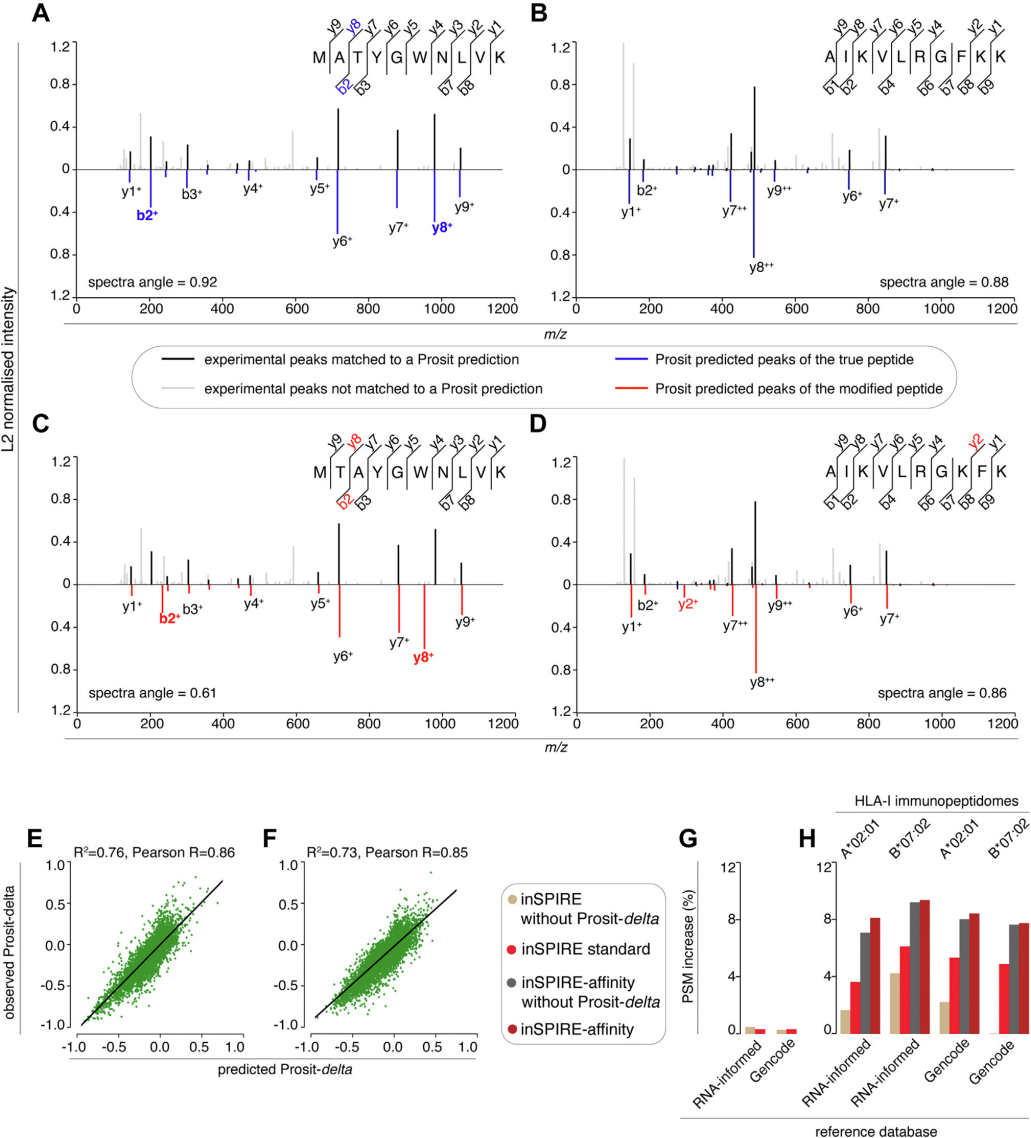
**Prosit-*delta* feature and its impact on inSPIRE’s PSM yield in HLA-I immunopeptidomics.***A*–*D*, Prosit-predicted MS2 spectra compared with experimentally measured MS2 spectra in representative cases wherein a switch of an amino acid residue pair results in either a large (*A* and *C*) or small (*B* and *D*) Prosit-*delta*. In each case, the pair plot is shown for the identified peptide (*A* and *B*) and for the peptide produced by the permutation of two adjacent amino acids in the original sequence (*C* and *D*). The peptide in (*A*) was identified in the synthetic peptide library sample SPL4-2 (scan number 11061). The peptide in (*B*) was identified in the synthetic peptide library sample SPL3-2 (scan number 15058). *E*, scatter plot of observed Prosit-*delta* values of PSMs in the search results of the K562-A∗02:01 and K562-B∗07:02 HLA-I immunopeptidomes against our model’s predictions. The data are downsampled to 10% of the original data to make the figure clearer. *F*, scatter plot of observed Prosit-*delta* values of PSMs in the search results of the K562 tryptic proteome digestion against our model’s predictions. The data are downsampled to 10% of the original data to make the figure clearer. *G* and *H*, relative increase in the number of identified PSMs by inSPIRE using different features as compared with Prosit rescoring pipeline. The analysis refers to the K562 tryptic proteome digestion (*G*) and the K562-B∗07:02 and K562-A∗02:01 HLA-I immunopeptidomes (*H*) searched by MaxQuant using either the RNA-informed or the full Gencode reference databases and 1% FDR. FDR, false discovery rate; HLA, human leukocyte antigen; inSPIRE, *in silico* Spectral Predictor Informed REscoring; PSM, peptide spectrum match.

The *R*^2^ values in Figure 5, *E* and *F* were based on 128,087 and 253,478 Prosit-*delta* values, respectively.

All analyses have been implemented in Python, if not stated otherwise. All statistics for performance measurement are described in the benchmarking framework.

### Metrics Validating Rescoring Performance

Our simplest analysis of the performance of an identification method was to compare the number of PSMs identified at 1% FDR as estimated *via* Percolator, which was used as the final identification method for all rescoring pipelines presented.

In an attempt to ensure that all pipelines were applying FDR estimation fairly, we used two independent validations for our K562, K562-A∗02:01, K562-B∗07:02 cell line datasets. First, for HLA-I immunopeptidome data, if NetMHCpan was not used in rescoring, we investigated the percentage of HLA-I binders and strong binders predicted by NetMHCpan among the peptides identified. Second, when rescoring search results obtained using the Gencode reference database, we investigated the percentage of peptides identified, which were also found by the search engine at any confidence level when searching the RNA-informed reference database.

We acknowledge that neither of these validation techniques was perfect; it is possible that a correct peptide sequence was not predicted to be an HLA-I binder by NetMHCpan or that the RNA sequencing evidence was not sufficient for its substrate protein to be included in our RNA-informed database. Equally, it is possible that an incorrectly identified peptide was predicted as a strong HLA-I binder and was also found in the RNA-informed database.

However, we estimated that reasonable consistency between these metrics across identifications from different pipelines, combined with Percolator’s well-established FDR estimation method (21), was a sufficient validation that an increased number of PSMs identified at a given threshold did represent better identification performance, indeed.

### Benchmarking With the Synthetic Peptide Library as Ground Truth Dataset

As a validation of the increased number of PSMs for the inSPIRE pipeline, we benchmarked all rescoring methods using “ground truth” datasets, in line with the benchmarking tool iBench (QSB lab) (54). In the approach applied in this study, we measured synthetic peptides *via* MS and selected MS2 scans that were identified with 1% FDR using PEAKS search engine. These peptides and their PSMs formed our “ground truth” dataset, although we note that our “ground truth” datasets represented an approximation to an absolute ground truth. Indeed, this strategy still had a minor degree of imperfection since it was based on MS measurement with 1% FDR rather than 0% FDR. Furthermore, although this strategy could contain a certain level of bias toward the PEAKS search engine, we estimated that this did not introduce any advantage for the rescoring methods used, and the use of synthetic peptide libraries greatly reduced the risk of identification errors.

We then embedded two-thirds of those synthetic peptide sequences into the Gencode reference database, thereby generating a constructed reference database, similarly as in the study of Mishto and collaborators (13). In this constructed reference database, these peptide sequences were labeled as the “discoverable.” We ensured that the remaining one-third of the synthetic peptide sequences were not in the constructed reference database, and, hence, were “undiscoverable.” We also added fragments of these peptide sequences to the constructed reference database so that the composition of the database was not biased against these peptides after their removal. We then searched the RAW files with MaxQuant using the constructed database and extracted all PSMs (FDR = 1.0%). Any identification found in the MaxQuant search results for an MS2 scan, which was not identified by PEAKS in the original search of the synthetic peptides, was filtered out before any rescoring was applied. This removed the possible confounding influence of contamination peptides of unknown origin.

In our study, we were initially limited by the fact that there were approximately 1000 peptides identified per RAW file, and the Prosit rescoring pipeline only allowed a single RAW file to be scored against. Hence, to overcome these potential limitations, we ran Prosit rescoring for each of the eight RAW files of the synthetic peptide libraries (*i.e.*, SPL1-2 to SPL8-2) separately. We then concatenated the *prosit.tab* files from each run, which contained all the inputs for the final Percolator rescoring, and reran Percolator with the concatenated files. We used the –override flag to ensure that Percolator used the full feature set for all executions.

In order to quantify the impact of the small dataset size on each pipeline, we performed rescoring on search results from two, four, and eight RAW files and calculated precision–recall (PR) curves for each method. To remove the effects of differences between RAW files, we ran all rescoring pipelines on four combinations of two RAW files and two combinations of four RAW files, so that in each case, the final performance was measured on the same data.

The PR curves were generated by varying the cutoff between the minimum and maximum Percolator score for each rescoring method. This involved combining Percolator scores across different runs. To note, Percolator normalized scores based on *q* value and combined scores internally from the different cross-validated models. Hence, combining scores across models did not create any clear bias between the methods being benchmarked.

The precision at each cutoff was calculated as the number of correctly identified PSMs divided by the total number of PSMs above the threshold, whereas the recall was calculated as the number of correctly identified PSMs divided by the number of discoverable peptides in the modified database. In each case, the maximum possible recall was limited by the number of correct PSMs found in the original search engine results.

### Development of inSPIRE Prosit-*delta* Predictor

The motivation for the Prosit-*delta* is explained in detail in the Results section. Briefly, we aimed to use a lightweight predictor to estimate the sensitivity of Prosit to adjacent residue permutation at each fragmentation site of the peptide. Although inSPIRE does allow for “brute force” computation of all Prosit-predicted MS2 spectra and resulting Prosit-*delta* values, this would result in doubling the run time and vastly increasing the memory consumption. As an alternative, we found that an xgboost Gradient Tree Boosting Regressor provided an appropriate and performant solution (55), without incurring the same computational burden.

We developed the *delta* predictor for Prosit only and not MS^2^PIP for a number of reasons. For instance, inSPIRE was primarily developed to increase the availability of Prosit predictions. Also, one of the main reasons why an inSPIRE user would choose the inSPIRE-MS^2^PIP pipeline could be to predict MS2 spectra for peptides containing PTMs not available with Prosit. Including a broad range of PTMs—particularly those linked to the termini of a peptide—would significantly complicate the current version of the *delta* predictor. In addition, spectral angle was not the primary metric on which MS^2^PIP was trained, although it was an important feature in the inSPIRE-MS^2^PIP pipeline. Hence, the development of *delta* scoring within MS^2^PIP might need a specific investigation of the best metric to be considered.

In order to generate training data for the Prosit-*delta* predictor, we searched the HLA-I immunopeptidomes of Paes *et al.* (39) and Bassani-Sternberg *et al.* (40) using PEAKS 10.6, using an RNA-informed database and the UniProt Homo Sapiens database, respectively, and exported all hits with PEAKS -10lgP greater than 0. These data were combined with synthetic immunopeptides used by Wilhelm *et al.* (29) for which we used the MaxQuant identifications provided in their PRIDE repository. A full description of the RAW files used and the number of PSMs are provided in supplemental Table S2. The PEAKS DB searches were run with oxidation of methionine and carbamidomethylation of cysteine set as variable modifications. All hits containing unmodified cysteines were discarded before training. The PSMs were then divided between train (80% of the data) and test (20% of the data) ensuring that there was no overlap in the peptides used between train and test.

While we collected data for peptides with lengths 7 to 30 and precursor charge 1 to 6, the vast majority of our training comes from peptides of length less than 13 and precursor charge 1 to 3 (supplemental Fig. S11, *A* and *B*). This feature was in agreement with one of the main objectives of the Prosit-*delta* predictor, which was its application to immunopeptidome datasets. We also show sequence logo plots for the peptides of length 8 to 11 residues in the combined dataset in supplemental Fig. S11, *C*–*F*, thereby illustrating that the dataset was not biased toward any specific motif.

For each PSM, we selected five positions at random in the peptide sequence and generated Prosit predictions of MS2 spectra for the peptide created by flipping the adjacent amino acids at those positions. The target variable was the difference between the spectral angle of the modified sequence and the spectral angle of the original sequence. Hence, each PSM in the training dataset generated five training data points. The features used as input for the Prosit-*delta* predictor are detailed in supplemental Table S3.

We performed hyperparameter tuning on the parameters minimum child weight, maximum tree depth, learning rate, gamma, and columns sampled by tree. We then used randomized search with five-fold cross-validation on the training set and compared performance for different sets of hyperparameters based both on predictive performance (*r*^2^ score) and speed of execution. The results of this first round of hyperparameter tuning are shown in supplemental File S10. We then selected five sets of hyperparameters, which performed well in crossfold validation and which were then trained on the full training data and evaluated on the test set (supplemental File S11). From this second round of evaluation, it was clear that the model with maximum tree depth 16, minimum child weight 2, learning rate 0.15, gamma 0.1, and columns sampled by tree 0.9 was the most performant model. This model showed the best performance on the test data (*r*^2^ score = 0.74) despite showing slightly lower performance on the train data.

The trained model was packaged within inSPIRE, and the minimum, maximum, median, first quartile, third quartile, fraction of predicted Prosit-*deltas* above −0.1 and fraction of predicted Prosit-*deltas* above 0.0 were passed as features for Percolator.

As with the inSPIRE source code, all the training the code for the Prosit-*delta* predictor is fully open source. Hence, a user of inSPIRE could retrain this predictor on their own data and use their Prosit-*delta* model in the inSPIRE pipeline.

### inSPIRE Implementation and Application

All inSPIRE jobs presented in this study may be recreated by providing the required inSPIRE configuration file. Full details on the creation of the inSPIRE config file may be found in the README available on GitHub. For each experiment, “rescoreMethod” was set to “percolator,” and “mzAccuracy” was set to 0.02. The search engine and location of search results as well as the location of scan data converted to either mgf or mzML was provided *via* the config file. For inSPIRE-affinity, “useBindingAffinity” was set to “asFeature.” The calibrated collision energy was also set, which agreed between inSPIRE and the Prosit web server in all cases.

To generate Prosit predictions without specialized GPU hardware, we downloaded the Prosit model details and changed the definition of the CuDNNGRU layers in the model.yml file to GRU layers with the following settings, activation equal to tanh, recurrent_activation equal to sigmoid, unroll equal to false, use_bias equal to true, and reset_after equal to true. We also had to avoid using the Tensorflow graph as in the original Prosit code. We were then able to reload the model definition and weights using Tensorflow (Google Brain team), version 2.5 and execute predictions by modifying the open-source code available from the Prosit team (see https://github.com/kusterlab/prosit).

For timing comparisons of Prosit prediction on CPU against GPU, spectral prediction on CPU was run on Intel Sky Lake processors, whereas the GPU predictions were run on an NVIDIA Tesla K40m Graphics Card.

All Prosit spectral predictions were generated using the 2020 HCD model and indexed retention time (iRT) predictions using the 2019 model. For users who have very large datasets and easy access to GPU servers, we also provide the modified version of the original Prosit code, including a converted Singularity image so that Prosit can be run on a high-performance computing cluster, a change to the MSP export code so that Prosit predicted iRT values were included, and an option so that *m/z* values of all fragment ions were not calculated by Prosit. We found it was much more efficient to calculate the *m/z* values of the fragment ions in the inSPIRE pipeline and greatly reduce the required prediction time, particularly if a large number of predicted spectra were required.

## Results

### inSPIRE

We developed inSPIRE to be a flexible rescoring pipeline, which provides the power of Prosit prediction for users without specialized computational hardware and can be applied to a vast number of tandem MS proteomics datasets generated with HCD or collision-induced dissociation fragmentation. Although it is optimized for HLA-I immunopeptidomics, inSPIRE can also be applied to standard proteomics experiments. inSPIRE provides flexibility through compatibility with commonly used search engines, that is, MaxQuant, PEAKS, or Mascot, as well as compatibility with open data formats, that is, mgf and mzML formats. For HLA-I immunopeptidomics, the inSPIRE-affinity variant can be employed, which allows integration of NetMHCpan predictions of HLA-I-peptide binding affinity and potentially others in future releases.

When using Prosit, inSPIRE is subject to the limitations of the Prosit predictor and will filter out PSMs where the peptides are of length less than 7 or greater than 30. If the sample contains unmodified cysteines (noncarbamidomethylated) or variable modifications other than the oxidation of methionine, these PSMs will be filtered out by inSPIRE. Unmodified cysteines and a wider range of variable modifications are supported if the user selects MS^2^PIP as their spectral predictor (inSPIRE-MS^2^PIP supports a maximum of nine unique modifications). However, we did not prioritize the development of the MS^2^PIP pipeline given that there already exists a fully open-source rescoring pipeline, which utilizes MS^2^PIP prediction in MS^2^Rescore.

inSPIRE provides multiple pipelines to fulfill different user requirements. The core functionality is provided *via* the “core” pipeline (though individual steps may be run independently), which enables MS2 spectral rescoring (Fig. 1). The first subsection of the “core” functionality, “prepare,” formats the search engine output for Prosit or MS^2^PIP (and NetMHCpan if required). The required Prosit predictions or MS^2^PIP predictions are then generated *via* the “predictSpectra” pipeline. For Prosit, this entailed the conversion of the GPU-only models available from the Prosit team to a version that could be run on an ordinary CPU (see for Tool Implementation and statistical analysis details). We found that execution of the “predictSpectra” pipeline on the CPU was effective, and timing even compared favorably with execution of the original Prosit code when we removed the calculation of *m/z* values for all possible fragment ions (supplemental Fig. S1). We also validated that the predictions from the inSPIRE CPU implementation did not differ from the online Prosit model by running the spectral prediction pipelines for both tools on 13,054 unique peptide-charge combinations (the peptides identified by MaxQuant in the HLA-A∗02:01 immunopeptidome). We found that the predicted iRT values and MS2 spectra agreed to near machine single precision with a mean spectral angle between predicted spectra of 0.9999997 of a mean difference in iRT of the order of 10^−5^ (supplemental File S2).

NetMHCpan prediction is not currently integrated within inSPIRE because of license restrictions, but if the user wishes to employ the inSPIRE-affinity variant, they could generate the predictions independently (see instructions in the README on GitHub and supplemental File S1). The final part of the inSPIRE core pipeline, “rescore” utilizes all available data for improved rescoring. This process generates all required features from search results, spectral predictions, and NetMHCpan-predicted binding affinities. Once all features are generated, inSPIRE calls Percolator to rescore the PSMs. These results are then benchmarked against Percolator rescoring without spectral features, and an HTML report is provided to the user (see the examples provided in supplemental File S3). This report provides details of varying feature importance, feature distributions, and performance of the inSPIRE pipeline against Percolator with classical features (Fig. 1). If inSPIRE-affinity was used, or binding affinity was selected as a validation technique, this report also shows the percentage of NetMHCpan-predicted binders that are identified.

In addition to the core functionality, inSPIRE also provides a calibration pipeline, which allows calibration of the collision energy setting passed to Prosit. The inSPIRE calibration pipeline is a simple pipeline as described by Wilhelm *et al.* (29), where the highest scoring unmodified PSMs are considered based on search engine score, and spectral angles against Prosit predictions for each collision energy between 20 and 40 (inclusive) are generated. The collision energy that provides the highest mean spectral angle is selected as the recommended collision energy setting for further analysis.

In inSPIRE, we have introduced a number of changes to the feature set and feature selection approaches compared with other spectral rescoring pipelines such as Prosit rescoring and MS^2^Rescore. For example, rather than providing features matching y- and b-ions, we distinguished between the dominant ion series (the series with greater predicted coverage) and the lesser ion series. While this is unlikely to impact tryptic proteome digestion datasets, where the y-series is generally dominant, we found it a more useful distinction for HLA-I immunopeptidome rescoring, where there is more variation in which ion series is dominant. We also found that considering *m/z* error on the MS2 fragment ions was a useful feature. We provide a full description of all features used by inSPIRE in supplemental Table S1.

Compared with other pipelines, another major change was the use of features from a Prosit-*delta* predictor (see the Experimental Procedures section). In our ground truth datasets, we found that swapping the position of certain pairs of adjacent amino acids in the true peptide sequence led to a very small change in the PSM spectral angle. We termed this change the “Prosit-*delta*.” In early development of inSPIRE, peptides that had a small Prosit-*delta* were often misassigned in the ground truth datasets, resulting in incorrect (although similar) peptide sequences. While generating Prosit-predicted spectra and spectral angles for swaps of every pair of adjacent amino acid residues would massively increase the computational load of the pipeline, we aimed to use a less intensive predictor to estimate the sensitivity of Prosit at each fragmentation site of the peptide. Therefore, the aim of including these Prosit-*delta* predictor features was to identify the sensitivity of the Prosit MS2 spectral prediction to minor changes in amino acid residue positions. These *delta* predictions are not available for the inSPIRE-MS^2^PIP pipeline (see the Experimental Procedures section for full details on the technical aspects of the Prosit-*delta* predictor).

In addition to its aforedescribed flexibility, inSPIRE provides several options to allow for manual feature inclusion or exclusion by the user. For example, if the user had a very small dataset where some features in the standard inSPIRE feature set could lead to the introduction of bias, they can simply add a list of “excludeFeatures” to the inSPIRE config file. Furthermore, if the user was particularly interested in certain sequence identifications and wished to examine their MS2 spectra more closely, inSPIRE provides a plotting tool, which generates pair plots in pdf format and compare the experimental MS2 spectrum to the Prosit-predicted MS2 spectrum. An example of these plots for PSMs of varying quality is provided in supplemental File S4. All that is required is to select the rows of interest from the inSPIRE final assignments or provide a csv file with the peptides of interest along with their source file and scan number. This functionality may be of particular interest to users who wish to use inSPIRE, for example, for epitope target discovery in immunopeptidomics.

### inSPIRE Boosts PSM Identifications in HLA-I Immunopeptidomes and Tryptic Proteome Digestions

We focused our initial benchmarking of inSPIRE against the Prosit rescoring pipeline and the MS^2^Rescore pipeline with MaxQuant search results as well as comparing it with a baseline rescoring without the use of spectral prediction. For comparison between the pipelines, we attempted to provide as fair a comparison between tools as possible; thereby, we reran the final Percolator rescoring with the same command line arguments used for all pipelines (see the Experimental Procedures section for details). However, one area of difference, which we could not correct for, was the fact that the current release of MS^2^Rescore dropped PSMs with duplicate scan numbers, meaning that chimeric spectra could not be discovered. This feature might be changed in the next release of MS^2^Rescore (personal communication), when we would expect an increase in PSMs identified.

We applied all pipelines—that is, Prosit rescoring, inSPIRE, inSPIRE-affinity, MS^2^Rescore, inSPIRE-MS^2^PIP, and inSPIRE-MS^2^PIP-affinity—to HLA-I immunopeptidomes derived from K562-A∗02:01 (Fig. 2, *A*–*E* and supplemental Fig. S2, *A* and *B*) and K562-B∗07:02 (Fig. 2, *F*–*J* and supplemental Fig. S2, *C* and *D*) cell lines and tryptic proteome digestions derived from K562 cell lines (Fig. 2, *K*–*M*). Since the Prosit rescoring web server allowed only a single MS file per search, we analyzed a single MS file for both HLA-I immunopeptidomes (Fig. 2, *A*–*J*) and tryptic proteome digestions (Fig. 2, *K*–*M*).

In our initial MaxQuant analysis, we used a reference database informed by RNA sequencing of K562 cell lines, which consisted of 43,578 entries. Then, we repeated the analysis using the full Gencode reference database, which consisted of 100,551 entries. This strategy allowed evaluation of the impact of the reference database size on the PSM yield of inSPIRE compared with the other pipelines in the range of estimated FDRs 1 to 5% (Fig. 2, *A*–*M*). We focused our analysis of the peptides identified on PSMs identified at 1% FDR as this is the most commonly employed FDR threshold in recent proteomics and immunopeptidomics studies (Fig. 2 and supplemental Figs. S2–S5).

It has already been demonstrated that the Prosit rescoring pipeline and MS^2^Rescore significantly increase PSM yield over baseline rescoring without spectral prediction (29, 30). Similarly, we observed a significant impact of inSPIRE, with more than a 150% increase in PSMs discovered at 1% FDR for all immunopeptidome datasets as compared with the baseline rescoring (supplemental Fig. S2).

For rescoring pipelines using spectral prediction applied to HLA-I immunopeptidomes, inSPIRE identified a slightly higher number of PSMs (4–6% increase) compared with the Prosit Rescoring pipeline, and inSPIRE-affinity showed the highest PSM yield (8–9% increase on the Prosit Rescoring pipeline). The increase in PSMs identified between Prosit Rescoring and inSPIRE using MS^2^PIP (3–6% increase) was similar to the increase of inSPIRE over Prosit Rescoring. In each case, MS^2^Rescore identified the fewest PSMs at 1% FDR (Fig. 2, *A*, *C*, *F* and *H*). In the case of the immunopeptidome dataset, this difference was unlikely to be explained entirely by the dropping of chimeric MS2 spectra; it may be more related to the fact that MS^2^Rescore uses 100 features in its rescoring as opposed to the 40 features used by Prosit Rescoring and the 41 to 42 features used by inSPIRE and inSPIRE-affinity. This larger feature set may be less suitable when rescoring small immunopeptidome datasets as there is a greater risk of overfitting with a large number of features and a smaller dataset, leading to a reduced number of PSMs identified when cross-validation is applied within Percolator. In contrast to the performances on HLA-I immunopeptidomes, the performance of inSPIRE, Prosit Rescoring, and inSPIRE-MS^2^PIP was very similar on the tryptic proteome digestion dataset using both RNA-informed and full Gencode reference databases, with a marginal improvement in PSM yield by inSPIRE over Prosit Rescoring and a marginal increase by Prosit Rescoring over inSPIRE-MS^2^PIP (Fig. 2, *K* and *L*). Again, fewer PSMs were identified by MS^2^Rescore, although, in this case, the difference could almost entirely be explained by the removal of chimeric MS2 spectra in the MS^2^Rescore pipeline. The number of unique scans identified at 1% FDR was very similar for all pipelines, with all identifying approximately 30,000 unique scans.

To validate the assignments of each pipeline, we initially computed the percentage of peptides, identified at 1% FDR for each pipeline, which were predicted to bind the cognate HLA-I allele by NetMHCpan among the peptides identified in the HLA-I immunopeptidomes. This percentage was high and similar across all pipelines (Fig. 2, *B*, *D*, *G* and *I*). As a second validation step, we computed the percentage of peptides, identified using the Gencode reference database by each pipeline, which were also identified using RNA-informed reference database. The analysis of these metrics also pointed toward a reliable peptide identification in both HLA-I immunopeptidomes and tryptic proteome digestions (Fig. 2, *E*, *J* and *M*).

By examining the incremental PSMs discovered by competing pipelines, that is, the PSMs exclusively discovered by one pipeline but not the other, we observed greater variation in these two validation metrics. We performed such “head-to-head” analysis for inSPIRE against baseline rescoring (supplemental Fig. S2), inSPIRE against Prosit Rescoring (supplemental Fig. S3), inSPIRE-affinity against inSPIRE (supplemental Fig. S4), and inSPIRE-MS^2^PIP against MS^2^Rescore (supplemental Fig. S5). The best performance on each metric was invariably observed in the pool of PSMs shared between pipelines. However, the incremental PSMs from the pipeline that identified the greater number of PSMs at 1% FDR generally showed higher values for the two validation metrics over the competing pipeline that identified fewer PSMs. Overall, we found that peptides exclusively identified by inSPIRE variants showed a higher percentage of peptides predicted to be HLA-I binders compared with those peptides that were exclusively identified by the baseline, Prosit rescoring, and MS^2^Rescore. Furthermore, in the latter comparisons, peptides exclusively identified by inSPIRE variants using the Gencode reference database were more frequently identified using RNA-informed reference database (supplemental Figs. S2, S3 and S5). Only two exceptions broke this homogenous pattern: (i) the percentage of peptides predicted to be HLA-I binders in the K562-B∗07:02 HLA-I immunopeptidomes using RNA-informed reference database comparing inSPIRE against Prosit rescoring pipeline (supplemental Fig. S3*C*); (ii) the percentage of peptides identified using the Genecode reference database that were also identified using the RNA-informed reference database in the K562-B∗07:02 HLA-I immunopeptidomes comparing inSPIRE-MS^2^PIP against MS^2^Rescore (supplemental Fig. S5*B*). These exceptions may indicate a level of noise in our validation metrics (see the caveats described in the Experimental Procedures section). However, overall, the evidence across all incremental PSM comparisons (supplemental Figs. S2–S5) and pipelines (Fig. 2) indicated a consistent quality in the PSMs identified by Percolator at 1% FDR for each pipeline.

In addition to these independent metrics, we also examined MS2 coverage and spectral angle distribution for the incremental PSMs discovered by competing pipelines (supplemental Figs. S2–S5), which could provide some insight into the features prioritized by each pipeline. Not surprisingly, we found that the PSMs identified by inSPIRE only had significant higher spectral angle distribution compared with the baseline rescoring pipeline, which does not use features from Prosit (supplemental Fig. S2). Furthermore, PSMs exclusively identified by inSPIRE but not Prosit Rescoring typically had a greater MS2 coverage but a lower spectral angle than those exclusively identified by Prosit Rescoring but not by inSPIRE (supplemental Fig. S3). In our comparison of inSPIRE-affinity to the standard inSPIRE pipeline, we noted that the incremental PSMs identified by inSPIRE-affinity showed higher mean spectral angle and MS2 coverage over inSPIRE standard, despite the added importance of binding affinity (supplemental Fig. S4). Therefore, inSPIRE-affinity did not only identify peptides with higher HLA-I-peptide binding affinities but also with overall better spectral features. This suggested that the MS2 spectral and HLA-I-peptide binding affinity prediction features worked effectively in concert rather than one aspect being solely prioritized over the other. The MS^2^Rescore pipeline did not compute spectral angle between the experimental and MS^2^PIP predicted MS2 spectra. Therefore, it should not come as a surprise that the mean spectral angle was greater for the PSMs identified exclusively by inSPIRE-MS^2^PIP than for PSMs identified exclusively by MS^2^Rescore (supplemental Fig. S5).

To study the impact of inSPIRE on PSM yield as compared with Prosit rescoring on a wide variety of HLA-I alleles, we performed rescoring on 12 monoallelic HLA-I cell lines from the large HLA-I immunopeptidome dataset published by Sarkizova *et al.* (51). For this analysis, we focused on acquiring data using diverse HLA-I alleles and included datasets where peptide sequence motifs were less well understood (*e.g.*, the HLA-G alleles). This strategy allowed testing of the effect of inSPIRE-affinity in such challenging settings. Overall, we found that the resulting peptide sequence motifs from Prosit rescoring compared with inSPIRE rescoring were extremely similar (supplemental Fig. S6). However, with regard to peptide identification, we observed a 0.1 to 7.6% increase (mean = 3.1%) in PSMs identified at 1% FDR with the inSPIRE pipeline over the Prosit rescoring pipeline and 0.6 to 10.6% increase (mean = 4.2%) over Prosit rescoring when using the inSPIRE-affinity pipeline (supplemental Fig. S7).

We then compared the percentage of peptides predicted by NetMHCpan to be either binders or strong binders of the cognate HLA-I complex and identified at 1% FDR across different HLA-I alleles with a broad range of NetMHCpan performance (supplemental Fig. S8). In this analysis, the variation in the percentage of peptides predicted to be HLA-I binders was larger between HLA-I alleles than between pipelines. In addition, in those HLA-I alleles for which NetMHCpan prediction reported a low NetMHCpan’s PPV, that is, where the HLA-I-peptide binding affinity was not efficiently predicted by NetMHCpan, inSPIRE-affinity showed a similar percentage of peptides predicted to be HLA-I binders than the other pipelines. This further indicates that inSPIRE-affinity did not blindly assign peptides based on predicted HLA-I-peptide binding affinity alone, particularly when the HLA-I-peptide binding affinity prediction was less reliable.

### inSPIRE Shows High Specificity and Stable Performance on Ground Truth Datasets of Varying Size

Although the validation analyses performed so far suggested a high performance of inSPIRE and inSPIRE-affinity, we wished to further verify that the increased PSM yield observed by applying inSPIRE pipelines was due to an improved sensitivity of inSPIRE compared with the other pipelines, rather than the result of spurious identifications. To this end, we applied inSPIRE, Prosit Rescoring, and the baseline rescoring to ground truth datasets of synthetic peptide libraries of pathogen-derived 9, 10, and 15 amino acid long peptides (supplemental File S5). The ground truth dataset construction followed the approach described by Cormican *et al.* (54) and is explained in the Experimental Procedures section. The pipelines’ benchmarking on a ground truth dataset containing PSMs with characteristics similar to HLA-I immunopeptidomes could let us estimate the precision—that is, number of correctly identified peptides over the number of identified peptides—and recall—that is number of correctly identified peptides over the number of correct peptides—of a given method. The computation of PR is a standard strategy for performance evaluation of binary predictors and has also been applied to proteomics in other contexts (56, 57). Optimal performance in terms of PR would show a tool achieving close to the maximum possible recall while maintaining high precision until very low scoring thresholds lead to a steep drop. The maximum possible recall for each rescoring pipeline was the fraction of the true PSMs correctly identified by the initial search engine at any identification cutoff. Hence, a lower limit on the recall indicates that there were more incorrect assignments in the original database search.

Within the immunopeptidomics field, we observed that implementing Percolator with a standard feature set on small datasets could lead to a lower precision (13). Therefore, we tested inSPIRE performance in ground truth datasets with increasing size, from a mean of just under 3000 total PSMs using two RAW files to over 12,000 PSMs from eight RAW files (Fig. 3). To remove the effects of different performance on different RAW files, we performed rescoring on four sets of two RAW files (Fig. 3*A*), two sets of four RAW files (Fig. 3*B*), and a single set of eight RAW files (Fig. 3*C*) and calculated PR across all sets (see Experimental Procedures section for more details). In the case of inSPIRE, we observed stable performance on all ground truth datasets, even when rescoring was performed on a small number of PSMs (Fig. 3*A*).

For the baseline rescoring, we observed very similar performance no matter the size of the dataset, achieving 19 to 20% recall at 99% precision for any dataset size. The pipelines using spectral prediction saw a steady increase in performance with dataset size. The Prosit rescoring pipeline increased from 32% recall at 99% precision when rescoring on two RAW files (Fig. 3*A*), to 36% recall at 99% precision when rescoring on four RAW files (Fig. 3*B*), to 38% recall at 99% precision when rescoring on all eight RAW files (Fig. 3*C*). Similarly, with inSPIRE, at 99% precision, we observed the recall of 36% when rescoring on two RAW files (Fig. 3*A*), 40% when rescoring on four RAW files (Fig. 3*B*), and 41% when rescoring on eight RAW files (Fig. 3*C*). Therefore, under all conditions, we observed a performance improvement of inSPIRE over Prosit rescoring (Fig. 3, *A*–*C*), which was in line with the increase in PSMs observed on the HLA-I immunopeptidome datasets (Fig. 2). Hence, the results on the ground truth datasets provided further validation of the results on the HLA-I immunopeptidome datasets. To note, both Prosit-rescoring and inSPIRE obtained on average 98% precision at their respective estimated 1% FDRs across all datasets, indicating a slight underestimation of the FDR for both tools in these experimental conditions (Fig. 3, *A*–*C*).

### inSPIRE Is Performant on Larger Scale Datasets and Across Search Engines

In contrast to Prosit rescoring pipeline, inSPIRE supports multiple MS files in a single run and can be combined with various database search engines (Fig. 1). To estimate how inSPIRE would perform on results from larger datasets—for example, derived from multiple MS files—and different search engines, we tested inSPIRE on larger datasets of HLA-I immunopeptidomes and tryptic proteome digestions than those investigated in Figure 2. Indeed, we applied inSPIRE to search results from three MS files of K562-B∗07:02-derived HLA-I immunopeptidomes (Fig. 4, *A* and *B*). As reference database, we use both RNA-informed and Gencode reference databases, thereby evaluating the impact of the reference database size on inSPIRE’s PSM yield. Rescoring with inSPIRE increased the PSM yield at 1% FDR for all search engines by 31 to 33% for PEAKS DB, 225 to 281% for Mascot, and 120 to 127% for MaxQuant compared with the baseline Percolator rescoring. Interestingly, the larger increase in PSMs using inSPIRE with PEAKS DB and MaxQuant was observed when using the RNA-informed rather than Gencode reference database. The best performance came from the rescoring of PEAKS DB search results with a 15 to 18% increase over MaxQuant results (Fig. 4, *A* and *B*).

As with the performance using a single technical replicate (Fig. 2, *E*, *J* and *M*), we observed a high and comparable percentage of peptides identified at 1% FDR when searching the Gencode reference database, which were also found in the search results of the RNA-informed reference database, with a minimum of 98.2% for Mascot search results after rescoring with inSPIRE and a maximum of 99.0% for the PEAKS DB baseline (Fig. 4*C*).

We performed the same analysis on six MS files from K562 cell tryptic proteome digestions using MaxQuant, Mascot, and PEAKS DB. inSPIRE rescoring improved the PSM yield of all search engines compared with the baseline Percolator rescoring, which was even more pronounced than the HLA-I immunopeptidome datasets. As with the HLA-I immunopeptidome datasets, the best identification rate was achieved with inSPIRE rescoring of PEAKS DB search results (Fig. 4, *D* and *E*).

While the percentage of peptides identified using the Gencode reference database identified also using the RNA-informed reference database was high and consistent for results with Mascot and PEAKS with and without rescoring, a slight decrease of this percentage was observed by applying the inSPIRE rescoring to MaxQuant results (from 98.9% to 97.2% of identified peptides; Fig. 4*F*).

The most remarkable variation in search engine performance from HLA-I immunopeptidome to tryptic proteome digestion searches came from Mascot. Indeed, this search engine identified the fewest PSMs on the HLA-I immunopeptidome datasets although showed performance similar to PEAKS DB on the tryptic proteome digestion search results (Fig. 4, *A*, *B*, *D* and *E*). This is in line with results obtained with other approaches (13).

More generally, inSPIRE rescoring was particularly impactful relative to the original search engine choice in the enlarged HLA-I immunopeptidome search space. Indeed, rescoring of MaxQuant search results in the tryptic proteome digestion search space still provided fewer identifications than PEAKS DB and Mascot baseline results. In opposite, in the HLA-I immunopeptidome results, even Mascot, the lowest performing search engine in this case, identified more PSMs after rescoring than PEAKS DB without rescoring at 1% FDR (Fig. 4, *A* and *B*).

To understand the impact of the search engines on the pool of identified peptides, we analyzed the overlap among peptides identified using the three search engines with and without inSPIRE (supplemental Fig. S9). In the HLA-I immunopeptidome dataset, we observed that inSPIRE rescoring led to a particularly large increase in the number of shared identified peptides among the search engines (supplemental Fig. S9, *A* and *B*). In the tryptic proteome digestion dataset, the impact was less striking since even without inSPIRE rescoring, the majority of the identified peptides were discovered by all three search engines (supplemental Fig. S9, *C* and *D*).

Furthermore, we investigated the impact of MS1 intensity on the ability of the search engines and inSPIRE rescoring to identify PSMs in HLA-I immunopeptidomes (supplemental Fig. S10, *A*–*C*) and in tryptic proteome digestions (supplemental Fig. S10, *D*–*F*). In HLA-I immunopeptidomes, PSMs identified by inSPIRE only showed significantly lower MS1 intensity distributions compared with PSMs identified by both inSPIRE and the search engines and inSPIRE. This suggested that the use of inSPIRE allowed the detection of lower intensity PSMs in HLA-I immunopeptidomics (supplemental Fig. S10, *A*–*C*). Such differences were, however, absent when analyzing tryptic proteome digestion samples (supplemental Fig. S10, *D*–*F*).

### Insight into inSPIRE Optimization of Spectral Prediction Features by Modeling Amino Acid Pair Switch (Prosit-*delta*)

Beyond some improvements to the feature set and the integration of NetMHCpan predictions, the inSPIRE pipeline employs a novel approach to PSM rescoring, namely the prediction of the sensitivity of the Prosit MS2 spectrum prediction in case of switch of adjacent amino acid residue pairs. This switch (or permutation) had previously been noted by Collaert *et al.* (15) as a difference that traditional search engines struggled to detect. This sensitivity, or lack thereof, is represented in the examples of Prosit MS2 spectrum prediction of the peptides MATYGWNLVK and AIKVLRGFKK identified in the synthetic peptide library samples (Fig. 5, *A* and *B*). For these peptides, we challenged Prosit MS2 spectrum prediction by switching the position of two adjacent amino acids and computed the difference in the spectral angle between the true and the modified peptides, which we named “Prosit-*delta*” value. In the case of the peptide MATYGWNLVK, the position switch between alanine (A) and threonine (T) in the true peptide MATYGWNLVK, which resulted in the modified peptide MTAYGWNLVK, led to a large Prosit-*delta* value, with the spectral angle dropping from 0.92 for the original sequence to 0.61 for the modified sequence (Fig. 5*C*). In contrast, for the peptide AIKVLRGFKK, the switch in position between the phenylalanine (F) and lysine (K), which resulted in the theoretical peptide AIKVLRGKFK, led to a small Prosit-*delta* value (spectral angle drops from 0.88 to 0.86) as we saw only minor differences in predicted MS2 spectra between the original and the modified peptides (Fig. 5*D*).

In early development of inSPIRE, we noticed that misassigned peptide sequences in the synthetic peptides’ ground truth datasets often occurred when a similar peptide sequence was found in the constructed reference database (data not shown); in particular, this often happened when a peptide sequence differed from the true peptide sequence without impacting on the spectral angle, that is, with a small Prosit-*delta* (see representative example in Fig. 5, *B* and *D*). Hence, we hypothesized that the distribution of these Prosit-*delta* values for each position in the sequence could be a useful feature in rescoring, and that sequences where the Prosit spectral angle was less sensitive to minor changes in amino acid position should be assigned with less confidence than those where the spectral angle was more sensitive. To avoid the heavy computational burden of generating Prosit-predicted spectra for all modified sequences, we developed a model to predict the Prosit-*delta* values as described in the Experimental Procedures section. To make the model as independent as possible of the datasets benchmarked with inSPIRE, we used large publicly available HLA-I immunopeptidome data from Paes *et al.* (39) and Bassani-Sternberg *et al.* (40), as well as the synthetic immunopeptide dataset used to train the Prosit model (29) as training and test data (supplemental Table S2). Peptide length and charge state distribution of the training data reflected those distributions typically observed in HLA-I immunopeptidome datasets (supplemental Fig. S11, *A* and *B*). The peptide sequence motifs in the training dataset were evenly distributed, thereby confirming that we were not training the Prosit-*delta* predictor on peptides that were biased toward some specific sequence motifs (supplemental Fig. S11, *C*–*F*).

The resulting Prosit-*delta* predictor was an ensemble learning–based model, which primarily focused on the local features of the permutation site, although further features such as precursor charge state were also considered (supplemental Fig. S12). Interestingly, collision energy was one of the most important features, which highlights the need for careful calibration of Prosit before usage. The full feature set used by the Prosit-*delta* predictor is described in detail in supplemental Table S3. Application of the trained Prosit-*delta* predictor to K562-A∗02:01 and K562-B∗07:02 HLA-I immunopeptidomes and tryptic proteome digestion dataset resulted in good performance (Fig. 5, *E* and *F*).

To understand the impact of these Prosit-*delta* predictions on inSPIRE performance, we rescored the search results of the HLA-I immunopeptidome and tryptic proteome digestion (Fig. 2 and supplemental Figs. S2–S4) with the Prosit-*delta* features excluded. We found that the Prosit-*delta* features had little to no impact when applied to the tryptic proteome digestion datasets, where the enzyme specificity reduced the search space and made the search engine more sensitive to changes in sequence (Fig. 5*G*). On the HLA-I immunopeptidomes, the Prosit-*delta* implementation had an impact when the RNA-informed reference database was used, although the most impact was observed when the Gencode reference database was used (Fig. 5*H* and supplemental Fig. S13). For the search of the K562-B∗07:02 HLA-I immunopeptidome using the Gencode reference database, the increase in PSM yield over Prosit rescoring was almost entirely because of the Prosit-*delta* features (Fig. 5*H*). As with the tryptic proteome digestions, the Prosit-*delta* features had little impact when applied to inSPIRE-affinity (Fig. 5*H*). This could be explained by the fact that peptide sequence motifs driving the HLA-I-peptide–binding motifs—and, hence, the HLA-I-peptide binding affinity prediction—are already very sensitive to minor changes in peptide sequence. Therefore, in inSPIRE-affinity, the impact of Prosit-*delta* features might be attenuated by the impact of HLA-I-peptide–binding affinity prediction.

To validate these latter results, we again analyzed the percentage of peptides predicted by NetMHCpan to be HLA-I binders as well as the percentage of peptides identified using the Gencode reference database that were also identified using the RNA-informed reference database. As observed in the previous analyses, the various inSPIRE pipelines resulted in a high and comparable peptide percentage (supplemental Fig. S14), which hinted toward a reliable peptide identification.

## Discussion

The integration of MS2 spectral prediction with rescoring strategies is a fruitful area to boost MS identification performance and could find in the inSPIRE pipeline a versatile, high-performing, user-friendly, and open-source tool. The ability of inSPIRE to generate Prosit predictions on a standard CPU architecture significantly lowers the entry barrier for researchers, thereby “bringing Prosit to the people.” The standard implementation of inSPIRE has demonstrated similar performance to the Prosit Rescoring pipeline for search results of tryptic proteome digestions and improved performance for HLA-I immunopeptidomes. The integration of NetMHCpan prediction of HLA-I-peptide–binding affinity in inSPIRE-affinity pipeline raises the performance even further by optimizing inSPIRE for the analysis of HLA-I immunopeptidomes. In this study, the increased PSM identification rate of inSPIRE over the Prosit rescoring and MS^2^Rescore pipelines has further been validated by investigation of the peptides identified. We have observed consistency in the percentage of identified peptides predicted to bind to the cognate HLA-I complex and the percentage of peptides identified from the Gencode reference database that were validated by RNA sequencing evidence. Furthermore, we have demonstrated the improved recall of inSPIRE over Prosit rescoring at 99% precision on ground truth datasets. In comparison to Prosit rescoring, the inSPIRE pipeline also brings significant benefits in terms of data volume and flexibility across multiple search engines. We have demonstrated that inSPIRE can provide increased PSM identification rates in each of these scenarios. The ability to apply inSPIRE to PEAKS DB, in particular, allows for significant improvement over rescoring of MaxQuant search results. Finally, we provide a detailed documentation and a step-by-step user guide to achieve easy access to inSPIRE for both the coding-experienced and coding-inexperienced user.

The addition of the Prosit-*delta* predictor boosts identification rates and can open potential avenues for other features based on meta-analysis, that is, features considering not only the match between experimental and predicted MS2 spectrum of a given peptide but also the uniqueness and sensitivity of the prediction. These features showed a greater impact on analysis confronted with larger search spaces such as the full Gencode database. This suggests the Prosit-*delta* features may assist with the challenges raised by the expansion of database size through the increased interest in noncanonical peptide identification in proteomics and immunopeptidomics. For example, the impact of the database size on method performance has been demonstrated for post-translational spliced peptides (13), and spectral prediction features as a solution for the search space size problem in proteogenomics have been proposed (31).

As suggested by Verbruggen *et al.* (31), we found the spectral prediction features far more impactful when dealing with the larger immunopeptidome search space compared with tryptic proteome digestion search spaces. Interestingly, this was not always the case when comparing results from the full Gencode reference database to the results from the RNA-informed reference databases. In fact, the increase in PSMs identified by applying inSPIRE and Prosit rescoring compared with the baseline, which was observed on the K562-B∗07:02 HLA-I immunopeptidomes, showed to be larger when the RNA-informed reference database rather than the full Gencode reference database was used. This could point toward a limitation on the potential improvement in peptide yield on rescoring, when the true peptide is not selected by the original search engine at any confidence threshold, and, hence, cannot be found through rescoring. This limitation may also contribute to the lack of variation between inSPIRE and Prosit Rescoring pipelines for the search results of the tryptic proteome digestions. In this case, the number of MS2 scans left unidentified in the rescored search results was similar to the number of decoy hits in the search results. Hence, any great increase in identification rate on the tryptic proteome digestion datasets would have to be treated with a certain degree of suspicion.

In conclusion, we speculate that the application of rescoring pipelines using MS2 spectral features will become the standard approach to tackle large search space problems in proteogenomics. We, here, provide a fully open-source tool, inSPIRE, which can aid flexible MS analysis pipeline development in a user-friendly manner in the future.

## Data and Software Availability

Our MS proteomics data have been deposited to the ProteomeXchange Consortium *via* the PRIDE (58) partner repository with the dataset identifiers PXD031709 (13), PXD031812 (54), and PXD034056.

The MS proteomics data published by Paes *et al.* (39) are available at the PRIDE repository with the dataset identifier PXD015489.

The RNA sequencing data used for the analysis of our MS proteomics data have been deposited in the National Center for Biotechnology Information Sequence Read Archive database with the accession code PRJNA721129 (13).

The inSPIRE software has been implemented with Python and is available at GitHub (https://github.com/QuantSysBio/inSPIRE).

The Prosit-*delta* training software has been implemented with Python and is available at GitHub (https://github.com/QuantSysBio/prosit-delta).

The modified version of the Prosit spectral prediction code is also available at GitHub (https://github.com/QuantSysBio/qsb-modified-prosit).

The models downloaded by the inSPIRE pipeline at run time have been deposited on figshare (https://figshare.com/articles/software/inSPIRE_Models/20368035).

The RNA sequencing data generated by Peas *et al.* (39) are available upon request to the authors.

Analyses were carried out in Python 3.8.

Figures have been generated in Python using the Plotly library and Logomaker for the sequence logo plots (59). Postprocessing was done with Adobe Illustrator, version 26.2.

MS analysis was carried out with MaxQuant, version 1.16.17, Mascot, version 2.7.01, and PEAKS X Pro 10.6. Rescoring was carried out with Percolator, version 3.0.5. Preprocessing of MS RAW files for Mascot was performed with Mascot-Distiller, version 2.8.0.1, and RAW files were converted to mgf/mzML format for inSPIRE input using ms-convert GUI (ProteoWizard, version 3.0.9134) and ThermoRawFileParser, version 1.4.0, for input in Prosit-*delta* training pipeline (52).

## Supplemental data

This article contains supplemental data.

## Conflict of interest

The authors declare no competing interests.

## References

[1] Abelin J.G., Keskin D.B., Sarkizova S., Hartigan C.R., Zhang W., Sidney J. (2017). Mass spectrometry profiling of HLA-associated peptidomes in mono-allelic cells enables more accurate epitope prediction. Immunity.

[2] Aebersold R., Mann M. (2016). Mass-spectrometric exploration of proteome structure and function. Nature.

[3] Ouspenskaia T., Law T., Clauser K.R., Klaeger S., Sarkizova S., Aguet F. (2022). Unannotated proteins expand the MHC-I-restricted immunopeptidome in cancer. Nat. Biotechnol..

[4] Verheggen K., Raeder H., Berven F.S., Martens L., Barsnes H., Vaudel M. (2020). Anatomy and evolution of database search engines-a central component of mass spectrometry based proteomic workflows. Mass Spectrom. Rev..

[5] Kall L., Storey J.D., MacCoss M.J., Noble W.S. (2008). Posterior error probabilities and false discovery rates: two sides of the same coin. J. Proteome Res..

[6] Cravatt B.F., Simon G.M., Yates J.R. (2007). The biological impact of mass-spectrometry-based proteomics. Nature.

[7] Caron E., Kowalewski D.J., Chiek Koh C., Sturm T., Schuster H., Aebersold R. (2015). Analysis of major histocompatibility complex (MHC) immunopeptidomes using mass spectrometry. Mol. Cell. Proteomics.

[8] Barbosa C.R.R., Barton J., Shepherd A.J., Mishto M. (2021). Mechanistic diversity in MHC class I antigen recognition. Biochem. J..

[9] Liepe J., Holzhutter H.G., Bellavista E., Kloetzel P.M., Stumpf M.P., Mishto M. (2015). Quantitative time-resolved analysis reveals intricate, differential regulation of standard- and immuno-proteasomes. Elife.

[10] Mishto M., Liepe J., Textoris-Taube K., Keller C., Henklein P., Weberruss M. (2014). Proteasome isoforms exhibit only quantitative differences in cleavage and epitope generation. Eur. J. Immunol..

[11] Mansurkhodzhaev A., Barbosa C.R.R., Mishto M., Liepe J. (2021). Proteasome-generated cis-spliced peptides and their potential role in CD8(+) T cell tolerance. Front. Immunol..

[12] Goodenough E., Robinson T.M., Zook M.B., Flanigan K.M., Atkins J.F., Howard M.T. (2014). Cryptic MHC class I-binding peptides are revealed by aminoglycoside-induced stop codon read-through into the 3' UTR. Proc. Natl. Acad. Sci. U. S. A..

[13] Mishto M., Horokhovskyi Y., Cormican J.A., Yang X., Lynham S., Urlaub H. (2022). Database search engines and target database features impinge upon the identification of post-translationally cis-spliced peptides in HLA class I immunopeptidomes. Proteomics.

[14] Ruiz Cuevas M.V., Hardy M.P., Holly J., Bonneil E., Durette C., Courcelles M. (2021). Most non-canonical proteins uniquely populate the proteome or immunopeptidome. Cell Rep..

[15] Colaert N., Degroeve S., Helsens K., Martens L. (2011). Analysis of the resolution limitations of peptide identification algorithms. J. Proteome Res..

[16] Krug K., Carpy A., Behrends G., Matic K., Soares N.C., Macek B. (2013). Deep coverage of the Escherichia coli proteome enables the assessment of false discovery rates in simple proteogenomic experiments. Mol. Cell. Proteomics.

[17] Kall L., Canterbury J.D., Weston J., Noble W.S., MacCoss M.J. (2007). Semi-supervised learning for peptide identification from shotgun proteomics datasets. Nat. Methods.

[18] Ma K., Vitek O., Nesvizhskii A.I. (2012). A statistical model-building perspective to identification of MS/MS spectra with PeptideProphet. BMC Bioinformatics.

[19] Searle B.C. (2010). Scaffold: a bioinformatic tool for validating MS/MS-based proteomic studies. Proteomics.

[20] The M., MacCoss M.J., Noble W.S., Kall L. (2016). Fast and accurate protein false discovery rates on large-scale proteomics data sets with percolator 3.0. J. Am. Soc. Mass Spectrom..

[21] Granholm V., Noble W.S., Kall L. (2012). A cross-validation scheme for machine learning algorithms in shotgun proteomics. BMC Bioinformatics.

[22] Giese S.H., Sinn L.R., Wegner F., Rappsilber J. (2021). Retention time prediction using neural networks increases identifications in crosslinking mass spectrometry. Nat. Commun..

[23] Bichmann L., Nelde A., Ghosh M., Heumos L., Mohr C., Peltzer A. (2019). MHCquant: automated and reproducible data analysis for immunopeptidomics. J. Proteome Res..

[24] Silva A.S.C., Bouwmeester R., Martens L., Degroeve S. (2019). Accurate peptide fragmentation predictions allow data driven approaches to replace and improve upon proteomics search engine scoring functions. Bioinformatics.

[25] Elias J.E., Gibbons F.D., King O.D., Roth F.P., Gygi S.P. (2004). Intensity-based protein identification by machine learning from a library of tandem mass spectra. Nat. Biotechnol..

[26] Degroeve S., Martens L. (2013). MS2PIP: a tool for MS/MS peak intensity prediction. Bioinformatics.

[27] Degroeve S., Maddelein D., Martens L. (2015). MS2PIP prediction server: compute and visualize MS2 peak intensity predictions for CID and HCD fragmentation. Nucleic Acids Res..

[28] Gessulat S., Schmidt T., Zolg D.P., Samaras P., Schnatbaum K., Zerweck J. (2019). Prosit: proteome-wide prediction of peptide tandem mass spectra by deep learning. Nat. Methods.

[29] Wilhelm M., Zolg D.P., Graber M., Gessulat S., Schmidt T., Schnatbaum K. (2021). Deep learning boosts sensitivity of mass spectrometry-based immunopeptidomics. Nat. Commun..

[30] Declercq A., Bouwmeester R., Hirschler A., Carapito C., Degroeve S., Martens L. (2022). MS^2^Rescore: data-driven rescoring dramatically boosts immunopeptide identification rates. Mol. Cell. Proteomics.

[31] Verbruggen S., Gessulat S., Gabriels R., Matsaroki A., Van de Voorde H., Kuster B. (2021). Spectral prediction features as a solution for the search space size problem in proteogenomics. Mol. Cell. Proteomics.

[32] Gabriel W., The M., Zolg D.P., Bayer F.P., Shouman O., Lautenbacher L. (2022). Prosit-TMT: deep learning boosts identification of TMT-labeled peptides. Anal. Chem..

[33] Zolg D.P., Gessulat S., Paschke C., Graber M., Rathke-Kuhnert M., Seefried F. (2021). INFERYS rescoring: boosting peptide identifications and scoring confidence of database search results. Rapid Commun. Mass Spectrom..

[34] Goloborodko A.A., Levitsky L.I., Ivanov M.V., Gorshkov M.V. (2013). Pyteomics--a Python framework for exploratory data analysis and rapid software prototyping in proteomics. J. Am. Soc. Mass Spectrom..

[35] Levitsky L.I., Klein J.A., Ivanov M.V., Gorshkov M.V. (2019). Pyteomics 4.0: five years of development of a Python proteomics framework. J. Proteome Res..

[36] Jurtz V., Paul S., Andreatta M., Marcatili P., Peters B., Nielsen M. (2017). NetMHCpan-4.0: improved peptide-MHC class I interaction predictions integrating eluted ligand and peptide binding affinity data. J. Immunol..

[37] Reynisson B., Alvarez B., Paul S., Peters B., Nielsen M. (2020). NetMHCpan-4.1 and NetMHCIIpan-4.0: improved predictions of MHC antigen presentation by concurrent motif deconvolution and integration of MS MHC eluted ligand data. Nucleic Acids Res..

[38] Nielsen M., Lundegaard C., Blicher T., Lamberth K., Harndahl M., Justesen S. (2007). NetMHCpan, a method for quantitative predictions of peptide binding to any HLA-A and -B locus protein of known sequence. PLoS One.

[39] Paes W., Leonov G., Partridge T., Chikata T., Murakoshi H., Frangou A. (2019). Contribution of proteasome-catalyzed peptide cis-splicing to viral targeting by CD8(+) T cells in HIV-1 infection. Proc. Natl. Acad. Sci. U. S. A..

[40] Bassani-Sternberg M., Chong C., Guillaume P., Solleder M., Pak H., Gannon P.O. (2017). Deciphering HLA-I motifs across HLA peptidomes improves neo-antigen predictions and identifies allostery regulating HLA specificity. PLoS Comput. Biol..

[41] Hughes C.S., Moggridge S., Muller T., Sorensen P.H., Morin G.B., Krijgsveld J. (2019). Single-pot, solid-phase-enhanced sample preparation for proteomics experiments. Nat. Protoc..

[42] Gutman I., Gutman R., Sidney J., Chihab L., Mishto M., Liepe J. (2022). Predicting the success of Fmoc-based peptide synthesis. ACS Omega.

[43] Li S., Sullivan N.L., Rouphael N., Yu T., Banton S., Maddur M.S. (2017). Metabolic phenotypes of response to vaccination in humans. Cell.

[44] Chiu C., McCausland M., Sidney J., Duh F.M., Rouphael N., Mehta A. (2014). Broadly reactive human CD8 T cells that recognize an epitope conserved between VZV, HSV and EBV. PLoS Pathog..

[45] Weiskopf D., Angelo M.A., Grifoni A., O'Rourke P.H., Sidney J., Paul S. (2016). HLA-DRB1 alleles are associated with different magnitudes of dengue virus-specific CD4+ T-cell responses. J. Infect. Dis..

[46] Weiskopf D., Angelo M.A., de Azeredo E.L., Sidney J., Greenbaum J.A., Fernando A.N. (2013). Comprehensive analysis of dengue virus-specific responses supports an HLA-linked protective role for CD8+ T cells. Proc. Natl. Acad. Sci. U. S. A..

[47] Weiskopf D., Angelo M.A., Bangs D.J., Sidney J., Paul S., Peters B. (2015). The human CD8+ T cell responses induced by a live attenuated tetravalent dengue vaccine are directed against highly conserved epitopes. J. Virol..

[48] Weiskopf D., Cerpas C., Angelo M.A., Bangs D.J., Sidney J., Paul S. (2015). Human CD8+ T-cell responses against the 4 dengue virus serotypes are associated with distinct patterns of protein targets. J. Infect. Dis..

[49] Weiskopf D., Bangs D.J., Sidney J., Kolla R.V., De Silva A.D., de Silva A.M. (2015). Dengue virus infection elicits highly polarized CX3CR1+ cytotoxic CD4+ T cells associated with protective immunity. Proc. Natl. Acad. Sci. U. S. A..

[50] Weiskopf D., Angelo M.A., Sidney J., Peters B., Shresta S., Sette A. (2014). Immunodominance changes as a function of the infecting dengue virus serotype and primary versus secondary infection. J. Virol..

[51] Sarkizova S., Klaeger S., Le P.M., Li L.W., Oliveira G., Keshishian H. (2020). A large peptidome dataset improves HLA class I epitope prediction across most of the human population. Nat. Biotechnol..

[52] Hulstaert N., Shofstahl J., Sachsenberg T., Walzer M., Barsnes H., Martens L. (2020). ThermoRawFileParser: modular, scalable, and cross-platform RAW file conversion. J. Proteome Res..

[53] Frankish A., Diekhans M., Ferreira A.M., Johnson R., Jungreis I., Loveland J. (2019). GENCODE reference annotation for the human and mouse genomes. Nucleic Acids Res..

[54] Cormican J.A., Soh W.T., Mishto M., Liepe J. (2022). iBench: a ground truth approach for advanced validation of mass spectrometry identification method. Proteomics.

[55] Chen T.Q., Guestrin C. (2016). Kdd'16: Proceedings of the 22nd ACM SIGKDD International Conference on Knowledge Discovery and Data Mining.

[56] Collatz M., Mock F., Barth E., Holzer M., Sachse K., Marz M. (2021). EpiDope: a deep neural network for linear B-cell epitope prediction. Bioinformatics.

[57] Cox J., Hein M.Y., Luber C.A., Paron I., Nagaraj N., Mann M. (2014). Accurate proteome-wide label-free quantification by delayed normalization and maximal peptide ratio extraction, termed MaxLFQ. Mol. Cell. Proteomics.

[58] Perez-Riverol Y., Csordas A., Bai J., Bernal-Llinares M., Hewapathirana S., Kundu D.J. (2019). The PRIDE database and related tools and resources in 2019: improving support for quantification data. Nucleic Acids Res..

[59] Tareen A., Kinney J.B. (2020). Logomaker: beautiful sequence logos in Python. Bioinformatics.

