# Supplementary material for: inSPIRE: An Open-Source Tool for Increased Mass Spectrometry Identification Rates Using Prosit Spectral Prediction
Source: Mol Cell Proteomics. 2022 Oct 21;21(12):100432. doi: 10.1016/j.mcpro.2022.100432 (PMC9720494; doi:10.1016/j.mcpro.2022.100432)
Supplement: Supplemental File S1 [file mmc1.pdf]

**File S1. inSPIRE README converted to pdf.** The inSPIRE README provides detailed instructions on how to run the software and the configuration options available to the user. This README is also available from the inSPIRE repository on GitHub as a markdown file.

# inSPIRE

in silico Spectral Predictor Informed REscoring

inSPIRE allows easy rescoring of MaxQuant, Mascot or PEAKS DB search results using spectral prediction. inSPIRE is primarily developed to use Prosit predicted spectra but can also use MS2PIP predictions. inSPIRE enables the prediction of MS2 spectra with Prosit without the need of a GPU and it can be run on a standard workstation or laptop.

## Set Up

### Before Downloading

For MacOS you will require Miniforge. For Linux or Windows users, any version of conda will suffice.

### Download

You can then clone the repo from your terminal with:

```
git clone https://github.com/QuantSysBio/inSPIRE.git
```

and navigate in your terminal to the inSPIRE folder with:

```
cd inSPIRE
```

### Setting up your environment:

Once you have installed conda and cloned the repo

1) To start with create a new conda environment with python version 3.9:

```
conda create --name inspire python=3.9
```

2) Activate this environment

```
conda activate inspire
```

2) You will then need to install the inspire package:

```
python setup.py install
```

4) To check your installation, run the following command (it is normal for this call to hang for a few seconds on first execution)

```
inspire -h
```

Once you have successfully installed inSPIRE you should run it specifying your pipeline and a config file. The core execution of inSPIRE will take the form:

```
inspire --config_file path-to-config-file --pipeline pipeline-to-execute
```

where the config file is a yaml file specifying details of the inSPIRE execution and the pipeline is one of the options described below.

## Running a small example.

The example folder provides a simple example that you should be able to run immediately. The "core" pipeline which takes search engine and mgf input and produces fully rescored identifications using Prosit prediction on CPU. Execute this with:

```
inspire --pipeline core --config_file example/config.yml
```

This will run the rescoring and produce a report upon completion (this should take 2-3 minutes). This uses Mokapot rather than Percolator as it is easier to install together with inSPIRE. If you have Percolator installed and with to use it you can change the "rescoreMethod" in example/config.yml

## Plotting Spectra

To use the inSPIRE plotting utility you will need to further install plotly-orca:

```
conda install -c plotly plotly-orca
```

Then to to see example pair plots comparing the experimental data to prosit predictions call:

```
inspire --pipeline plotSpectra --config_file example/config.yml
```

This plots the PSMs specified in example/output/plotData.csv and saves the plots to example/output/spectralPlots.pdf. To generate these plots for a different PSM simply copy the first 4 columns of the relevant line from example/output/finalAssignments.csv into example/output/finalAssignments.csv and rerun the plotSpectra pipeline.

## inSPIRE-affinity

The core inSPIRE functionality can be executed via the "core" pipeline which will run rescoring using predicted spectra and provide final results to the user. If you wish to integrate binding affinity prediction you will have to make two modifications.

Firstly, you will need to add:

```
useBindingAffinity: asFeature
```

to your config.yml file.

Secondly, the execution will be slightly different. You will have to run two subsections of the "core" pipeline separately.

Firstly running the "prepare" pipeline will provide the input for all predictors. It will have created an mhcpan folder in the output folder with all unique peptides of each length saved as "inputLen{length}.txt". These can then be input to

NetMHCpan (see https://services.healthtech.dtu.dk/service.php?NetMHCpan-4.1). The prediction files should be placed in the mhcpan folder as "outputLen{length}preds.txt".

You can then run the "predictSpectra" pipeline, followed by the "rescore" pipeline which will use the predicted binding affinities together with the spectral predictions to produce rescored PSM assignments.

## Prosit Collision Energy Calibration

Prosit uses the collision energy setting of the mass spectrometer as input for MS2 prediction. While you could base this off the machine calibration, best results are expected if you callibrate prosit predictions for high scoring PSMs to the experimental data. Hence, for best performance, run the "calibrate" pipeline before executing the "core" pipeline. Execute this pipeline with:

```
inspire --config_file path-to-config --pipeline calibrate
```

The calibrate collision energy settting will be printed to the terminal and should be added to your config file as:

```
collisionEnergy: optimized-collision-energy
```

## Citation

### inSPIRE

inSPIRE is not yet published.

### Dependencies

Please also cite the relevant publications of the rescoring tools used, see details on https://github.com/percolator/percolator if using Percolator and details on https://github.com/wfondrie/mokapot if using Mokapot.

Please also cite the relevant publications of the spectral predictors, see details on https://www.proteomicsdb.org/prosit if using Prosit and details on https://github.com/compomics/ms2pip_c if using MS2PIP.

If using inSPIRE-affinity please also cite the relevant publications for NetMHCpan binding affinity predictions. See details on https://services.healthtech.dtu.dk/service.php?NetMHCpan-4.1.

## inSPIRE Config File

The configuration file is used to control inSPIRE execution using a set of keys and values specified in yaml format. yaml is a relatively simple format for entering many configs. The yaml file should take the form:

```
---
key1: value1
key2: value2
...
```

The keys needed are detailed below.

#### Required Configs

These are the minimal configs required to run inSPIRE.

| Key | Description |
|---|---|
| experimentTitle | A title for the experiment. |
| searchResults | The file path to the results of your ms search or a list of file paths if using multiple search results. |
| searchEngine | The search engine used (maxquant, mascot, or peaks). |
| outputFolder | Specify an output folder location which inSPIRE should write to. |
| scansFolder | Specify a folder containing the experimental spectra files in mgf or mzML format. |
| scansFormat | Specify the format of the spectra file (must be either mgf or mzML). |
| spectralPredictor | Either Prosit or MS2PIP |

#### Arguments Required for Prosit

| Key | Description |
|---|---|
| collisionEnergy | The mass spectrometer collision energy setting (run –calibrate pipeline to estimate the optimal setting). |
| deltaMethod | Recommended to set to "predictor" for immunopeptidome or "ignore" for tryptic proteome digestion. This defaults to ignore if MS2PIP is used. |

#### Arguments Required for MS2PIP Required

| Key | Description |
|---|---|
| ms2pipModel | Specify the MS2PIP model to be used (recommended HCD2021 for tryptic proteome data and Immuno-HCD for immunopeptidome). |

**Optional Settings (Experiment Specifications, Recommended to Check)**

The following settings are set by default but you should check that they are valid for your experimental set up.

| Key | Description |
|---|---|
| mzUnits | The units used for the m/z accuracy either Da for Daltons or ppm for Parts Per Million (default=Da). |
| mzAccuracy | The mz accuracy of the mass spectrometer in Daltons or ppm(default=0.02, default unit is Da). |
| rescoreMethod | inSPIRE supports either "mokapot" or "percolator" (default=mokapot). |
| nCores | The number of CPU cores you wish to use in rescoring (default=1). |
| fixedModifications | You must specify the fixed modifications used in a MaxQuant search. |
| forceReload | Boolean flag on whether to force models to be redownloaded in case you accidentally change the contents of your inSPIRE model folder. |

**Additional Options**

These setting are optional and are completely dependent on user preference.

| Key | Description |
|---|---|
| falseDiscoveryRate | This is the false discovery rate Percolator optimises for (default=0.01). |
| excludeFeatures | This specifies any features which you wish to exclude from rescoring (default=empty list). |
| includeFeatures | This specifies any features which you wish to include from rescoring and ignore all other features (default=empty list, meaning all features are used). |
| reduce | By default inSPIRE uses only the highest scoring hit per scan (and accession group if specified). If you set reduce to False this will consider all hits (default=True). |
| reuseInput | Boolean flag on whether to reuse formatted data after the first read in. When using Mascot in particular this may be useful as it reduces the time spend formatting data for input. |
| filterCysteine | Option to filter cysteins from rescoring if the sample contains unmodified cysteine and Prosit is being used. |

| Key | Description |
|---|---|
| dropUnknownPTMs | Whether to drop PSMs containing modifications other than oxidation of methionine and carbamidomethylation of cysteine. (default=True if Prosit used, False if ms2pip used) |

### Additional Configs for Mascot Distiller

If you have a combined mgf file from Mascot Distiller, you must add the following configs.

| Key | Description |
|---|---|
| scanTitleFormat | If Distiller used, set this argument to mascotDistiller. |
| distillerLog | If Distiller used, set this argument to the path to the "table_peptide_int.txt" file from Distiller. |
| combinedScansFile | Set this to the combined mgf file from Mascot Distiller and put the mgf file in folder specified by scansFolder. |

### NetMHCpan Configs

NetMHCpan predicts the binding affinity of a peptide for various HLA molecules. inSPIRE can use this as a validation of its predictions or as a feature for rescoring.

| Key | Description |
|---|---|
| useBindingAffinity | Set to asValidation if you want to check the percentage binders in your standard inSPIRE identifications. Set to asFeature if you want to use predicted binding affinity as a rescoring feature. |

### Ground Truth Datasets

Ground Truth Datasets may be useful in assessing the accuracy of your identification method. In this case, peptides with prior labels are measured via MS/MS. Some of these peptides are then inserted into the standard proteome and precision and recall your peptide identification method may be estimated.

To use ground truth datasets with inSPIRE, add the following parameters to the config file.

| Key | Description |
|---|---|
| groundTruth | The file path to your ground truth dataset matching scans to true peptide label. |
| groundTruthSeqKey | The name of the column containing the true peptide in the ground truth dataset. |
| groundTruthSourceKey | The name of the column containing the source file in the ground truth dataset. |
| groundTruthScanKey | The name of the column containing the scan number in the ground truth dataset. |
| groundTruthAccessionGroupKey* | The name of the column containing the accession group in the ground truth dataset. |

*Optional argument need only if the labelled peptides represent different accession groups.

## inSPIRE Pipelines.

This section details all possible pipeline you can run with inSPIRE. The 3 most important pipelines are calibrate, core, and plotSpectra.

### inspire –pipeline calibrate

As described above, this pipeline selects the highest scoring PSMs from the original search results and tests collision energy settings in the range 24 to 36 (inclusive) to find the optimal setting to be used. The calibrate collision energy is printed to the terminal.

### inspire –pipeline core

The core pipeline reads in and formats the search results file, predicts MS2 spectra for the peptides, and runs rescoring to provide final identifications of all peptides.

This pipeline can be executed in a number of sub-sections as detailed below.

### inspire –pipeline plotSpectra

This pipeline will plot experimental vs. Prosit predicted spectra for all PSMs specified in the plotData.csv file which should be placed in the output folder. This must at minimum specify the source file, scan number, peptide sequence, and modified sequence as provided in the inSPIRE finalAssignments.csv file.

### Subsections of the core pipeline

The “core” pipeline contains a large number of steps, which can be run individually. This can be important when using the binding affinity prediction (see

above) but also allows the user to make minor changes to the config file and rerun only the relevant sections of the pipeline.

**inspire –pipeline prepare**

This is the first step of the “core” functionality. It reads in the search results and formats input for Prosit/MS2PIP predictions and NetMHCpan if required.

**inspire –pipeline predictSpectra**

This predicts MS2 spectra using the specified spectral predictor and writes to msp format.

**inspire –pipeline rescore**

This option executes all of the remaining steps of the pipeline.

**inspire –pipeline featureGeneration**

This pipeline generate

**inspire –pipeline featureSelection+**

This pipeline filters the feature set as required by the config file (default does not apply any filter), runs rescoring, formats the output, and generates a html report with details of performance and comparison to a baseline rescoring without spectral prediction.
