# Supplementary material for: inSPIRE: An Open-Source Tool for Increased Mass Spectrometry Identification Rates Using Prosit Spectral Prediction
Source: Mol Cell Proteomics. 2022 Oct 21;21(12):100432. doi: 10.1016/j.mcpro.2022.100432 (PMC9720494; doi:10.1016/j.mcpro.2022.100432)

File S3. inSPIRE Reports for all datasets from which main text figures are generated. This file contains all of the inSPIRE html reports for the results which are reported in the Figures of the main text, converted to pdf and merged to a single file. These reports provide details of the relative feature importance, distribution of key features, and improvement over the baseline Percolator implementation.

inSPIRE Report for IP A02 Expressed inSPIRE Standard

inSPIRE Settings Used

inSPIRE Settings for Experiment IP A02 Expressed inSPIRE Standard:

Config	Setting
searchEngine	maxquant
scansFormat	mgf
spectralPredictor	prosit
deltaMethod	predictor
rescoreMethod	percolator
searchResults	section_1_data/maxQuant/A02_expressed/msms.txt
scansFolder	section_1_data/scans
outputFolder	spire_1_A02_expressed/output
collisionEnergy	33
mzAccuracy	0.02
mzUnits	Da
fixedModifications	None
forceReload	False
falseDiscoveryRate	0.01
excludeFeatures	[]
includeFeatures	None
reduce	False
filterCysteine	False
dropUnknownPTMs	True
useBindingAffinity	asValidation

Selected Features and Importance

The table below shows the importance of the final feature set used by percolator. Strong positive values (highlighted in green) may indicate that higher feature values are more common among target PSMs while strongly negative values (highlighted in red) may indicate that higher feature values are more common among decoy PSMs. However, it is also possible that a feature like searchEngineScore ends up with a negative coefficient simply because it is so strongly correlated to a more powerful feature like deltaScore which has a strong positive coefficent.

Weights of Features Selected by inSPIRE.

feature	weightFold1	weightFold2	weightFold3	averageWeight
spearmanR	1.3452	1.4846	1.6053	1.478
sequenceLength	1.1829	1.4514	1.1963	1.277
deltaScore	0.795	0.8879	0.7286	0.804
spearmanMajorIons	1.0657	0.7756	0.3491	0.73
nMajorMatchedDivFrags	0.7795	0.5101	0.6836	0.658
spectralAngle	0.279	0.642	0.488	0.47
matchedCoverage	0.5489	0.3993	0.1437	0.364
nDeltasAboveThreshold	0.3598	0.5584	-0.0212	0.299
nMinorMatchedDivFrags	0.2751	0.2815	0.2927	0.283
avgResidueMass	0.2789	0.1739	0.2302	0.228
maxTypeSpectralAngle	-0.1037	0.4367	0.3115	0.215
medianFragmentMzError	0.1262	0.251	0.1895	0.189
yIsDominantIonSeries	0.16	0.0939	0.2453	0.166
nDeltasAboveZero	0.1965	-0.3464	0.6076	0.153
fracUnique	0.1885	0.1068	0.1353	0.144
prositDeltaQuartile1	0.0539	0.1756	0.1331	0.121
nVarMods	0.1783	0.0887	0.0443	0.104
minMatchedCoverage	-0.014	0.1928	-0.0263	0.051
fracMatchedKR	0.1973	-0.0676	0.005	0.045
nRepeatedResidues	0.1336	-0.0987	0.0895	0.041
predNotFoundCoverage	-0.0846	0.091	0.0589	0.022
fromChimera	0.0421	0.0383	-0.1394	-0.02
charge	-0.1653	-0.201	0.2394	-0.042
nMinorNotMatchableDivFrags	0.2586	-0.2714	-0.1155	-0.043
fragmentMzErrorVariance	0.2415	-0.2827	-0.1306	-0.057
nLossIonsDivFrags	-0.1791	-0.1827	0.1753	-0.062
maxMatchedCoverage	-0.1373	0.1224	-0.2007	-0.072
nMajorNotMatchableDivFrags	-0.1758	-0.0949	-0.0254	-0.099
spectrumDensity	-0.5252	0.2179	-0.0976	-0.135
medianAbsoluteError	0.0606	-0.1832	-0.2981	-0.14
fracKR	-0.3305	-0.197	-0.2973	-0.275
minPrositDelta	-0.3304	-0.4612	-0.356	-0.383
maxPrositDelta	-1.0864	-0.0809	-0.1015	-0.423
prositDeltaQuartile3	-0.2231	-0.4579	-0.6579	-0.446
prositDeltaMedian	-0.723	-0.6385	-0.1775	-0.513
engineScore	-0.7148	-0.8539	-0.5464	-0.705
pearsonR	-0.3456	-1.3398	-0.4496	-0.712
seqLenMeanDiff	-0.8063	-0.7009	-1.2932	-0.933
deltaRT	-1.4215	-1.4949	-1.3593	-1.425
m0	-3.7942	-3.3536	-2.9707	-3.373

Feature Distributions

These Violin Plots show the distributions of the three most heavily positive and heavily negative weighted features for accepted and rejected PSMs.

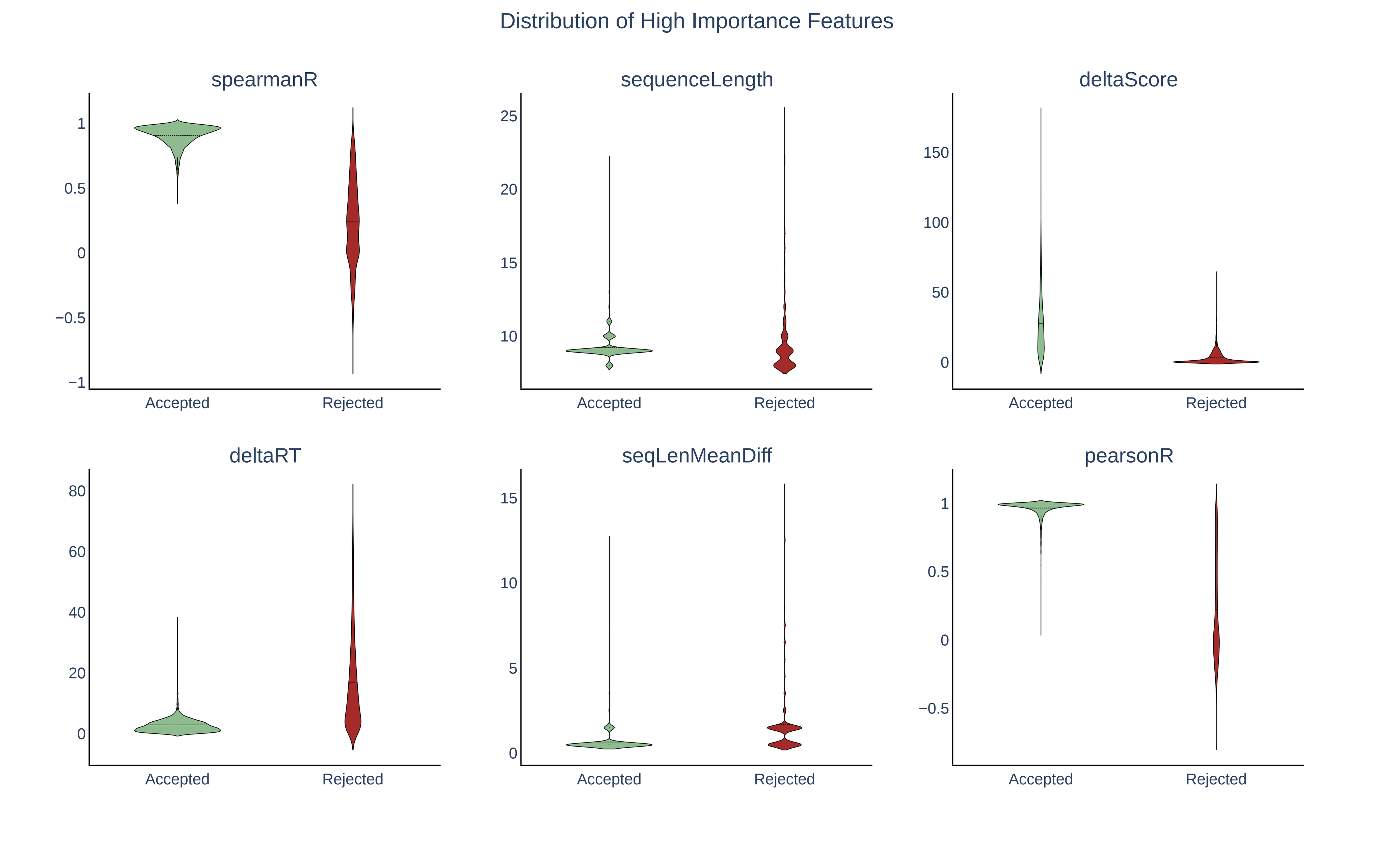

inSPIRE Performance: Number of PSMs Identified

This shows the number of PSMs discovered by inSPIRE compared to the original search engine for q-value cut offs between 0.01 and 0.1.

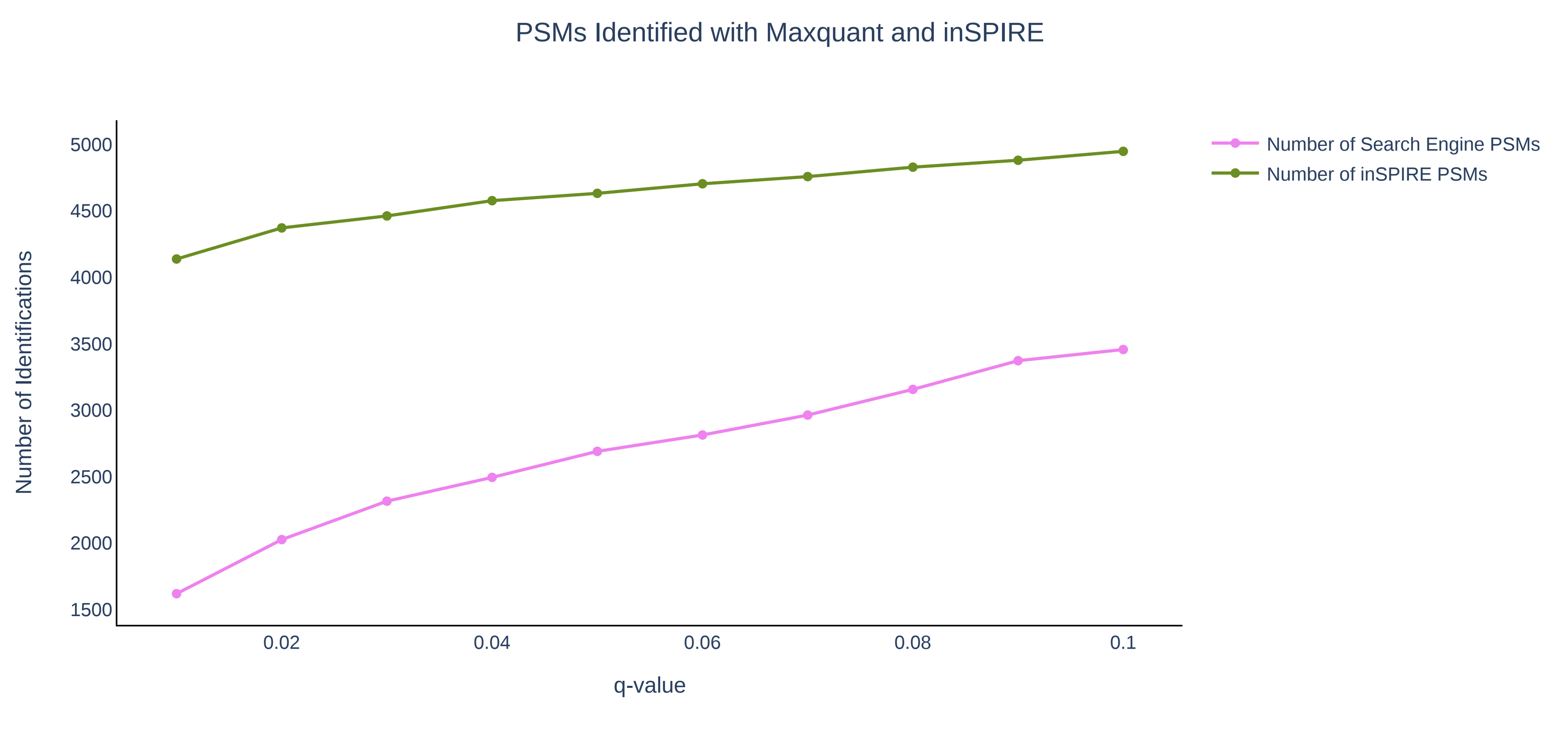

inSPIRE Performance: Percentage Binders Identified

This shows the percentage of HLA-I binders as predicted by NetMHCpan among the PSMs identified by inSPIRE compared to the original search engine results. Note that this plot is more meaningful if you have set useBindingAffinty to asValidation, as if it is set to asFeature it is unsurprising that inSPIRE would produce a higher percentage of HLA binders.

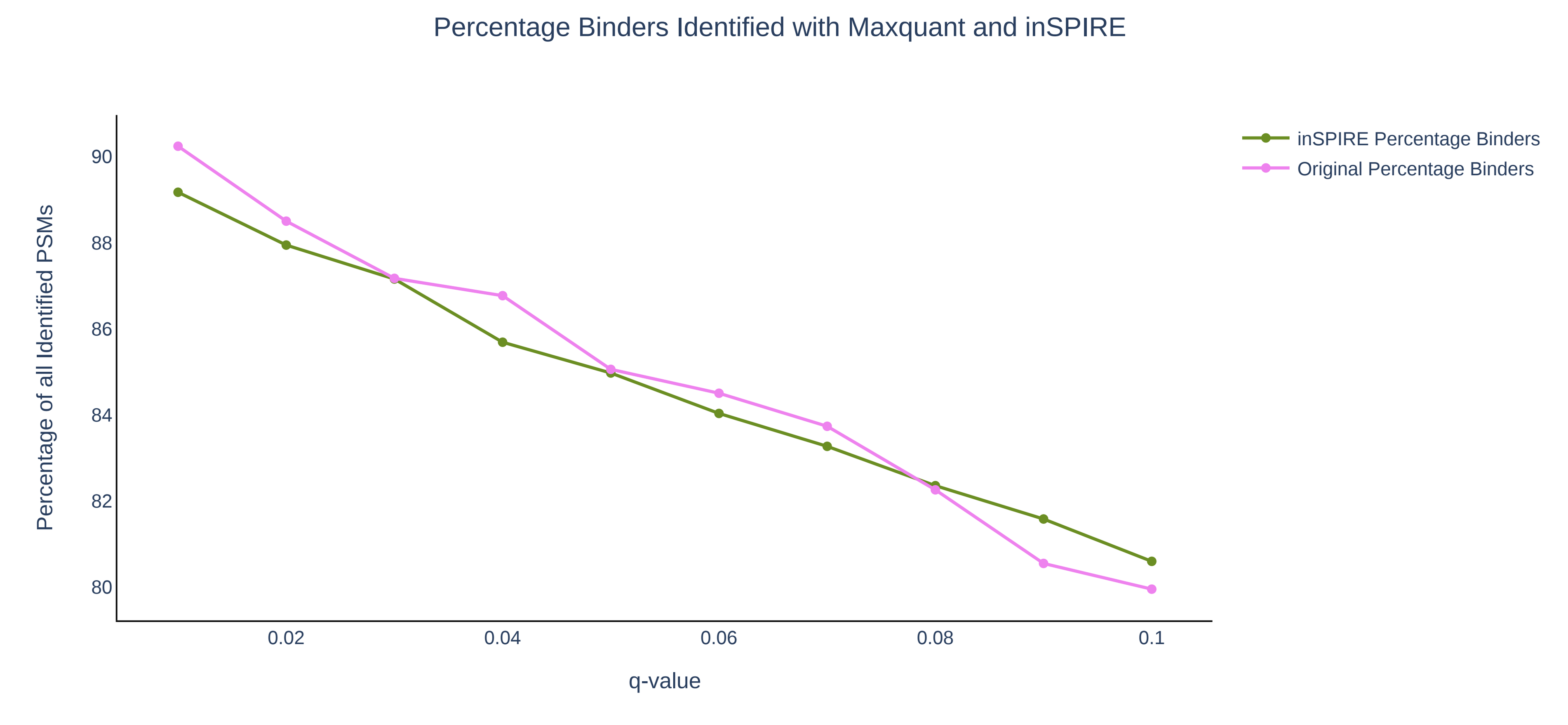

inSPIRE Report for IP A02 Gencode inSPIRE-Standard

inSPIRE Settings Used

inSPIRE Settings for Experiment IP A02 Gencode inSPIRE-Standard:

Config	Setting
searchEngine	maxquant
scansFormat	mgf
spectralPredictor	prosit
deltaMethod	predictor
rescoreMethod	percolator
searchResults	section_1_data/maxQuant/A02_gencode/msms.txt
scansFolder	section_1_data/scans
outputFolder	spire_1_A02_gencode/output
collisionEnergy	33
mzAccuracy	0.02
mzUnits	Da
fixedModifications	None
forceReload	False
falseDiscoveryRate	0.01
excludeFeatures	[]
includeFeatures	None
reduce	False
filterCysteine	False
dropUnknownPTMs	True
useBindingAffinity	asValidation

Selected Features and Importance

The table below shows the importance of the final feature set used by percolator. Strong positive values (highlighted in green) may indicate that higher feature values are more common among target PSMs while strongly negative values (highlighted in red) may indicate that higher feature values are more common among decoy PSMs. However, it is also possible that a feature like searchEngineScore ends up with a negative coefficient simply because it is so strongly correlated to a more powerful feature like deltaScore which has a strong positive coefficent.

Weights of Features Selected by inSPIRE.

feature	weightFold1	weightFold2	weightFold3	averageWeight
spearmanR	1.2685	1.0535	1.6082	1.31
sequenceLength	1.4487	1.0371	1.377	1.288
spearmanMajorIons	0.6642	0.5929	1.0588	0.772
nMajorMatchedDivFrags	0.6958	0.9131	0.5265	0.712
deltaScore	0.6177	0.8345	0.6823	0.712
matchedCoverage	0.8626	0.413	0.3915	0.556
maxTypeSpectralAngle	0.4642	0.3908	0.7843	0.546
nDeltasAboveZero	0.4896	0.5944	0.4033	0.496
spectralAngle	1.12	0.285	0.077	0.494
nMinorMatchedDivFrags	0.4219	0.4575	0.3205	0.4
prositDeltaQuartile1	0.385	0.2178	0.4867	0.363
charge	0.1354	0.2972	0.4096	0.281
avgResidueMass	0.3363	0.1373	0.3319	0.269
nVarMods	0.0954	0.203	0.2714	0.19
yIsDominantIonSeries	0.1901	0.122	0.1597	0.157
medianFragmentMzError	0.361	0.0192	0.0815	0.154
nRepeatedResidues	0.109	0.1802	0.1504	0.147
spectrumDensity	-0.2711	0.2143	0.4861	0.143
fracMatchedKR	-0.0828	0.0567	0.3858	0.12
nDeltasAboveThreshold	0.2149	0.0817	-0.0307	0.089
fracUnique	0.1501	0.0422	0.0067	0.066
nMajorNotMatchableDivFrags	-0.0086	-0.0139	0.149	0.042
fragmentMzErrorVariance	-0.1937	0.1732	-0.013	-0.011
predNotFoundCoverage	-0.0466	0.0398	-0.0341	-0.014
nLossIonsDivFrags	-0.088	0.0407	-0.0581	-0.035
minMatchedCoverage	-0.1243	-0.1951	0.1411	-0.059
fromChimera	-0.0933	-0.084	-0.1419	-0.106
medianAbsoluteError	-0.1161	-0.5711	-0.2702	-0.319
maxMatchedCoverage	-0.334	-0.4047	-0.2964	-0.345
prositDeltaQuartile3	-0.7527	-0.5447	0.1818	-0.372
nMinorNotMatchableDivFrags	0.064	-0.4797	-0.7641	-0.393
fracKR	-0.4437	-0.2385	-0.6844	-0.456
maxPrositDelta	-0.2624	-0.3425	-0.8532	-0.486
engineScore	-0.6489	-0.4271	-0.5514	-0.542
prositDeltaMedian	-0.4624	-0.3883	-0.8738	-0.575
minPrositDelta	-0.5698	-0.5098	-0.713	-0.598
seqLenMeanDiff	-0.9405	-0.978	-1.1449	-1.021
pearsonR	-1.6237	-0.879	-0.9892	-1.164
deltaRT	-1.5495	-1.1514	-1.3059	-1.336
m0	-3.1898	-2.5812	-3.8387	-3.203

Feature Distributions

These Violin Plots show the distributions of the three most heavily positive and heavily negative weighted features for accepted and rejected PSMs.

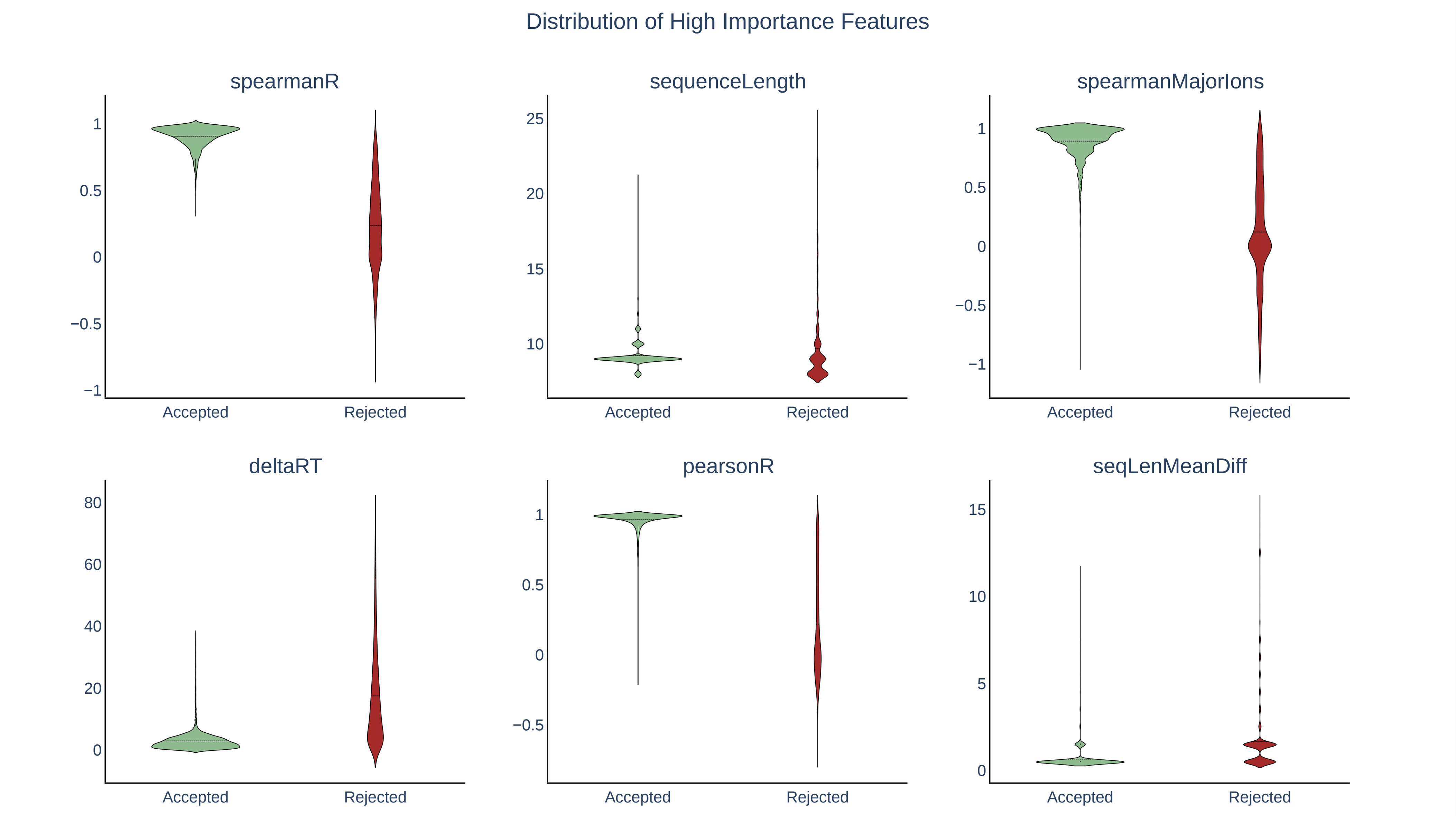

inSPIRE Performance: Number of PSMs Identified

This shows the number of PSMs discovered by inSPIRE compared to the original search engine for q-value cut offs between 0.01 and 0.1.

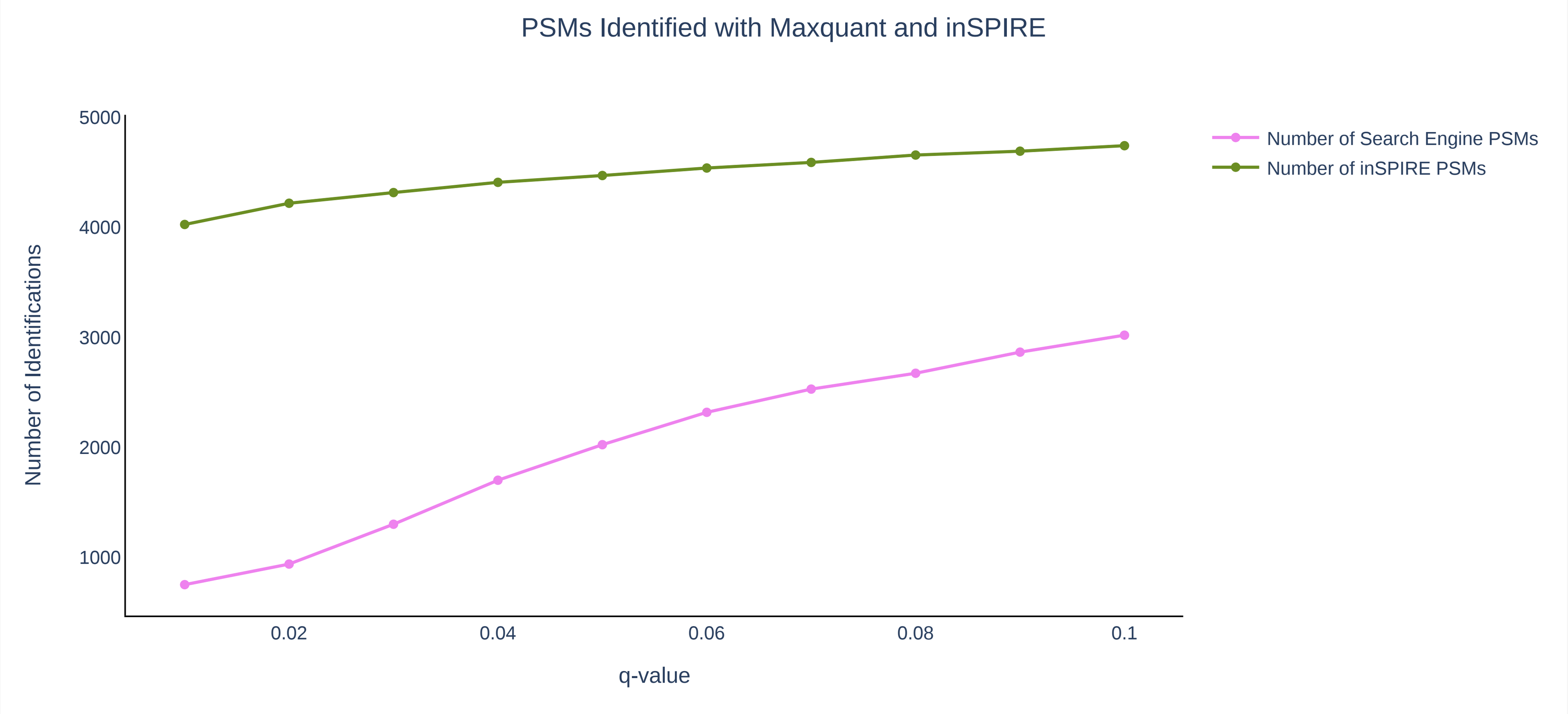

inSPIRE Performance: Percentage Binders Identified

This shows the percentage of HLA-I binders as predicted by NetMHCpan among the PSMs identified by inSPIRE compared to the original search engine results. Note that this plot is more meaningful if you have set useBindingAffinity to asValidation, as if it is set to asFeature it is unsurprising that inSPIRE would produce a higher percentage of HLA binders.

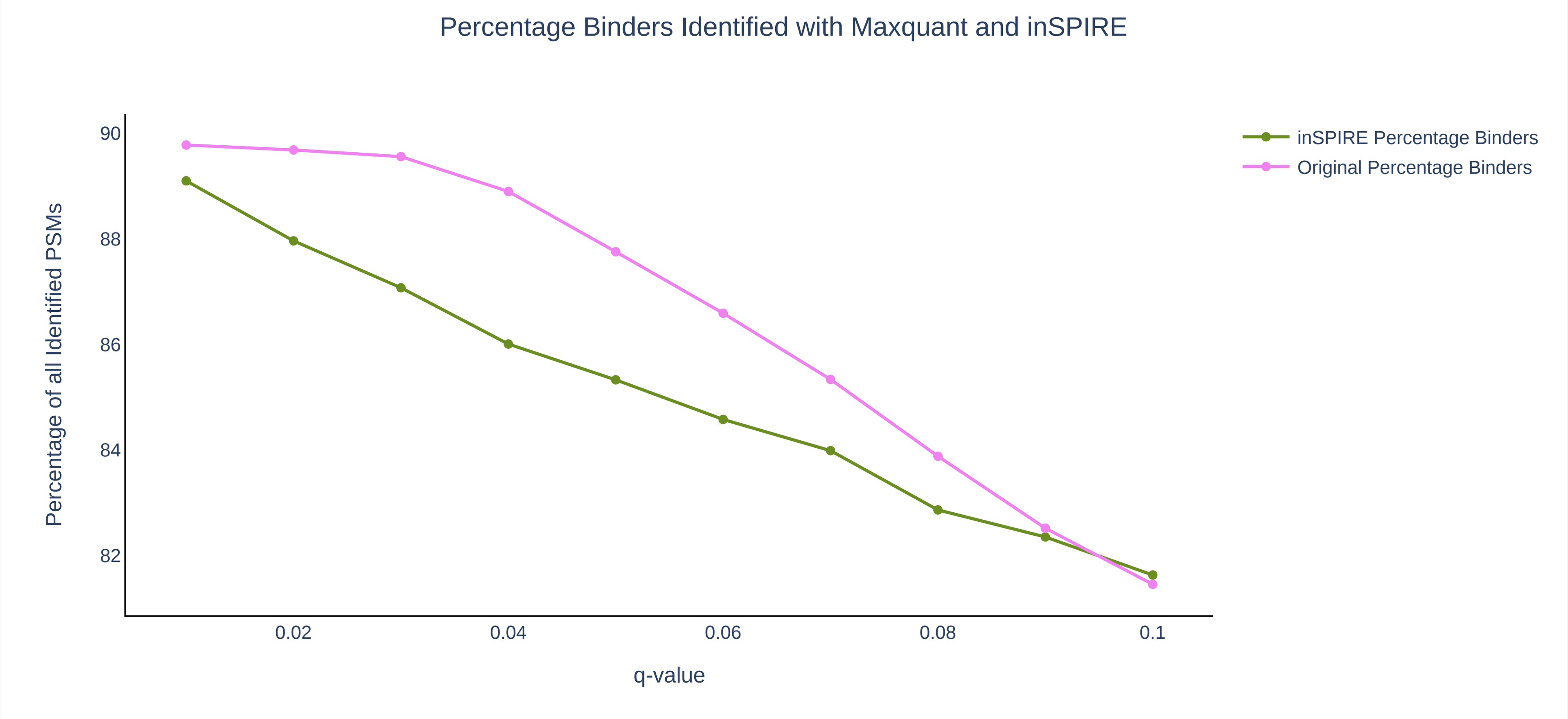

inSPIRE Report for IP B07 Expressed

inSPIRE Settings Used

inSPIRE Settings for Experiment IP B07 Expressed:

Config	Setting
searchEngine	maxquant
scansFormat	mgf
spectralPredictor	prosit
deltaMethod	predictor
rescoreMethod	percolator
searchResults	section_1_data/maxQuant/ip_expressed/msms.txt
scansFolder	section_1_data/scans
outputFolder	spire_1_ip_expressed/output
collisionEnergy	33
mzAccuracy	0.02
mzUnits	Da
fixedModifications	None
forceReload	False
falseDiscoveryRate	0.01
excludeFeatures	[]
includeFeatures	None
reduce	False
filterCysteine	False
dropUnknownPTMs	True
useBindingAffinity	asValidation

Selected Features and Importance

The table below shows the importance of the final feature set used by percolator. Strong positive values (highlighted in green) may indicate that higher feature values are more common among target PSMs while strongly negative values (highlighted in red) may indicate that higher feature values are more common among decoy PSMs. However, it is also possible that a feature like searchEngineScore ends up with a negative coefficient simply because it is so strongly correlated to a more powerful feature like deltaScore which has a strong positive coefficent.

Weights of Features Selected by inSPIRE.

feature	weightFold1	weightFold2	weightFold3	averageWeight
sequenceLength	1.3957	3.71	2.4141	2.507
spectralAngle	0.656	2.5772	1.4106	1.548
matchedCoverage	1.0262	1.7692	0.8418	1.212
spearmanR	0.4803	1.8497	1.0382	1.123
deltaScore	0.6832	1.1927	1.0244	0.967
spearmanMajorIons	0.3019	1.0142	0.3339	0.55
avgResidueMass	0.2107	0.8241	0.5135	0.516
maxTypeSpectralAngle	0.3505	0.8881	0.2621	0.5
minMatchedCoverage	0.325	0.2768	0.3305	0.311
medianAbsoluteError	-0.2346	0.8204	0.3388	0.308
maxMatchedCoverage	0.3129	0.2433	0.2469	0.268
predNotFoundCoverage	0.1252	0.3362	0.1541	0.205
nMinorMatchedDivFrags	-0.0772	0.2717	0.3345	0.176
nDeltasAboveZero	0.1007	0.1551	0.0936	0.116
fracUnique	0.0854	0.0357	0.2116	0.111
nMajorMatchedDivFrags	-0.0271	0.0578	0.2535	0.095
prositDeltaMedian	0.03	0.0019	0.2281	0.087
maxPrositDelta	0.1155	-0.1357	0.2691	0.083
fromChimera	-0.0153	0.027	0.1485	0.053
yIsDominantIonSeries	0.1223	0.0385	-0.0144	0.049
nMajorNotMatchableDivFrags	-0.0114	0.0369	0.0099	0.012
prositDeltaQuartile3	-0.269	0.3744	-0.1362	-0.01
spectrumDensity	-0.0948	-0.0704	0.0401	-0.042
nVarMods	-0.0396	-0.1047	-0.0094	-0.051
fracMatchedKR	-0.0268	0.0711	-0.2745	-0.077
minPrositDelta	0.0276	-0.295	-0.0068	-0.091
nLossIonsDivFrags	0.0235	-0.2145	-0.1606	-0.117
nRepeatedResidues	-0.0637	-0.2325	-0.1063	-0.134
nMinorNotMatchableDivFrags	-0.2154	0.0826	-0.3282	-0.154
charge	-0.0869	-0.4218	-0.2711	-0.26
nDeltasAboveThreshold	-0.06	-0.3872	-0.3862	-0.278
fracKR	-0.1884	-0.5346	-0.1117	-0.278
prositDeltaQuartile1	-0.0625	-0.7105	-0.4841	-0.419
medianFragmentMzError	-0.2467	-0.628	-0.474	-0.45
fragmentMzErrorVariance	0.1654	-1.202	-0.5082	-0.515
seqLenMeanDiff	-0.5398	-1.0076	-0.814	-0.787
engineScore	-0.714	-1.353	-0.849	-0.972
pearsonR	-0.4302	-2.895	-0.8341	-1.386
deltaRT	-1.1369	-5.5384	-2.4017	-3.026
m0	-2.3353	-7.378	-3.7948	-4.503

Feature Distributions

These Violin Plots show the distributions of the three most heavily positive and heavily negative weighted features for accepted and rejected PSMs.

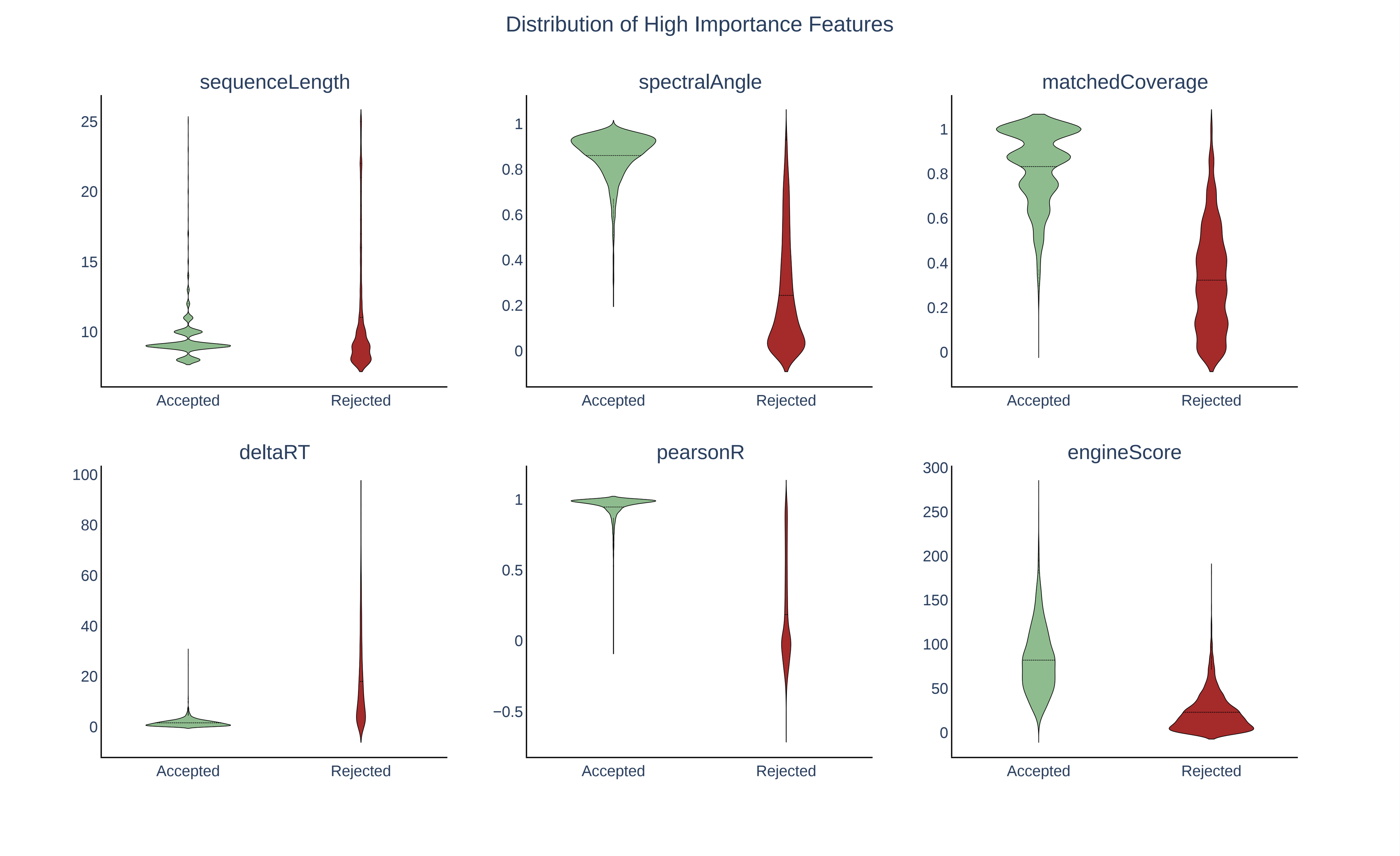

inSPIRE Performance: Number of PSMs Identified

This shows the number of PSMs discovered by inSPIRE compared to the original search engine for q-value cut offs between 0.01 and 0.1.

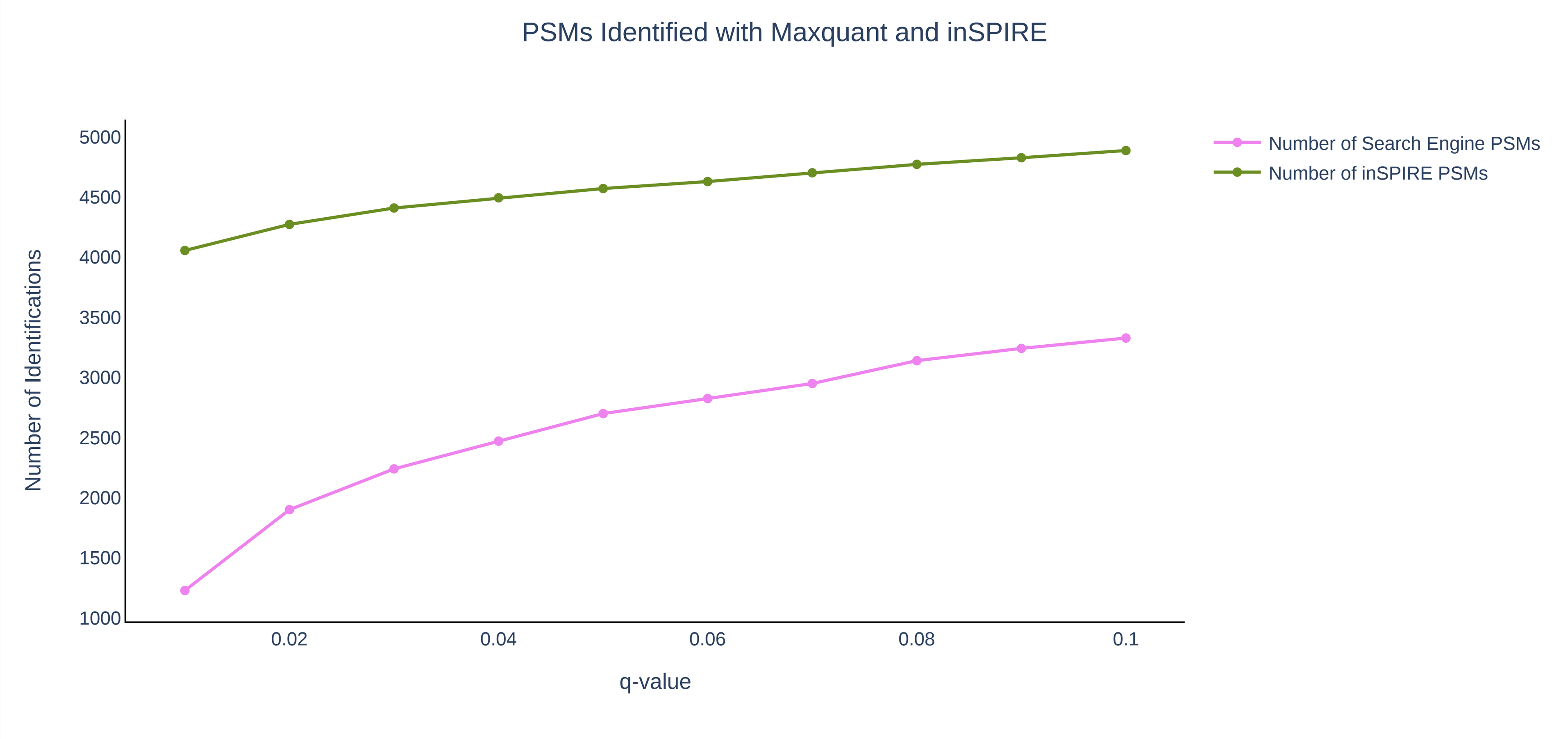

inSPIRE Performance: Percentage Binders Identified

This shows the percentage of HLA-I binders as predicted by NetMHCpan among the PSMs identified by inSPIRE compared to the original search engine results. Note that this plot is more meaningful if you have set useBindingAffinity to asValidation, as if it is set to asFeature it is unsurprising that inSPIRE would produce a higher percentage of HLA binders.

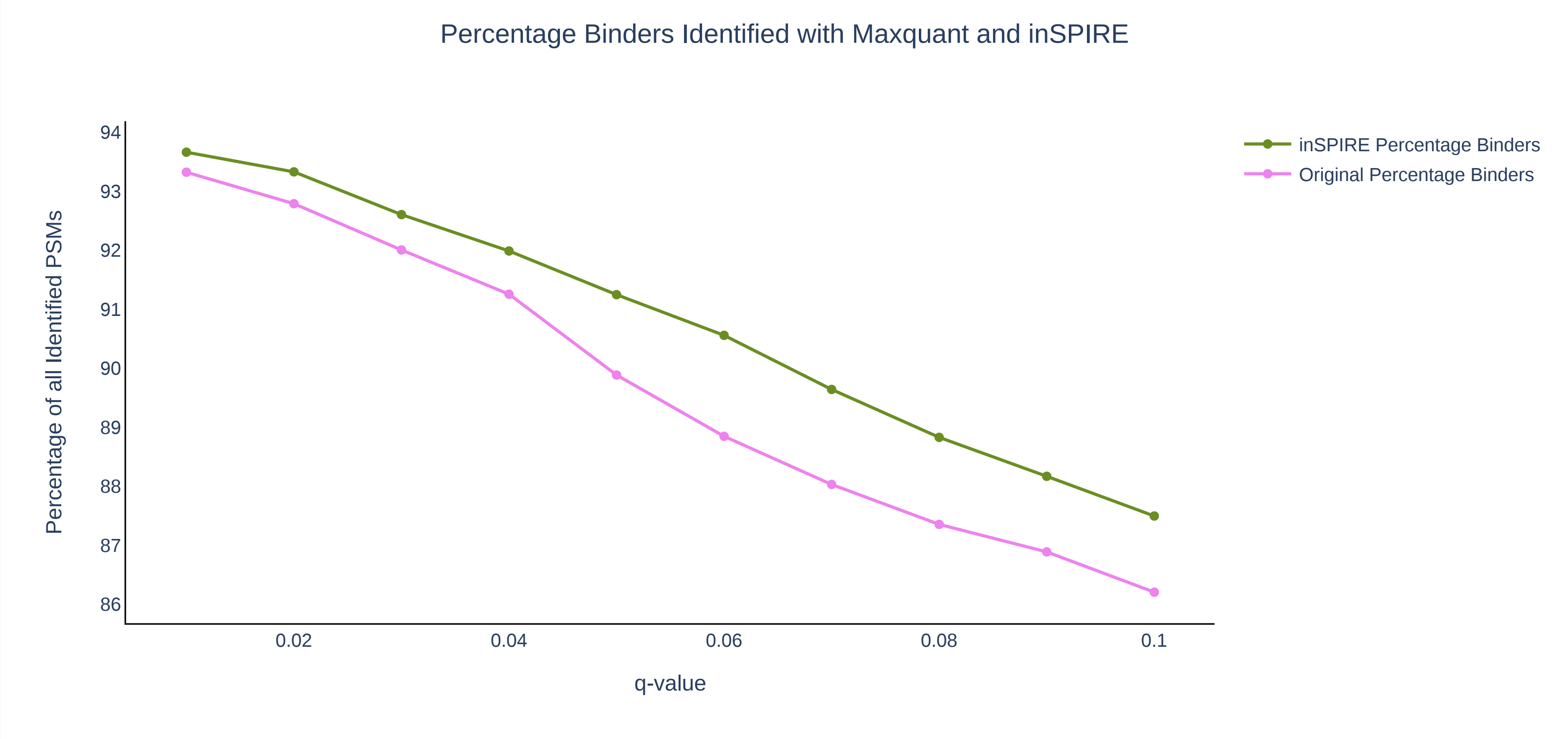

inSPIRE Report for IP B07 Gencode

inSPIRE Settings Used

inSPIRE Settings for Experiment IP B07 Gencode:

Config	Setting
searchEngine	maxquant
scansFormat	mgf
spectralPredictor	prosit
deltaMethod	predictor
rescoreMethod	percolator
searchResults	section_1_data/maxQuant/ip_gencode/msms.txt
scansFolder	section_1_data/scans
outputFolder	spire_1_ip_gencode/output
collisionEnergy	33
mzAccuracy	0.02
mzUnits	Da
fixedModifications	None
forceReload	False
falseDiscoveryRate	0.01
excludeFeatures	[]
includeFeatures	None
reduce	False
filterCysteine	False
dropUnknownPTMs	True
useBindingAffinity	asValidation

Selected Features and Importance

The table below shows the importance of the final feature set used by percolator. Strong positive values (highlighted in green) may indicate that higher feature values are more common among target PSMs while strongly negative values (highlighted in red) may indicate that higher feature values are more common among decoy PSMs. However, it is also possible that a feature like searchEngineScore ends up with a negative coefficient simply because it is so strongly correlated to a more powerful feature like deltaScore which has a strong positive coefficent.

Weights of Features Selected by inSPIRE.

feature	weightFold1	weightFold2	weightFold3	averageWeight
sequenceLength	2.7641	2.4184	2.0654	2.416
spectralAngle	1.0934	1.2141	1.2459	1.184
matchedCoverage	1.4711	1.0848	0.9095	1.155
spearmanR	0.6443	1.296	0.9057	0.949
deltaScore	0.6496	1.0336	0.516	0.733
maxTypeSpectralAngle	0.6951	0.5975	0.3665	0.553
avgResidueMass	0.5102	0.3399	0.4764	0.442
spearmanMajorIons	0.5078	0.3318	0.1816	0.34
nMinorMatchedDivFrags	-0.1858	0.5283	0.3567	0.233
medianAbsoluteError	0.1159	0.5254	0.0392	0.227
prositDeltaMedian	0.2445	0.0647	0.316	0.208
fracUnique	0.2068	0.2362	0.1688	0.204
predNotFoundCoverage	0.0125	0.2365	0.2902	0.18
maxPrositDelta	-0.0106	0.2425	0.2436	0.159
minMatchedCoverage	0.4871	-0.1726	0.1545	0.156
nMajorMatchedDivFrags	0.1372	0.1357	0.1117	0.128
nDeltasAboveZero	0.1714	0.1918	-0.0281	0.112
nMajorNotMatchableDivFrags	0.1455	0.0177	0.0779	0.08
nVarMods	0.0555	-0.0645	0.216	0.069
maxMatchedCoverage	0.1887	-0.1128	0.0986	0.058
nMinorNotMatchableDivFrags	0.0289	0.3928	-0.2792	0.047
fromChimera	-0.144	-0.0161	0.0308	-0.043
fracKR	0.0691	-0.0227	-0.2047	-0.053
yIsDominantIonSeries	-0.0805	0.0094	-0.1473	-0.073
nLossIonsDivFrags	0.0057	-0.0937	-0.1402	-0.076
nRepeatedResidues	-0.125	-0.0571	-0.2987	-0.16
fracMatchedKR	-0.2723	-0.1848	-0.0871	-0.181
charge	-0.1779	-0.1568	-0.25	-0.195
minPrositDelta	-0.2336	-0.2401	-0.1238	-0.199
prositDeltaQuartile3	0.0576	-0.0197	-0.7505	-0.238
spectrumDensity	-0.2879	-0.6609	0.1925	-0.252
nDeltasAboveThreshold	-0.4673	-0.5163	0.1946	-0.263
prositDeltaQuartile1	-0.3766	-0.2606	-0.254	-0.297
fragmentMzErrorVariance	-0.2228	-0.732	-0.4502	-0.468
medianFragmentMzError	-0.7699	-0.8884	-0.0264	-0.562
engineScore	-0.801	-0.552	-0.575	-0.643
seqLenMeanDiff	-0.9066	-0.4749	-0.8586	-0.747
pearsonR	-1.0373	-1.3052	-0.9867	-1.11
deltaRT	-2.8444	-2.5769	-0.7233	-2.048
m0	-4.7813	-4.0085	-2.8398	-3.877

Feature Distributions

These Violin Plots show the distributions of the three most heavily positive and heavily negative weighted features for accepted and rejected PSMs.

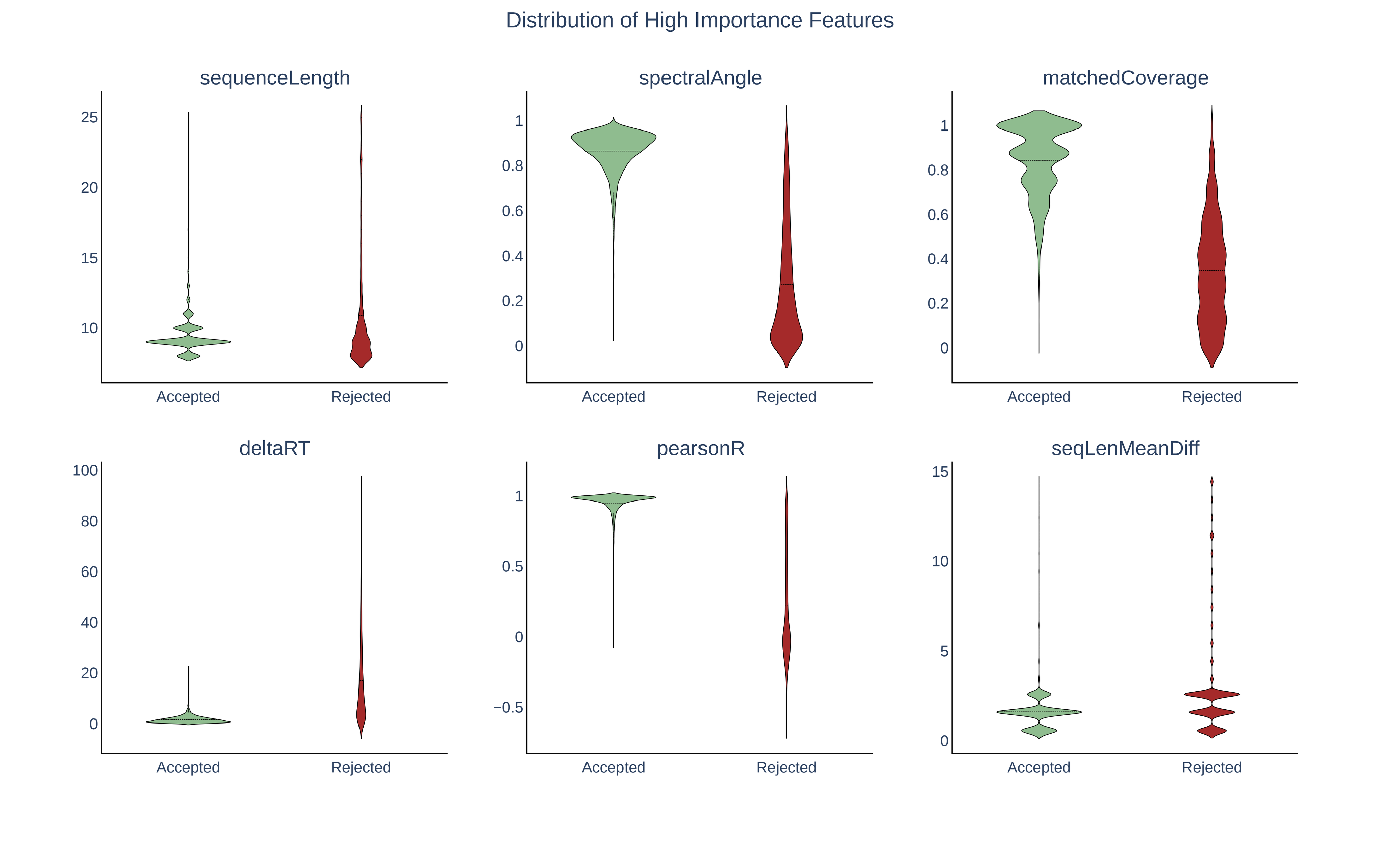

inSPIRE Performance: Number of PSMs Identified

This shows the number of PSMs discovered by inSPIRE compared to the original search engine for q-value cut offs between 0.01 and 0.1.

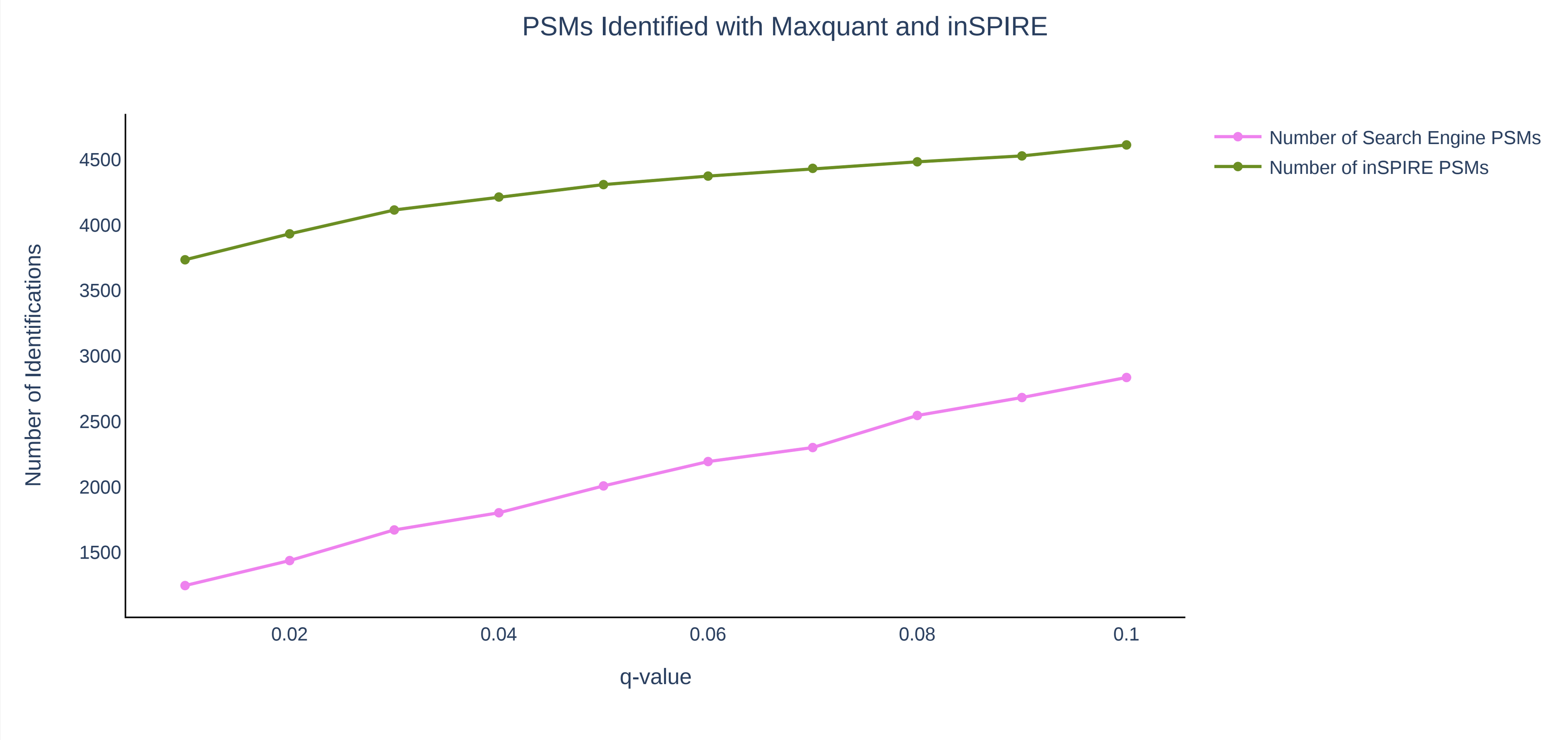

inSPIRE Performance: Percentage Binders Identified

This shows the percentage of HLA-I binders as predicted by NetMHCpan among the PSMs identified by inSPIRE compared to the original search engine results. Note that this plot is more meaningful if you have set useBindingAffinty to asValidation, as if it is set to asFeature it is unsurprising that inSPIRE would produce a higher percentage of HLA binders.

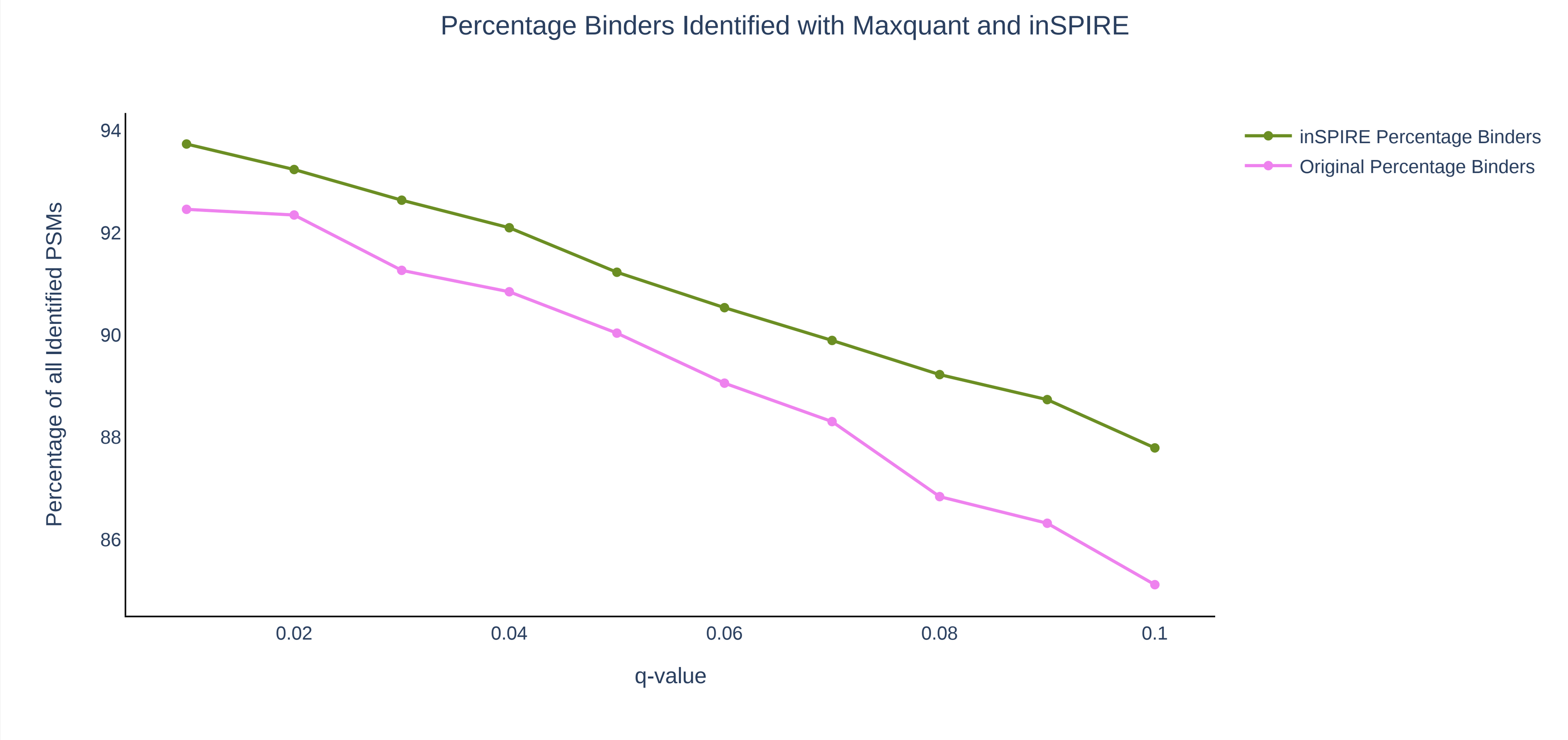

inSPIRE Report for Tryptic K562 Expressed

inSPIRE Settings Used

inSPIRE Settings for Experiment Tryptic K562 Expressed:

Config	Setting
searchEngine	maxquant
scansFormat	mgf
spectralPredictor	prosit
deltaMethod	predictor
rescoreMethod	percolator
searchResults	section_1_data/maxQuant/tryptic_expressed/txt/msms.txt
scansFolder	section_1_data/scans
outputFolder	spire_1_tryptic_expressed/output
collisionEnergy	29
mzAccuracy	0.02
mzUnits	Da
fixedModifications	['Carbamidomethylation']
forceReload	False
falseDiscoveryRate	0.01
excludeFeatures	[]
includeFeatures	None
reduce	False
filterCysteine	False
dropUnknownPTMs	True
useBindingAffinity	None

Selected Features and Importance

The table below shows the importance of the final feature set used by percolator. Strong positive values (highlighted in green) may indicate that higher feature values are more common among target PSMs while strongly negative values (highlighted in red) may indicate that higher feature values are more common among decoy PSMs. However, it is also possible that a feature like searchEngineScore ends up with a negative coefficient simply because it is so strongly correlated to a more powerful feature like deltaScore which has a strong positive coefficient.

Weights of Features Selected by inSPIRE.

feature	weightFold1	weightFold2	weightFold3	averageWeight
sequenceLength	1.5229	1.1318	1.3646	1.34
spearmanR	1.2774	0.9733	1.0991	1.117
deltaScore	1.0697	0.7296	0.9291	0.909
spectralAngle	0.7433	0.4439	1.2364	0.808
nMajorMatchedDivFrags	0.7311	0.3606	0.657	0.583
maxMatchedCoverage	0.5872	0.6545	0.4132	0.552
nMinorMatchedDivFrags	0.5821	0.2785	0.6256	0.495
spearmanMajorIons	0.3498	0.213	0.3038	0.289
maxPrositDelta	0.2483	0.2634	0.2696	0.26
matchedCoverage	0.142	0.288	0.2684	0.233
avgResidueMass	0.2098	0.1644	0.2131	0.196
fracUnique	0.2255	0.1622	0.1504	0.179
nDeltasAboveThreshold	0.27	0.0695	0.0985	0.146
prositDeltaMedian	-0.0203	0.1875	0.2454	0.138
nRepeatedResidues	0.2028	0.0749	0.0703	0.116
nVarMods	0.005	0.1438	0.0832	0.077
maxTypeSpectralAngle	0.0566	0.0342	0.1139	0.068
minMatchedCoverage	0.1084	0.0414	-0.0125	0.046
nDeltasAboveZero	0.0972	0.032	-0.0188	0.037
medianAbsoluteError	0.1237	-0.0443	-0.035	0.015
yIsDominantIonSeries	0.0319	-0.0388	0.0442	0.012
nMajorNotMatchableDivFrags	-0.008	-0.0545	-0.0057	-0.023
predNotFoundCoverage	-0.0489	0.0237	-0.054	-0.026
nMinorNotMatchableDivFrags	-0.1606	-0.0189	0.0184	-0.054
fracKR	-0.0302	-0.0309	-0.1033	-0.055
nLossIonsDivFrags	-0.0244	-0.0791	-0.0803	-0.061
fromChimera	-0.1073	-0.0582	-0.0306	-0.065
fracC	-0.1059	-0.0908	-0.0293	-0.075
missedCleavages	-0.1063	-0.1296	-0.0126	-0.083
charge	-0.0818	-0.1047	-0.0703	-0.086
prositDeltaQuartile3	-0.1237	-0.1626	-0.0779	-0.121
minPrositDelta	-0.1965	-0.0173	-0.153	-0.122
fracMatchedKR	-0.13	-0.0933	-0.1657	-0.13
medianFragmentMzError	-0.2266	-0.055	-0.2421	-0.175
prositDeltaQuartile1	-0.3083	-0.3893	-0.2992	-0.332
spectrumDensity	-0.3347	-0.3937	-0.4657	-0.398
seqLenMeanDiff	-0.7155	-0.5918	-0.6056	-0.638
pearsonR	-0.6847	-0.31	-1.0334	-0.676
fragmentMzErrorVariance	-0.4589	-0.1865	-2.1112	-0.919
engineScore	-1.32	-0.944	-1.28	-1.181
deltaRT	-1.0959	-1.5113	-1.5904	-1.399
m0	0.5448	0.1335	-0.0964	0.194

Feature Distributions

These Violin Plots show the distributions of the three most heavily positive and heavily negative weighted features for accepted and rejected PSMs.

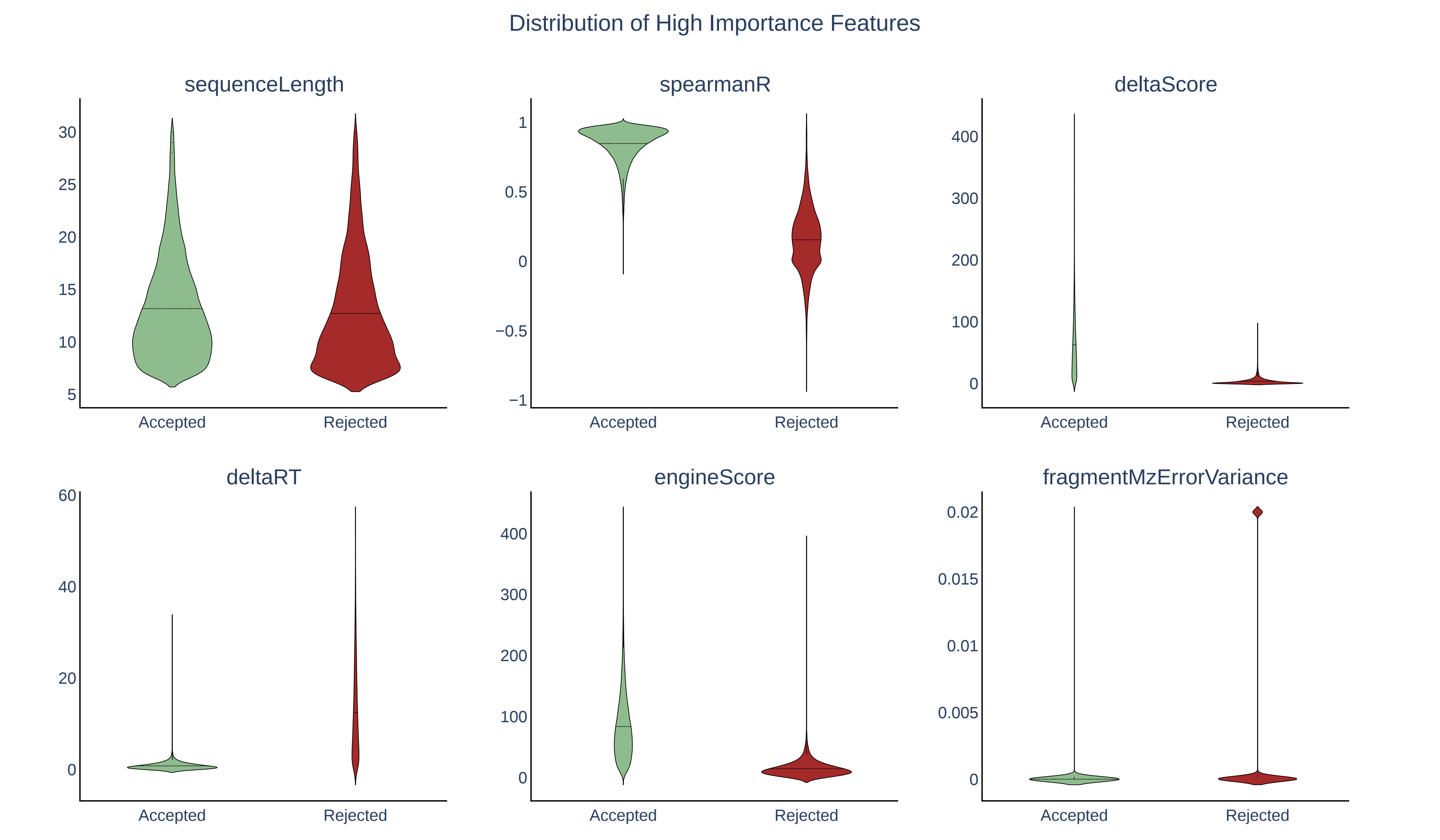

inSPIRE Performance: Number of PSMs Identified

This shows the number of PSMs discovered by inSPIRE compared to the original search engine for q-value cut offs between 0.01 and 0.1.

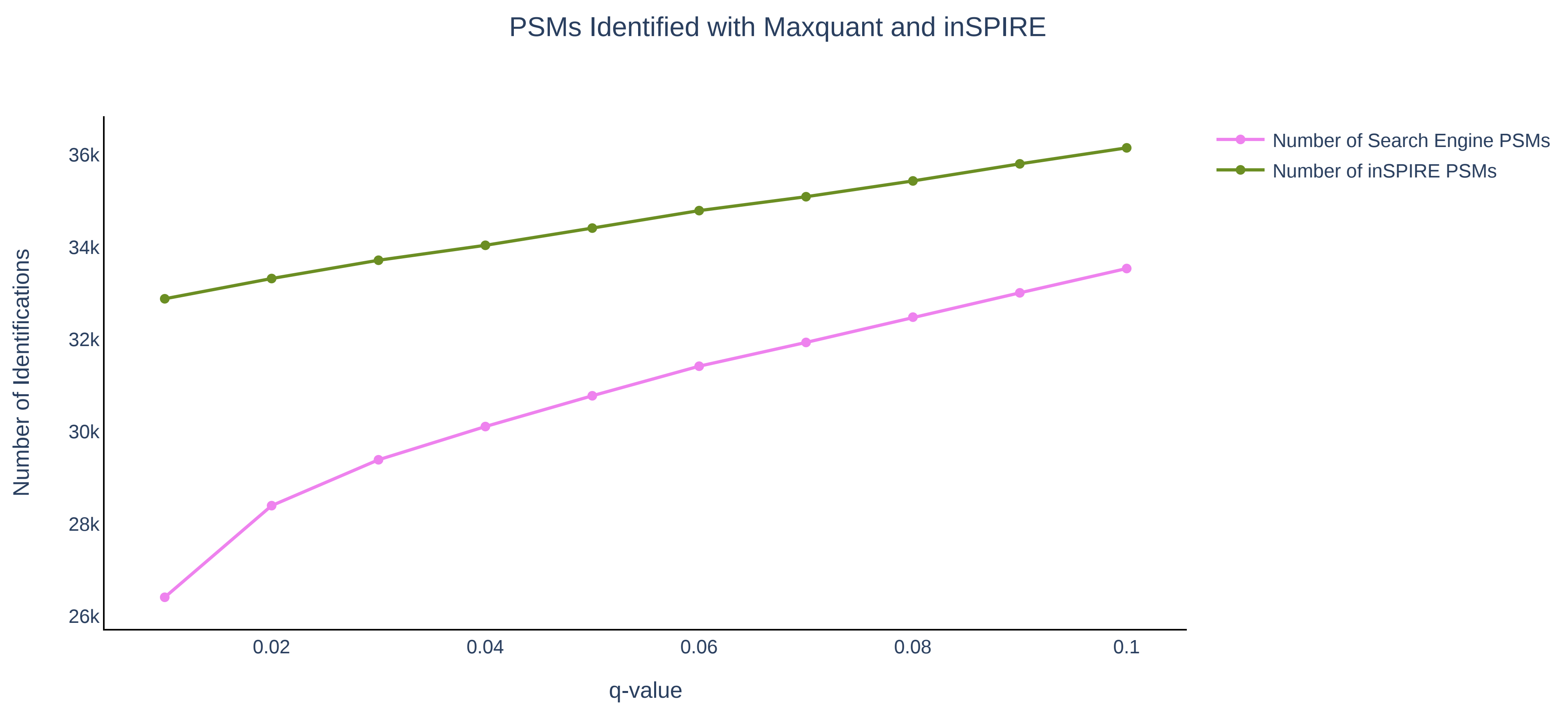

inSPIRE Report for Tryptic K562 Gencode

inSPIRE Settings Used

inSPIRE Settings for Experiment Tryptic K562 Gencode:

Config	Setting
searchEngine	maxquant
scansFormat	mgf
spectralPredictor	prosit
deltaMethod	predictor
rescoreMethod	percolator
searchResults	section_1_data/maxQuant/tryptic_gencode/txt/msms.txt
scansFolder	section_1_data/scans
outputFolder	spire_1_tryptic_gencode/output
collisionEnergy	29
mzAccuracy	0.02
mzUnits	Da
fixedModifications	['Carbamidomethylation']
forceReload	False
falseDiscoveryRate	0.01
excludeFeatures	[]
includeFeatures	None
reduce	False
filterCysteine	False
dropUnknownPTMs	True
useBindingAffinity	None

Selected Features and Importance

The table below shows the importance of the final feature set used by percolator. Strong positive values (highlighted in green) may indicate that higher feature values are more common among target PSMs while strongly negative values (highlighted in red) may indicate that higher feature values are more common among decoy PSMs. However, it is also possible that a feature like searchEngineScore ends up with a negative coefficient simply because it is so strongly correlated to a more powerful feature like deltaScore which has a strong positive coefficient.

Weights of Features Selected by inSPIRE.

feature	weightFold1	weightFold2	weightFold3	averageWeight
sequenceLength	1.0498	1.5497	1.2525	1.284
spearmanR	0.8285	1.1416	0.9766	0.982
deltaScore	0.8709	1.1046	0.7991	0.925
spectralAngle	0.5971	1.1618	0.522	0.76
maxMatchedCoverage	0.6205	0.4863	0.8378	0.648
nMajorMatchedDivFrags	0.2934	0.4218	0.2962	0.337
matchedCoverage	0.2147	0.4378	0.2265	0.293
nMinorMatchedDivFrags	0.266	0.3443	0.2278	0.279
maxPrositDelta	0.1812	0.2816	0.2514	0.238
spearmanMajorIons	0.221	0.1854	0.283	0.23
prositDeltaMedian	0.1353	0.1226	0.3419	0.2
fracUnique	0.1271	0.237	0.1993	0.188
maxTypeSpectralAngle	0.0985	0.2105	0.2359	0.182
avgResidueMass	0.1286	0.1728	0.2112	0.171
minMatchedCoverage	0.0686	0.0676	0.2904	0.142
medianAbsoluteError	0.1236	0.029	0.1486	0.1
nRepeatedResidues	0.0487	0.1002	0.0661	0.072
nVarMods	0.035	0.0799	-0.0268	0.029
yIsDominantIonSeries	0.018	0.0125	0.0575	0.029
predNotFoundCoverage	0.0853	-0.0195	0.021	0.029
nDeltasAboveThreshold	0.0473	0.0455	-0.05	0.014
nMajorNotMatchableDivFrags	-0.0055	0.0258	-0.0035	0.006
prositDeltaQuartile3	0.0518	0.0751	-0.1246	0.001
nLossIonsDivFrags	-0.0097	-0.0235	0.0304	-0.001
missedCleavages	-0.0477	-0.0076	0.0476	-0.003
charge	0.0294	-0.117	0.0022	-0.028
fromChimera	-0.0725	-0.0377	-0.0158	-0.042
fracC	-0.0657	-0.0251	-0.0539	-0.048
nMinorNotMatchableDivFrags	-0.0145	0.0125	-0.1882	-0.063
nDeltasAboveZero	-0.0765	-0.0582	-0.0748	-0.07
minPrositDelta	-0.0438	-0.0408	-0.1281	-0.071
fracMatchedKR	-0.0314	-0.13	-0.0818	-0.081
fracKR	-0.0543	-0.1009	-0.1722	-0.109
medianFragmentMzError	-0.097	-0.292	-0.2796	-0.223
prositDeltaQuartile1	-0.356	-0.376	-0.2912	-0.341
spectrumDensity	-0.3025	-0.4796	-0.2763	-0.353
seqLenMeanDiff	-0.5281	-0.7395	-0.561	-0.61
pearsonR	-0.4233	-0.9861	-0.5009	-0.637
fragmentMzErrorVariance	-0.0473	-2.0548	-0.0628	-0.722
engineScore	-0.968	-1.151	-1.082	-1.067
deltaRT	-1.6371	-1.4512	-1.1854	-1.425
m0	0.0548	-0.0419	0.252	0.088

Feature Distributions

These Violin Plots show the distributions of the three most heavily positive and heavily negative weighted features for accepted and rejected PSMs.

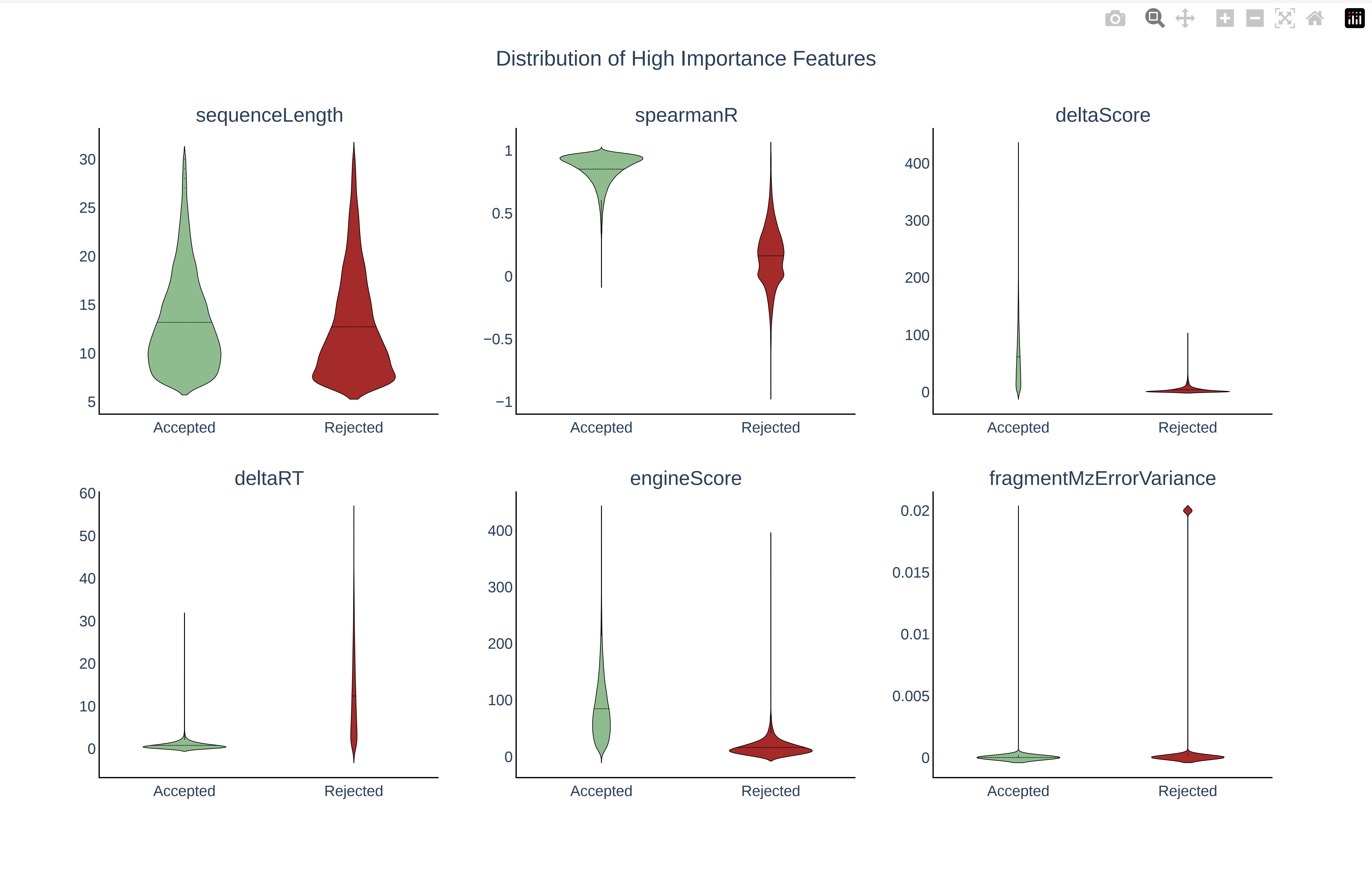

inSPIRE Performance: Number of PSMs Identified

This shows the number of PSMs discovered by inSPIRE compared to the original search engine for q-value cut offs between 0.01 and 0.1.

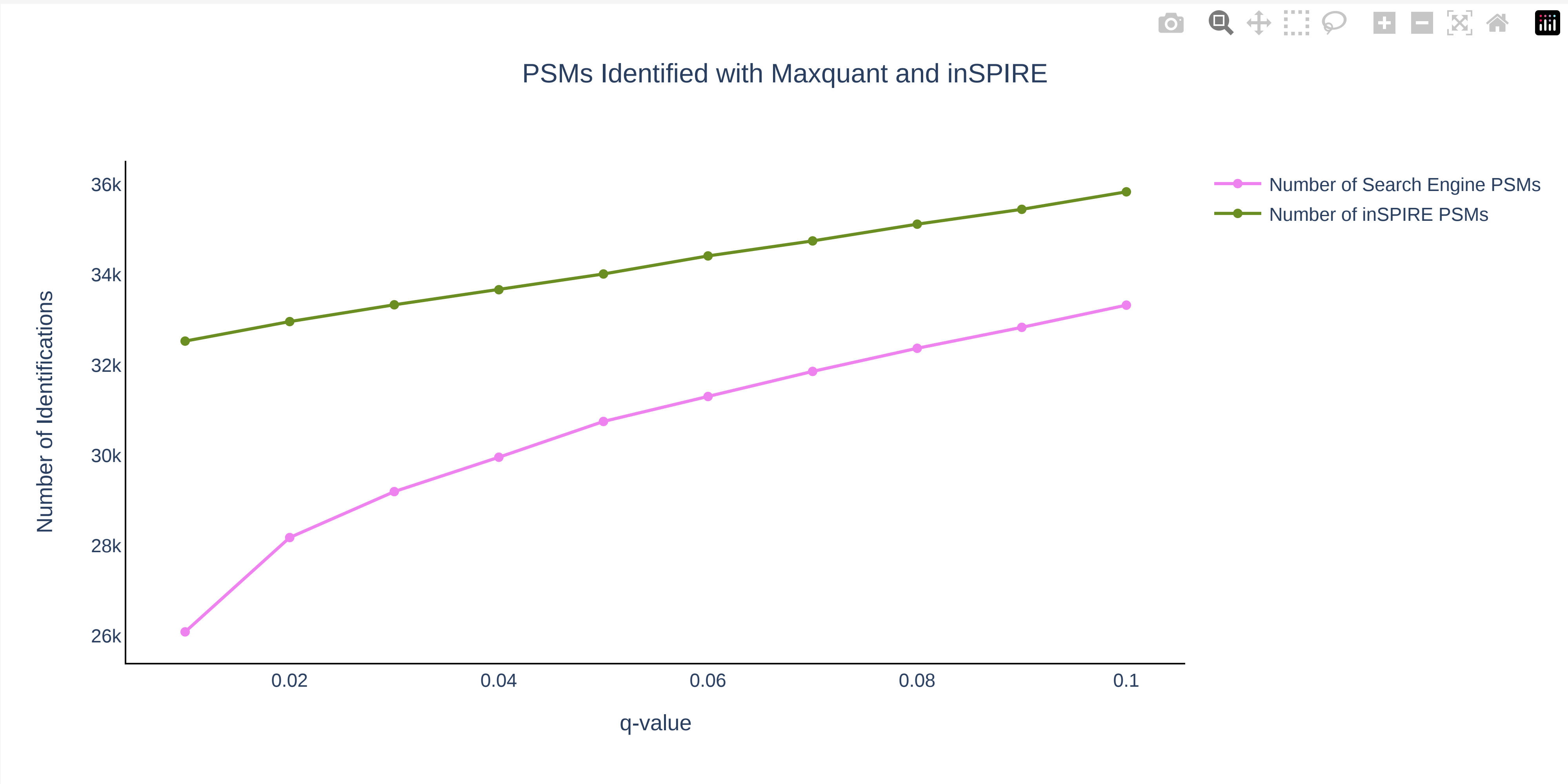

Supplement: Supplemental File S3 [file mmc3.pdf]
