# Supplementary material for: inSPIRE: An Open-Source Tool for Increased Mass Spectrometry Identification Rates Using Prosit Spectral Prediction
Source: Mol Cell Proteomics. 2022 Oct 21;21(12):100432. doi: 10.1016/j.mcpro.2022.100432 (PMC9720494; doi:10.1016/j.mcpro.2022.100432)
Supplement: Supplemental File S4 [file mmc4.pdf]

File S4. Example of inSPIRE spectral plotting. This is an example of the PDF file which can be obtained from the inSPIRE plotSpectra utility for PSMs of varying quality. Provided are mirror spectra where the spectrum above the y-axis is the experimental spectrum and the spectrum below the y-axis is the Prosit predicted spectrum.

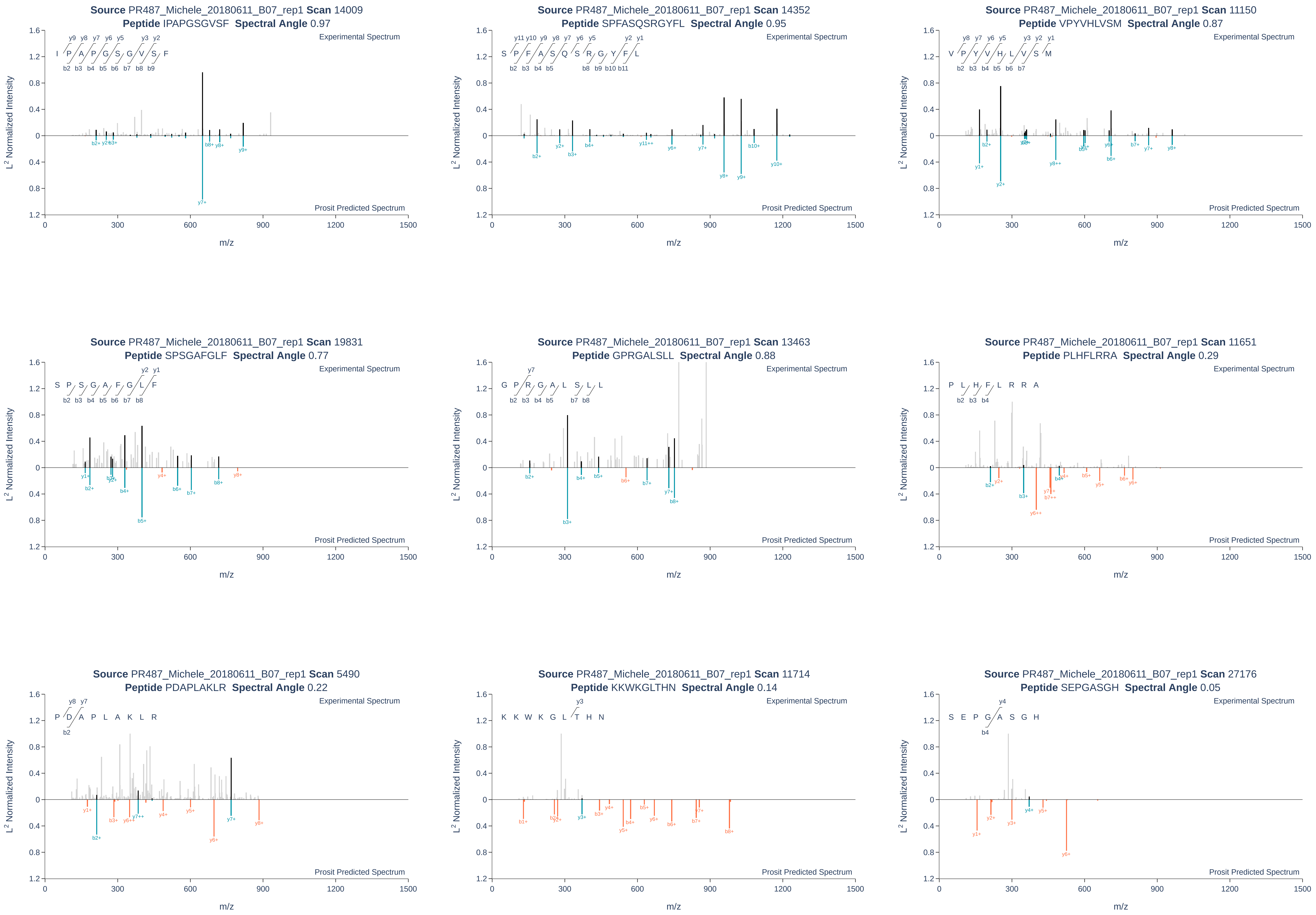

Source PR487_Michele_20180611_B07_rep1 Scan 14009
Peptide IPAPGSGVSF Spectral Angle 0.97
Source PR487_Michele_20180611_B07_rep1 Scan 14352
Peptide SPFASQSRGYFL Spectral Angle 0.95
Source PR487_Michele_20180611_B07_rep1 Scan 11150
Peptide VPYVHLVSM Spectral Angle 0.87
Source PR487_Michele_20180611_B07_rep1 Scan 19831
Peptide SPSGAFGLF Spectral Angle 0.77
Source PR487_Michele_20180611_B07_rep1 Scan 13463
Peptide GPRGALSLL Spectral Angle 0.88
Source PR487_Michele_20180611_B07_rep1 Scan 11651
Peptide PLHFLRRA Spectral Angle 0.29
Source PR487_Michele_20180611_B07_rep1 Scan 5490
Peptide PDAPLAKLR Spectral Angle 0.22
Source PR487_Michele_20180611_B07_rep1 Scan 11714
Peptide KKWKGLTHN Spectral Angle 0.14
Source PR487_Michele_20180611_B07_rep1 Scan 27176
Peptide SEPGASGH Spectral Angle 0.05
Experimental Spectrum
Prosit Predicted Spectrum
L² Normalized Intensity
m/z
