# Supplementary material for: inSPIRE: An Open-Source Tool for Increased Mass Spectrometry Identification Rates Using Prosit Spectral Prediction
Source: Mol Cell Proteomics. 2022 Oct 21;21(12):100432. doi: 10.1016/j.mcpro.2022.100432 (PMC9720494; doi:10.1016/j.mcpro.2022.100432)
Supplement: Supplemental Figures S1–S14 and Tables S1–S4 [file mmc12.pdf]

inSPIRE: An open-source tool for increased mass spectrometry identification rates using Prosit spectral prediction

John A. Cormican[1], Yehor Horokhovskyi1, Wai Tuck Soh[1], Michele Mishto[2,3,$,*] Juliane Liepe[1,$,*]

1 Max-Planck-Institute for Multidisciplinary Sciences (MPI-NAT), 37077 Göttingen, Germany

2 Centre for Inflammation Biology and Cancer Immunology (CIBCI) & Peter Gorer Department of Immunobiology, King's College London, SE1 1UL London, United Kingdom

3 The Francis Crick Institute, WC2A 3LY London, United Kingdom

* Correspondence to: michele.mishto@kcl.ac.uk, jliepe@mpinat.mpg.de.

$ Authors equally contributed.

Table S1	List of all features available in inSPIRE
Table S2	Datasets used to train Prosit-*delta* predictor
Table S3	Features used by the Prosit-delta predictor
Table S4	MS files used in each figure
Table S5	Search result files
Figure S1	Timing of Prosit MS2 spectral prediction via inSPIRE (CPU) and the original Prosit library (GPU only)
Figure S2	Incremental PSMs identified by inSPIRE and baseline rescoring pipelines
Figure S3	Incremental PSMs identified by inSPIRE and Prosit rescoring pipelines
Figure S4	Incremental PSMs identified by inSPIRE-affinity and standard inSPIRE pipelines
Figure S5	Incremental PSMs identified by inSPIRE-MS^{2}PIP and MS^{2}Rescore pipelines
Figure S6	Sequence logo plots for 9 residue-long peptides Identified by inSPIRE and Prosit rescoring for all HLA-I Immunopeptidomes analyzed
Figure S7	Number of PSMs identified by inSPIRE, inSPIRE-affinity and Prosit Rescoring across a variety of HLA-I monoallelic cell lines
Figure S8	Percentage of peptides predicted to bind the cognate HLA-I complex, and identified by inSPIRE, inSPIRE-affinity and Prosit Rescoring in a variety of HLA-I monoallelic immunopeptidomes
Figure S9	Overlap of peptides identified by Mascot, MaxQuant and PEAKS search engines with or without rescoring with inSPIRE
Figure S10	Distribution of MS1 ion peak intensity for PSMs identified by original search engine and inSPIRE, compared to PSMs identified by inSPIRE only
Figure S11	Distribution of sequence length, charge state and sequence motifs in the Prosit-*delta* training dataset
Figure S12	Feature importance in the Prosit-*delta* Predictor.
Figure S13	Impact of Prosit-*delta* feature on inSPIRE's PSM yield in HLA-I immunopeptidomics with an FDR range of 1-5%
Figure S14	Validation of peptide identification by applying inSPIRE variants with or without Prosit-*delta* features

**Feature Name**	**Description**
engineScore	The score from the original search engine.
deltaScore	The difference between the highest scoring and second highest scoring PSM for a spectrum (filled with 0 for PEAKS DB).
sequenceLength	The length of the peptide sequence (number of residues).
seqLenMeanDiff	The difference between the length of the peptide sequence and the mean peptide length in the dataset.
charge	The precursor charge of the peptide.
avgResidueMass	The mass of the peptide divided by sequenceLength.
nVarMods	The number of variable modifications on the peptide sequence.
spectralAngle	The spectral angle between the Prosit predicted spectrum and experimental spectrum.
deltaRT	The absolute difference between the fitted Prosit predicted retention time and the experimentally observed spectrum.
bindingAffinity (if specified)	The minimum predicted MHC binding affinity from NetMHCpan for the sequence.
spearmanR	The Spearman correlation between the Prosit predicted spectrum and experimental spectrum.
pearsonR	The Pearson correlation between the Prosit predicted spectrum and experimental spectrum.
spearmanMajorIons	The Spearman correlation between the Prosit predicted spectrum and experimental spectrum where the Prosit prediction is greater than or equal to 10% of predicted L2 norm.
medianAbsoluteError	The median absolute error between the Prosit predicted intensities and experimentally observed intensities.
matchedCoverage	The fraction of fragmentation positions in the sequence for which there is at least one Prosit matched ion.
maxMatchedCoverage	The fraction of fragmentation positions in the sequence for which there is at least one Prosit matched ion for the ion series where Prosit predicts more peaks.
minMatchedCoverage	The fraction of fragmentation positions in the sequence for which there is at least one Prosit matched ion for the ion series where Prosit predicts less peaks.
minPrositDelta	The minimum predicted Prosit-*delta* value.
prositDeltaQuartile1	The first quartile of predicted Prosit-*delta* values.
prositDeltaMedian	The median predicted Prosit-*delta* value.
prositDeltaQuartile3	The third quartile of predicted Prosit-*delta* values.
maxPrositDelta	The maximum predicted Prosit-*delta* value.
nDeltasAboveZero	The number of predicted Prosit-*delta* values above 0.
nDeltasAboveThreshold	The number of predicted Prosit-*delta* values above -0.1.
fracUnique	The number of unique residues in the peptide divided by the length of the sequence.
nRepeatedResidues	The number of times the same amino acid occurs repeatedly within the peptide.
medianFragmentMzError	The median error in Daltons between the m/z values of the Prosit predicted peaks and experimentally observed m/z values.
fragmentMzErrorVariance	The variance in the m/z error between the Prosit predicted peaks and experimentally observed m/z values.
nMajorMatchedDivFrags	The number of matched peaks where the Prosit prediction is greater than or equal to 10% of predicted L2 norm divided by the number of fragmentation positions in the sequence.

nMinorMatchedDivFrags	The number of matched peaks where the Prosit prediction is less than 10% of predicted L2 norm divided by the number of fragmentation positions in the sequence.
nMajorNotMatchableDivFrags	The number of peaks in the experimental peaks where the intensity is greater than or equal to 10% of the L2 norm of all observed intensities divided by the number of fragmentation positions in the sequence.
nMinorNotMatchableDivFrags	The number of peaks in the experimental peaks where the intensity is less than 10% of the L2 norm of all observed intensities divided by the number of fragmentation positions in the sequence.
maxTypeSpectralAngle	The spectral angle for the ion series for which Prosit predicts the largest number of intensities.
yIsDominantIonSeries	Flag indicating if there are more predicted peaks for the y-ions rather than the b-ions.
nLossIonsDivFrags	The number of possible matched ions from neutral losses divided by the number of fragmentation positions in the sequence.
spectrumDensity	The number of peaks in the experimental spectrum divided by the m/z range of the observed peaks.
fracC	The fraction of residues which are cysteine (confounding variable due to Prosit assumption of fixed carbamidomethylation).
fracKR	The fraction of residues which are either arginine or lysine (confounding variables for tryptic datasets and because of the greater intensities for fragments containing K or R).
fracMatchedKR	The fraction of matched fragment ions containing arginine or lysine divided by the number of possible fragment ions.
missedCleavages	The number of missed cleavages in the sequence.
fromChimera	Flag indicating if the PSM comes from a chimeric spectrum.

Table S1. List of all features available in inSPIRE. All of these features are used by default in the rescoring pipeline. The user can filter these columns as required for their dataset. The number of fragmentation positions of a peptide is defined as the sequence length minus 1.

Reference	Raw Files	CE	Number of PSMs Used
PXD015489	A1101_Uninfected<br>B5703_Uninfected<br>C8166_Uninfected	32	129,682
PXD005231	20160513_TIL1_R1<br>20160513_TIL2_R1<br>20160513_TIL3_R1<br>20160513_TIL4_R1<br>20160517_DC1W6_R1<br>20160517_DC3W6_R1<br>20160517_DC5W6_R1<br>20160517_GD149-2_R1<br>20160517_GD149-4_R1<br>20160617_PD42_w6_S1_R1<br>20160730_QEh1_LC1_HuPa_SA_Apher-1_Amino_1<br>20160730_QEh1_LC1_HuPa_SA_Apher-6_Amino_1<br>20160823_QEh1_LC2_HuPa_SA_HLApI_CM647_2_MG_1<br>20160823_QEh1_LC2_HuPa_SA_HLApI_CM647_3_MG_1<br>20160823_QEh1_LC2_HuPa_SA_HLApI_RA957_1_MG_1<br>20160823_QEh1_LC2_HuPa_SA_HLApI_RA957_2_MG_1<br>20160902_QEh1_LC2_CHC_SA_HLApI_MD155_1<br>20160902_QEh1_LC2_CHC_SA_HLApI_pooledTIL3_1	34	434,184
PXD010595	02445a_BE1-TUM_HLA_49_01_01-3xHCD-1h-R1<br>02445a_BG10-TUM_HLA_82_01_01-3xHCD-1h-R1<br>02445b_BG5-TUM_HLA_77_01_01-3xHCD-1h-R4<br>02445d_BA10-TUM_HLA_10_01_01-3xHCD-1h-R4<br>02445d_BA11-TUM_HLA_11_01_01-3xHCD-1h-R4<br>02445d_BA12-TUM_HLA_12_01_01-3xHCD-1h-R4<br>02445d_BA1-TUM_HLA_1_01_01-3xHCD-1h-R4<br>02445d_BA2-TUM_HLA_2_01_01-3xHCD-1h-R4<br>02445d_BA3-TUM_HLA_3_01_01-3xHCD-1h-R4<br>02445d_BA4-TUM_HLA_4_01_01-3xHCD-1h-R4<br>02445d_BA5-TUM_HLA_5_01_01-3xHCD-1h-R4<br>02445d_BA6-TUM_HLA_6_01_01-3xHCD-1h-R4<br>02445d_BA7-TUM_HLA_7_01_01-3xHCD-1h-R4<br>02445d_BA8-TUM_HLA_8_01_01-3xHCD-1h-R4<br>02445d_BA9-TUM_HLA_9_01_01-3xHCD-1h-R4<br>02445d_BB10-TUM_HLA_22_01_01-3xHCD-1h-R4<br>02445d_BB11-TUM_HLA_23_01_01-3xHCD-1h-R4<br>02445d_BB12-TUM_HLA_24_01_01-3xHCD-1h-R4<br>02445d_BB1-TUM_HLA_13_01_01-3xHCD-1h-R4<br>02445d_BB2-TUM_HLA_14_01_01-3xHCD-1h-R4<br>02445d_BB3-TUM_HLA_15_01_01-3xHCD-1h-R4<br>02445d_BB4-TUM_HLA_16_01_01-3xHCD-1h-R4<br>02445d_BB5-TUM_HLA_17_01_01-3xHCD-1h-R4<br>02445d_BB6-TUM_HLA_18_01_01-3xHCD-1h-R4<br>02445d_BB7-TUM_HLA_19_01_01-3xHCD-1h-R4<br>02445d_BB8-TUM_HLA_20_01_01-3xHCD-1h-R4<br>02445d_BB9-TUM_HLA_21_01_01-3xHCD-1h-R4<br>02445d_BC10-TUM_HLA_34_01_01-3xHCD-1h-R4<br>02445d_BC11-TUM_HLA_35_01_01-3xHCD-1h-R4<br>02445d_BC12-TUM_HLA_36_01_01-3xHCD-1h-R4<br>02445d_BC1-TUM_HLA_25_01_01-3xHCD-1h-R4<br>02445d_BC2-TUM_HLA_26_01_01-3xHCD-1h-R4<br>02445d_BC3-TUM_HLA_27_01_01-3xHCD-1h-R4	25<br>30<br>35	608,152

	02445d_BC4-TUM_HLA_28_01_01-3xHCD-1h-R4<br>02445d_BC5-TUM_HLA_29_01_01-3xHCD-1h-R4<br>02445d_BC6-TUM_HLA_30_01_01-3xHCD-1h-R4<br>02445d_BC7-TUM_HLA_31_01_01-3xHCD-1h-R4<br>02445d_BC8-TUM_HLA_32_01_01-3xHCD-1h-R4<br>02445d_BC9-TUM_HLA_33_01_01-3xHCD-1h-R4<br>02445d_BD10-TUM_HLA_46_01_01-3xHCD-1h-R4<br>02445d_BD11-TUM_HLA_47_01_01-3xHCD-1h-R4<br>02445d_BD12-TUM_HLA_48_01_01-3xHCD-1h-R4<br>02445d_BD1-TUM_HLA_37_01_01-3xHCD-1h-R4<br>02445d_BD2-TUM_HLA_38_01_01-3xHCD-1h-R4<br>02445d_BD3-TUM_HLA_39_01_01-3xHCD-1h-R4<br>02445d_BD4-TUM_HLA_40_01_01-3xHCD-1h-R4<br>02445d_BD5-TUM_HLA_41_01_01-3xHCD-1h-R4<br>02445d_BD6-TUM_HLA_42_01_01-3xHCD-1h-R4<br>02445d_BD7-TUM_HLA_43_01_01-3xHCD-1h-R4<br>02445d_BD8-TUM_HLA_44_01_01-3xHCD-1h-R4<br>02445d_BD9-TUM_HLA_45_01_01-3xHCD-1h-R4<br>02445d_BE10-TUM_HLA_58_01_01-3xHCD-1h-R4<br>02445d_BE11-TUM_HLA_59_01_01-3xHCD-1h-R4<br>02445d_BE12-TUM_HLA_60_01_01-3xHCD-1h-R4<br>02445d_BE2-TUM_HLA_50_01_01-3xHCD-1h-R4<br>02445d_BE3-TUM_HLA_51_01_01-3xHCD-1h-R4<br>02445d_BE4-TUM_HLA_52_01_01-3xHCD-1h-R4<br>02445d_BE5-TUM_HLA_53_01_01-3xHCD-1h-R4<br>02445d_BE6-TUM_HLA_54_01_01-3xHCD-1h-R4<br>02445d_BE7-TUM_HLA_55_01_01-3xHCD-1h-R4<br>02445d_BE8-TUM_HLA_56_01_01-3xHCD-1h-R4<br>02445d_BE9-TUM_HLA_57_01_01-3xHCD-1h-R4<br>02445d_BF10-TUM_HLA_70_01_01-3xHCD-1h-R4<br>02445d_BF11-TUM_HLA_71_01_01-3xHCD-1h-R4<br>02445d_BF12-TUM_HLA_72_01_01-3xHCD-1h-R4<br>02445d_BF1-TUM_HLA_61_01_01-3xHCD-1h-R4<br>02445d_BF2-TUM_HLA_62_01_01-3xHCD-1h-R4<br>02445d_BF3-TUM_HLA_63_01_01-3xHCD-1h-R4<br>02445d_BF4-TUM_HLA_64_01_01-3xHCD-1h-R4<br>02445d_BF5-TUM_HLA_65_01_01-3xHCD-1h-R4<br>02445d_BF6-TUM_HLA_66_01_01-3xHCD-1h-R4<br>02445d_BF7-TUM_HLA_67_01_01-3xHCD-1h-R4<br>02445d_BF8-TUM_HLA_68_01_01-3xHCD-1h-R4<br>02445d_BF9-TUM_HLA_69_01_01-3xHCD-1h-R4<br>02445d_BG11-TUM_HLA_83_01_01-3xHCD-1h-R4<br>02445d_BG12-TUM_HLA_84_01_01-3xHCD-1h-R4<br>02445d_BG1-TUM_HLA_73_01_01-3xHCD-1h-R4<br>02445d_BG2-TUM_HLA_74_01_01-3xHCD-1h-R4<br>02445d_BG3-TUM_HLA_75_01_01-3xHCD-1h-R4<br>02445d_BG4-TUM_HLA_76_01_01-3xHCD-1h-R4<br>02445d_BG6-TUM_HLA_78_01_01-3xHCD-1h-R4<br>02445d_BG7-TUM_HLA_79_01_01-3xHCD-1h-R4<br>02445d_BG8-TUM_HLA_80_01_01-3xHCD-1h-R4<br>02445d_BG9-TUM_HLA_81_01_01-3xHCD-1h-R4<br>02445d_BH10-TUM_HLA_94_01_01-3xHCD-1h-R4<br>02445d_BH11-TUM_HLA_95_01_01-3xHCD-1h-R4<br>02445d_BH12-TUM_HLA_96_01_01-3xHCD-1h-R4<br>02445d_BH1-TUM_HLA_85_01_01-3xHCD-1h-R4<br>02445d_BH2-TUM_HLA_86_01_01-3xHCD-1h-R4		

	02445d_BH3-TUM_HLA_87_01_01-3xHCD-1h-R4<br>02445d_BH4-TUM_HLA_88_01_01-3xHCD-1h-R4<br>02445d_BH5-TUM_HLA_89_01_01-3xHCD-1h-R4<br>02445d_BH6-TUM_HLA_90_01_01-3xHCD-1h-R4<br>02445d_BH7-TUM_HLA_91_01_01-3xHCD-1h-R4<br>02445d_BH8-TUM_HLA_92_01_01-3xHCD-1h-R4<br>02445d_BH9-TUM_HLA_93_01_01-3xHCD-1h-R4<br>02446d_GA10-TUM_HLA_106_01_01-3xHCD-1h-R4<br>02446d_GA11-TUM_HLA_107_01_01-3xHCD-1h-R4<br>02446d_GA12-TUM_HLA_108_01_01-3xHCD-1h-R4<br>02446d_GA1-TUM_HLA_97_01_01-3xHCD-1h-R4<br>02446d_GA2-TUM_HLA_98_01_01-3xHCD-1h-R4<br>02446d_GA3-TUM_HLA_99_01_01-3xHCD-1h-R4<br>02446d_GA4-TUM_HLA_100_01_01-3xHCD-1h-R4<br>02446d_GA5-TUM_HLA_101_01_01-3xHCD-1h-R4<br>02446d_GA6-TUM_HLA_102_01_01-3xHCD-1h-R4<br>02446d_GA7-TUM_HLA_103_01_01-3xHCD-1h-R4<br>02446d_GA8-TUM_HLA_104_01_01-3xHCD-1h-R4<br>02446d_GA9-TUM_HLA_105_01_01-3xHCD-1h-R4<br>02446d_GB10-TUM_HLA_118_01_01-3xHCD-1h-R4<br>02446d_GB11-TUM_HLA_119_01_01-3xHCD-1h-R4<br>02446d_GB12-TUM_HLA_120_01_01-3xHCD-1h-R4<br>02446d_GB1-TUM_HLA_109_01_01-3xHCD-1h-R4<br>02446d_GB2-TUM_HLA_110_01_01-3xHCD-1h-R4<br>02446d_GB3-TUM_HLA_111_01_01-3xHCD-1h-R4<br>02446d_GB4-TUM_HLA_112_01_01-3xHCD-1h-R4<br>02446d_GB5-TUM_HLA_113_01_01-3xHCD-1h-R4<br>02446d_GB6-TUM_HLA_114_01_01-3xHCD-1h-R4<br>02446d_GB7-TUM_HLA_115_01_01-3xHCD-1h-R4<br>02446d_GB8-TUM_HLA_116_01_01-3xHCD-1h-R4<br>02446d_GB9-TUM_HLA_117_01_01-3xHCD-1h-R4<br>02446d_GC10-TUM_HLA_130_01_01-3xHCD-1h-R4<br>02446d_GC11-TUM_HLA_131_01_01-3xHCD-1h-R4<br>02446d_GC12-TUM_HLA_132_01_01-3xHCD-1h-R4<br>02446d_GC1-TUM_HLA_121_01_01-3xHCD-1h-R4<br>02446d_GC2-TUM_HLA_122_01_01-3xHCD-1h-R4<br>02446d_GC3-TUM_HLA_123_01_01-3xHCD-1h-R4<br>02446d_GC4-TUM_HLA_124_01_01-3xHCD-1h-R4<br>02446d_GC5-TUM_HLA_125_01_01-3xHCD-1h-R4<br>02446d_GC6-TUM_HLA_126_01_01-3xHCD-1h-R4<br>02446d_GC7-TUM_HLA_127_01_01-3xHCD-1h-R4<br>02446d_GC8-TUM_HLA_128_01_01-3xHCD-1h-R4<br>02446d_GC9-TUM_HLA_129_01_01-3xHCD-1h-R4<br>02446d_GD10-TUM_HLA_142_01_01-3xHCD-1h-R4<br>02446d_GD11-TUM_HLA_143_01_01-3xHCD-1h-R4<br>02446d_GD12-TUM_HLA_144_01_01-3xHCD-1h-R4<br>02446d_GD1-TUM_HLA_133_01_01-3xHCD-1h-R4<br>02446d_GD2-TUM_HLA_134_01_01-3xHCD-1h-R4<br>02446d_GD3-TUM_HLA_135_01_01-3xHCD-1h-R4<br>02446d_GD4-TUM_HLA_136_01_01-3xHCD-1h-R4<br>02446d_GD5-TUM_HLA_137_01_01-3xHCD-1h-R4<br>02446d_GD6-TUM_HLA_138_01_01-3xHCD-1h-R4<br>02446d_GD7-TUM_HLA_139_01_01-3xHCD-1h-R4<br>02446d_GD8-TUM_HLA_140_01_01-3xHCD-1h-R4<br>02446d_GD9-TUM_HLA_141_01_01-3xHCD-1h-R4<br>02446d_GE10-TUM_HLA_154_01_01-3xHCD-1h-R4		

	02446d_GE11-TUM_HLA_155_01_01-3xHCD-1h-R4<br>02446d_GE12-TUM_HLA_156_01_01-3xHCD-1h-R4<br>02446d_GE1-TUM_HLA_145_01_01-3xHCD-1h-R4<br>02446d_GE2-TUM_HLA_146_01_01-3xHCD-1h-R4<br>02446d_GE3-TUM_HLA_147_01_01-3xHCD-1h-R4<br>02446d_GE4-TUM_HLA_148_01_01-3xHCD-1h-R4<br>02446d_GE5-TUM_HLA_149_01_01-3xHCD-1h-R4<br>02446d_GE6-TUM_HLA_150_01_01-3xHCD-1h-R4<br>02446d_GE7-TUM_HLA_151_01_01-3xHCD-1h-R4<br>02446d_GE8-TUM_HLA_152_01_01-3xHCD-1h-R4<br>02446d_GE9-TUM_HLA_153_01_01-3xHCD-1h-R4<br>02446d_GF10-TUM_HLA_166_01_01-3xHCD-1h-R4<br>02446d_GF11-TUM_HLA_167_01_01-3xHCD-1h-R4<br>02446d_GF12-TUM_HLA_168_01_01-3xHCD-1h-R4<br>02446d_GF1-TUM_HLA_157_01_01-3xHCD-1h-R4<br>02446d_GF2-TUM_HLA_158_01_01-3xHCD-1h-R4<br>02446d_GF3-TUM_HLA_159_01_01-3xHCD-1h-R4<br>02446d_GF4-TUM_HLA_160_01_01-3xHCD-1h-R4<br>02446d_GF5-TUM_HLA_161_01_01-3xHCD-1h-R4<br>02446d_GF6-TUM_HLA_162_01_01-3xHCD-1h-R4<br>02446d_GF7-TUM_HLA_163_01_01-3xHCD-1h-R4<br>02446d_GF8-TUM_HLA_164_01_01-3xHCD-1h-R4<br>02446d_GF9-TUM_HLA_165_01_01-3xHCD-1h-R4<br>02446d_GG10-TUM_HLA_178_01_01-3xHCD-1h-R4<br>02446d_GG1-TUM_HLA_169_01_01-3xHCD-1h-R4<br>02446d_GG2-TUM_HLA_170_01_01-3xHCD-1h-R4<br>02446d_GG3-TUM_HLA_171_01_01-3xHCD-1h-R4<br>02446d_GG4-TUM_HLA_172_01_01-3xHCD-1h-R4<br>02446d_GG5-TUM_HLA_173_01_01-3xHCD-1h-R4<br>02446d_GG6-TUM_HLA_174_01_01-3xHCD-1h-R4<br>02446d_GG7-TUM_HLA_175_01_01-3xHCD-1h-R4<br>02446d_GG8-TUM_HLA_176_01_01-3xHCD-1h-R4<br>02446d_GG9-TUM_HLA_177_01_01-3xHCD-1h-R4		

Table S2. Datasets used to train Prosit-*delta* predictor. Provided are the RAW files used from each of the PRIDE repositories used to provide training data for the Prosit-*delta* predictor.

**Feature Name**	**Description**
spectralAngle	The spectral angle of the original PSM.
blosumC	The blosum 7.1 encoding of the amino acid at the C-terminus side of the residue position switch.
blosumN	The blosum 7.1 encoding of the amino acid at the N-terminus side of the residue position switch
nTermDist	The distance number of residues between the permutation position and the N-terminus of the peptide.
cTermDist	The distance number of residues between the permutation position and the C-terminus of the peptide.
charge	The precursor charge of the peptide.
yErrsAtLoc	The error between predicted and observed normalized intensity values for y-ions at the position of permutation.
bErrsAtLoc	The error between predicted and observed normalized intensity values for b-ions at the position of permutation.
collisionEnergy	The collision energy setting used in the Prosit prediction.
bPrositIntesAtLoc	The sum of Prosit predicted intensities for b-ions at the fragmentation position where the residues have been flipped.
yPrositIntesAtLoc	The sum of Prosit predicted intensities for y-ions at the fragmentation position where the residues have been flipped.
bPrositIntesAtC	The sum of Prosit predicted intensities for b-ions at the fragmentation position towards the C terminal of the peptide.
yPrositIntesAtN	The sum of Prosit predicted intensities for y-ions at the fragmentation position towards the N terminal of the peptide.
bMatchedIntesAtLoc	The sum of matched intensities for b-ions at the fragmentation position where the residues have been flipped.
yMatchedIntesAtLoc	The sum of matched intensities for y-ions at the fragmentation position where the residues have been flipped.
bMatchedIntesAtC	The sum of matched intensities for b-ions at the fragmentation position towards the C terminal of the peptide.
yMatchedIntesAtN	The sum of matched intensities for y-ions at the fragmentation position towards the N terminal of the peptide.
cOxidation	Flag indicating oxidation of the amino acid being swapped on the C-terminus side.
nOxidation	Flag indicating oxidation of the amino acid being swapped on the N-terminus side.
flipYNewIntensity	The sum of the normalized intensities matched to m/z values of potential y-ions after permutation.
flipBNewIntensity	The sum of the normalized intensities matched to m/z values of potential b-ions after permutation.
matchedCoverage	The fraction of fragmentation positions in the sequence for which there is at least one Prosit matched ion.

Table S3. Features used by the Prosit-*delta* predictor. This feature set is used to predict the Prosit-*delta* values. These values are passed to the model within inSPIRE to predict the Prosit-*delta* values for each position within the peptide.

Figure	MS files	Reference
2 (A)	PR489_Michel_S02_rep2	PXD034056
2 (B)	PR487_Michele_20180611_B07_rep1	PXD034056
2 (C)	WSoh__281021_181221_Expl3_A1_tryp_pt5ug_R1	PXD031709
3	PR489_Michele_20200228_SPL1-2_r1_20200303203802.raw<br>PR489_Michele_20200228_SPL2-2_r1.raw<br>PR489_Michele_2020316_SPL3-2_r1.raw<br>PR489_Michele_2020316_SPL4-2_r1.raw<br>PR489_Michele_2020316_SPL5-2_r1.raw<br>PR489_Michele_2020316_SPL6-2_r1.raw<br>PR489_Michele_2020316_SPL7-2_r1.raw<br>PR489_Michele_2020316_SPL8-2_r1.raw	PXD031812
4 (A-B)	WSoh__281021_181221_Expl3_A1_tryp_pt5ug_R1<br>WSoh__281021_181221_Expl3_A1_tryp_pt5ug_R2<br>WSoh__281021_181221_Expl3_A2_tryp_pt5ug_R1<br>WSoh__281021_181221_Expl3_A2_tryp_pt5ug_R2<br>WSoh__281021_181221_Expl3_A3_tryp_pt5ug_R1<br>WSoh__281021_181221_Expl3_A3_tryp_pt5ug_R2	PXD034056
4 (C-D)	PR487_Michele_20180604_B07<br>PR487_Michele_20180611_B07_rep1<br>PR487_Michele_20180611_B07_rep2	PXD031709
5 (A)	PR489_Michele_2020316_SPL3-2_r1	PXD031812
5 (B)	PR489_Michele_2020316_SPL4-2_r1	PXD031812
5 (C)	PR489_Michel_S02_rep2<br>PR487_Michele_20180611_B07_rep1	PXD015489
5 (D)	WSoh__281021_181221_Expl3_A1_tryp_pt5ug_R1	
5 (E)	WSoh__281021_181221_Expl3_A1_tryp_pt5ug_R1<br>PR487_Michele_20180611_B07_rep1<br>PR489_Michel_S02_rep2	PXD031709
S2	PR489_Michel_S02_rep2<br>PR487_Michele_20180611_B07_rep1	PXD031709
S3	PR489_Michel_S02_rep2<br>PR487_Michele_20180611_B07_rep1	PXD031709
S4	PR489_Michel_S02_rep2<br>PR487_Michele_20180611_B07_rep1	PXD031709
S5	PR489_Michel_S02_rep2<br>PR487_Michele_20180611_B07_rep1	PXD031709
S6	PR489_Michel_S02_rep2<br>PR487_Michele_20180611_B07_rep1,<br>GN20170720_SK_HLA_A3002_R1_01,<br>GN20171013_SK_HLA_A3401_R1_01,<br>GN20170719_SK_HLA_A7401_R1_01,<br>GN20171016_SK_HLA_B1302_R1_01,<br>GN20170630_SK_HLA_B4002_R1_01,<br>YE_20180531_SK_HLA_B5802_3IPs_a50mio_R1_01,<br>GN20170531_SK_HLA_C0102_R1_01,<br>GN20170602_SK_HLA_C0501_R1_01,<br>GG20170112_CRH_HLA_C1403_biorep1_techrep1<br>GN20170722_SK_HLA_G0101_R1_01,<br>GN20170722_SK_HLA_G0103_R1_01,<br>AC20171011_Broad_HLA_G0104_R1_Rep01,	PXD031709,<br>MSV000084442
S7	GN20170720_SK_HLA_A3002_R1_01,<br>GN20171013_SK_HLA_A3401_R1_01,<br>GN20170719_SK_HLA_A7401_R1_01,	MSV000084442

	GN20171016_SK_HLA_B1302_R1_01,<br>GN20170630_SK_HLA_B4002_R1_01,<br>YE_20180531_SK_HLA_B5802_3IPs_a50mio_R1_01,<br>GN20170531_SK_HLA_C0102_R1_01,<br>GN20170602_SK_HLA_C0501_R1_01,<br>GG20170112_CRH_HLA_C1403_biorep1_techrep1<br>GN20170722_SK_HLA_G0101_R1_01,<br>GN20170722_SK_HLA_G0103_R1_01,<br>AC20171011_Broad_HLA_G0104_R1_Rep01,	
S8	GN20170720_SK_HLA_A3002_R1_01,<br>GN20171013_SK_HLA_A3401_R1_01,<br>GN20170719_SK_HLA_A7401_R1_01,<br>GN20171016_SK_HLA_B1302_R1_01,<br>GN20170630_SK_HLA_B4002_R1_01,<br>YE_20180531_SK_HLA_B5802_3IPs_a50mio_R1_01,<br>GN20170531_SK_HLA_C0102_R1_01,<br>GN20170602_SK_HLA_C0501_R1_01,<br>GG20170112_CRH_HLA_C1403_biorep1_techrep1<br>GN20170722_SK_HLA_G0101_R1_01,<br>GN20170722_SK_HLA_G0103_R1_01,<br>AC20171011_Broad_HLA_G0104_R1_Rep01,	MSV000084442
S9	PR487_Michele_20180604_B07<br>PR487_Michele_20180611_B07_rep1<br>PR487_Michele_20180611_B07_rep2	PXD031709
S10	PR487_Michele_20180604_B07<br>PR487_Michele_20180611_B07_rep1<br>PR487_Michele_20180611_B07_rep2	PXD031709
S11	All files as listed in Table S5	PXD015489,<br>PXD005231,<br>PXD010595,
S12	All files as listed in Table S5	PXD015489,<br>PXD005231,<br>PXD010595,
S13	PR489_Michel_S02_rep2<br>PR487_Michele_20180611_B07_rep1	PXD031709
S14	PR489_Michel_S02_rep2<br>PR487_Michele_20180611_B07_rep1	PXD031709

Table S4. MS files used in each figure. Details of the MS files from which each of the figures has been generated.

Figure	MS files	Reference
2 (A)	a02_single_rep_rna_msms.txt<br>a02_single_rep_gencode_msms.txt	PXD034056
2 (B)	b07_single_rep_rna_msms.txt<br>b07_single_rep_gencode_msms.txt	PXD034056
2 (C)	tryptic_single_rep_rna_msms.txt<br>tryptic_single_rep_gencode_msms.txt	PXD034056
3	spl_1-2_msms.txt<br>spl_2-2_msms.txt<br>spl_3-2_msms.txt<br>spl_4-2_msms.txt<br>spl_5-2_msms.txt<br>spl_6-2_msms.txt<br>spl_7-2_msms.txt<br>spl_8-2_msms.txt	PXD034056
4 (A-B)	tryptic_rna_peaks.csv<br>tryptic_gencode_peaks.csv<br>tryptic_rna_maxquant,txt<br>tryptic_gencode_maxquant,txt<br>tryptic_rna_mascot1.csv<br>tryptic_rna_mascot2.csv<br>tryptic_rna_mascot3.csv<br>tryptic_rna_mascot4.csv<br>tryptic_rna_mascot5.csv<br>tryptic_rna_mascot6.csv<br>tryptic_rna_mascot7.csv<br>tryptic_rna_mascot8.csv<br>tryptic_rna_mascot9.csv<br>tryptic_rna_mascot10.csv<br>tryptic_rna_mascot11.csv<br>tryptic_rna_mascot12.csv<br>tryptic_gencode_mascot1.csv<br>tryptic_gencode_mascot2.csv<br>tryptic_gencode_mascot3.csv<br>tryptic_gencode_mascot4.csv<br>tryptic_gencode_mascot5.csv<br>tryptic_gencode_mascot6.csv<br>tryptic_gencode_mascot7.csv<br>tryptic_gencode_mascot8.csv<br>tryptic_gencode_mascot9.csv<br>tryptic_gencode_mascot10.csv<br>tryptic_gencode_mascot11.csv<br>tryptic_gencode_mascot12.csv	PXD034056
4 (C-D)	b07_rna_peaks.csv<br>b07_gencode_peaks.csv<br>b07_rna_maxquant.txt<br>b07_gencode_maxquant.txt<br>b07_rna_macot1.csv<br>b07_rna_macot2.csv<br>b07_rna_macot3.csv<br>b07_rna_macot4.csv<br>b07_rna_macot5.csv<br>b07_rna_macot6.csv<br>b07_gencode_macot1.csv<br>b07_gencode_macot2.csv	PXD034056

	b07_gencode_macot3.csv<br>b07_gencode_macot4.csv<br>b07_gencode_macot5.csv<br>b07_gencode_macot6.csv	
5 (C)	b07_single_rep_gencode_msms.txt<br>a02_single_rep_gencode_msms.txt	PXD034056
5 (D)	tryptic_single_rep_gencode_msms.txt	PXD034056
5 (E)	b07_single_rep_rna_msms.txt<br>b07_single_rep_gencode_msms.txt<br>a02_single_rep_rna_msms.txt<br>a02_single_rep_gencode_msms.txt<br>tryptic_single_rep_rn_msms.txt<br>tryptic_single_rep_gencode_msms.txt	PXD034056
S2	b07_single_rep_rna_msms.txt<br>b07_single_rep_gencode_msms.txt<br>a02_single_rep_rna_msms.txt<br>a02_single_rep_gencode_msms.txt	PXD034056
S3	b07_single_rep_rna_msms.txt<br>b07_single_rep_gencode_msms.txt<br>a02_single_rep_rna_msms.txt<br>a02_single_rep_gencode_msms.txt	PXD034056
S4	b07_single_rep_rna_msms.txt<br>b07_single_rep_gencode_msms.txt<br>a02_single_rep_rna_msms.txt<br>a02_single_rep_gencode_msms.txt	PXD034056
S5	b07_single_rep_rna_msms.txt<br>b07_single_rep_gencode_msms.txt<br>a02_single_rep_rna_msms.txt<br>a02_single_rep_gencode_msms.txt	PXD034056
S6	a02_single_rep_gencode_msms.txt<br>b07_single_rep_gencode_msms.txt<br>a3002_msms.txt<br>a3401_msms.txt<br>a7401_msms.txt<br>b1302_msms.txt<br>b4002_msms.txt<br>b5802_msms.txt<br>c0102_msms.txt<br>c0501_msms.txt<br>c1403_msms.txt<br>g0101_msms.txt<br>g0103_msms.txt<br>g0104_msms.txt	PXD034056
S7	a3002_msms.txt<br>a3401_msms.txt<br>a7401_msms.txt<br>b1302_msms.txt<br>b4002_msms.txt<br>b5802_msms.txt<br>c0102_msms.txt<br>c0501_msms.txt<br>c1403_msms.txt<br>g0101_msms.txt<br>g0103_msms.txt<br>g0104_msms.txt	PXD034056

S8	a3002_msms.txt<br>a3401_msms.txt<br>a7401_msms.txt<br>b1302_msms.txt<br>b4002_msms.txt<br>b5802_msms.txt<br>c0102_msms.txt<br>c0501_msms.txt<br>c1403_msms.txt<br>g0101_msms.txt<br>g0103_msms.txt<br>g0104_msms.txt	PXD034056
S9 (A)	b07_rna_peaks.csv<br>b07_rna_maxquant.txt<br>b07_rna_mascot1.csv<br>b07_rna_mascot2.csv<br>b07_rna_mascot3.csv<br>b07_rna_mascot4.csv<br>b07_rna_mascot5.csv<br>b07_rna_mascot6.csv	PXD034056
S9 (B)	b07 gencode_peaks.csv<br>b07 gencode maxquant.txt<br>b07 gencode mascot1.csv<br>b07 gencode mascot2.csv<br>b07 gencode mascot3.csv<br>b07 gencode mascot4.csv<br>b07 gencode mascot5.csv<br>b07_rna_macot6.csv	PXD034056
S9 (C)	tryptic_rna_peaks.csv<br>tryptic_rna_maxquant,txt<br>tryptic_rna_mascot1.csv<br>tryptic_rna_mascot2.csv<br>tryptic_rna_mascot3.csv<br>tryptic_rna_mascot4.csv<br>tryptic_rna_mascot5.csv<br>tryptic_rna_mascot6.csv<br>tryptic_rna_mascot7.csv<br>tryptic_rna_mascot8.csv<br>tryptic_rna_mascot9.csv<br>tryptic_rna_mascot10.csv<br>tryptic_rna_mascot11.csv<br>tryptic_rna_mascot12.csv	PXD034056
S9 (D)	tryptic_gencode_peaks.csv<br>tryptic_gencode_maxquant,txt<br>tryptic gencode mascot1.csv<br>tryptic gencode mascot2.csv<br>tryptic gencode mascot3.csv<br>tryptic gencode mascot4.csv<br>tryptic gencode mascot5.csv<br>tryptic gencode mascot6.csv<br>tryptic gencode mascot7.csv<br>tryptic gencode mascot8.csv<br>tryptic gencode mascot9.csv<br>tryptic gencode mascot10.csv<br>tryptic gencode mascot11.csv	PXD034056

	tryptic gencode mascot12.csv	
S10(A)	b07_rna_peaks.csv<br>b07_rna_maxquant.txt<br>b07_rna_mascot1.csv<br>b07_rna_mascot2.csv<br>b07_rna_mascot3.csv<br>b07_rna_mascot4.csv<br>b07_rna_mascot5.csv<br>b07_rna_mascot6.csv	PXD034056
S10(B)	tryptic_rna_peaks.csv<br>tryptic_rna_maxquant,txt<br>tryptic_rna_mascot1.csv<br>tryptic_rna_mascot2.csv<br>tryptic_rna_mascot3.csv<br>tryptic_rna_mascot4.csv<br>tryptic_rna_mascot5.csv<br>tryptic_rna_mascot6.csv<br>tryptic_rna_mascot7.csv<br>tryptic_rna_mascot8.csv<br>tryptic_rna_mascot9.csv<br>tryptic_rna_mascot10.csv<br>tryptic_rna_mascot11.csv<br>tryptic_rna_mascot12.csv	PXD034056
S11	PaesBorrows_DB_search_psm.csv<br>20160513_TIL1_R1/peaks_search.csv<br>20160513_TIL2_R1/peaks_search.csv<br>20160513_TIL3_R1/peaks_search.csv<br>20160513_TIL4_R1/peaks_search.csv<br>20160517_DC1W6_R1/peaks_search.csv<br>20160517_DC3W6_R1/peaks_search.csv<br>20160517_DC5W6_R1/peaks_search.csv<br>20160517_GD149-2_R1/peaks_search.csv<br>20160517_GD149-4_R1/peaks_search.csv<br>20160617_PD42_w6_S1_R1/peaks_search.csv<br>20160730_QEh1_LC1_HuPa_SA_Apher-1_Amino_1/peaks_search.csv<br>20160730_QEh1_LC1_HuPa_SA_Apher-6_Amino_1/peaks_search.csv<br>20160823_QEh1_LC2_HuPa_SA_HLApI_CM647_2_MG_1/peaks_search.csv<br>20160823_QEh1_LC2_HuPa_SA_HLApI_CM647_3_MG_1/peaks_search.csv<br>20160823_QEh1_LC2_HuPa_SA_HLApI_RA957_1_MG_1/peaks_search.csv<br>20160823_QEh1_LC2_HuPa_SA_HLApI_RA957_2_MG_1/peaks_search.csv<br>20160902_QEh1_LC2_CHC_SA_HLApI_MD155_1/peaks_search.csv<br>20160902_QEh1_LC2_CHC_SA_HLApI_pooledTIL3_1/peaks_search.csv<br>TUM_HLA_49_01_01-3xHCD-1h-R1-unspecific/msms.txt<br>TUM_HLA_82_01_01-3xHCD-1h-R1-unspecific/msms.txt<br>TUM_HLA_77_01_01-3xHCD-1h-R4-unspecific/msms.txt<br>TUM_HLA_10_01_01-3xHCD-1h-R4-unspecific/msms.txt<br>TUM_HLA_11_01_01-3xHCD-1h-R4-unspecific/msms.txt<br>TUM_HLA_12_01_01-3xHCD-1h-R4-unspecific/msms.txt<br>TUM_HLA_1_01_01-3xHCD-1h-R4-unspecific/msms.txt<br>TUM_HLA_2_01_01-3xHCD-1h-R4-unspecific/msms.txt<br>TUM_HLA_3_01_01-3xHCD-1h-R4-unspecific/msms.txt<br>TUM_HLA_4_01_01-3xHCD-1h-R4-unspecific/msms.txt<br>TUM_HLA_5_01_01-3xHCD-1h-R4-unspecific/msms.txt<br>TUM_HLA_6_01_01-3xHCD-1h-R4-unspecific/msms.txt<br>TUM_HLA_7_01_01-3xHCD-1h-R4-unspecific/msms.txt	PXD034056,<br>PXD005231

	TUM_HLA_8_01_01-3xHCD-1h-R4-unspecific/msms.txt<br>TUM_HLA_9_01_01-3xHCD-1h-R4-unspecific/msms.txt<br>TUM_HLA_22_01_01-3xHCD-1h-R4-unspecific/msms.txt<br>TUM_HLA_23_01_01-3xHCD-1h-R4-unspecific/msms.txt<br>TUM_HLA_24_01_01-3xHCD-1h-R4-unspecific/msms.txt<br>TUM_HLA_13_01_01-3xHCD-1h-R4-unspecific/msms.txt<br>TUM_HLA_14_01_01-3xHCD-1h-R4-unspecific/msms.txt<br>TUM_HLA_15_01_01-3xHCD-1h-R4-unspecific/msms.txt<br>TUM_HLA_16_01_01-3xHCD-1h-R4-unspecific/msms.txt<br>TUM_HLA_17_01_01-3xHCD-1h-R4-unspecific/msms.txt<br>TUM_HLA_18_01_01-3xHCD-1h-R4-unspecific/msms.txt<br>TUM_HLA_19_01_01-3xHCD-1h-R4-unspecific/msms.txt<br>TUM_HLA_20_01_01-3xHCD-1h-R4-unspecific/msms.txt<br>TUM_HLA_21_01_01-3xHCD-1h-R4-unspecific/msms.txt<br>TUM_HLA_34_01_01-3xHCD-1h-R4-unspecific/msms.txt<br>TUM_HLA_35_01_01-3xHCD-1h-R4-unspecific/msms.txt<br>TUM_HLA_36_01_01-3xHCD-1h-R4-unspecific/msms.txt<br>TUM_HLA_25_01_01-3xHCD-1h-R4-unspecific/msms.txt<br>TUM_HLA_26_01_01-3xHCD-1h-R4-unspecific/msms.txt<br>TUM_HLA_27_01_01-3xHCD-1h-R4-unspecific/msms.txt<br>TUM_HLA_28_01_01-3xHCD-1h-R4-unspecific/msms.txt<br>TUM_HLA_29_01_01-3xHCD-1h-R4-unspecific/msms.txt<br>TUM_HLA_30_01_01-3xHCD-1h-R4-unspecific/msms.txt<br>TUM_HLA_31_01_01-3xHCD-1h-R4-unspecific/msms.txt<br>TUM_HLA_32_01_01-3xHCD-1h-R4-unspecific/msms.txt<br>TUM_HLA_33_01_01-3xHCD-1h-R4-unspecific/msms.txt<br>TUM_HLA_46_01_01-3xHCD-1h-R4-unspecific/msms.txt<br>TUM_HLA_47_01_01-3xHCD-1h-R4-unspecific/msms.txt<br>TUM_HLA_48_01_01-3xHCD-1h-R4-unspecific/msms.txt<br>TUM_HLA_37_01_01-3xHCD-1h-R4-unspecific/msms.txt<br>TUM_HLA_38_01_01-3xHCD-1h-R4-unspecific/msms.txt<br>TUM_HLA_39_01_01-3xHCD-1h-R4-unspecific/msms.txt<br>TUM_HLA_40_01_01-3xHCD-1h-R4-unspecific/msms.txt<br>TUM_HLA_41_01_01-3xHCD-1h-R4-unspecific/msms.txt<br>TUM_HLA_42_01_01-3xHCD-1h-R4-unspecific/msms.txt<br>TUM_HLA_43_01_01-3xHCD-1h-R4-unspecific/msms.txt<br>TUM_HLA_44_01_01-3xHCD-1h-R4-unspecific/msms.txt<br>TUM_HLA_45_01_01-3xHCD-1h-R4-unspecific/msms.txt<br>TUM_HLA_58_01_01-3xHCD-1h-R4-unspecific/msms.txt<br>TUM_HLA_59_01_01-3xHCD-1h-R4-unspecific/msms.txt<br>TUM_HLA_60_01_01-3xHCD-1h-R4-unspecific/msms.txt<br>TUM_HLA_50_01_01-3xHCD-1h-R4-unspecific/msms.txt<br>TUM_HLA_51_01_01-3xHCD-1h-R4-unspecific/msms.txt<br>TUM_HLA_52_01_01-3xHCD-1h-R4-unspecific/msms.txt<br>TUM_HLA_53_01_01-3xHCD-1h-R4-unspecific/msms.txt<br>TUM_HLA_54_01_01-3xHCD-1h-R4-unspecific/msms.txt<br>TUM_HLA_55_01_01-3xHCD-1h-R4-unspecific/msms.txt<br>TUM_HLA_56_01_01-3xHCD-1h-R4-unspecific/msms.txt<br>TUM_HLA_57_01_01-3xHCD-1h-R4-unspecific/msms.txt<br>TUM_HLA_70_01_01-3xHCD-1h-R4-unspecific/msms.txt<br>TUM_HLA_71_01_01-3xHCD-1h-R4-unspecific/msms.txt<br>TUM_HLA_72_01_01-3xHCD-1h-R4-unspecific/msms.txt<br>TUM_HLA_61_01_01-3xHCD-1h-R4-unspecific/msms.txt<br>TUM_HLA_62_01_01-3xHCD-1h-R4-unspecific/msms.txt<br>TUM_HLA_63_01_01-3xHCD-1h-R4-unspecific/msms.txt<br>TUM_HLA_64_01_01-3xHCD-1h-R4-unspecific/msms.txt	

	TUM_HLA_65_01_01-3xHCD-1h-R4-unspecific/msms.txt<br>TUM_HLA_66_01_01-3xHCD-1h-R4-unspecific/msms.txt<br>TUM_HLA_67_01_01-3xHCD-1h-R4-unspecific/msms.txt<br>TUM_HLA_68_01_01-3xHCD-1h-R4-unspecific/msms.txt<br>TUM_HLA_69_01_01-3xHCD-1h-R4-unspecific/msms.txt<br>TUM_HLA_83_01_01-3xHCD-1h-R4-unspecific/msms.txt<br>TUM_HLA_84_01_01-3xHCD-1h-R4-unspecific/msms.txt<br>TUM_HLA_73_01_01-3xHCD-1h-R4-unspecific/msms.txt<br>TUM_HLA_74_01_01-3xHCD-1h-R4-unspecific/msms.txt<br>TUM_HLA_75_01_01-3xHCD-1h-R4-unspecific/msms.txt<br>TUM_HLA_76_01_01-3xHCD-1h-R4-unspecific/msms.txt<br>TUM_HLA_78_01_01-3xHCD-1h-R4-unspecific/msms.txt<br>TUM_HLA_79_01_01-3xHCD-1h-R4-unspecific/msms.txt<br>TUM_HLA_80_01_01-3xHCD-1h-R4-unspecific/msms.txt<br>TUM_HLA_81_01_01-3xHCD-1h-R4-unspecific/msms.txt<br>TUM_HLA_94_01_01-3xHCD-1h-R4-unspecific/msms.txt<br>TUM_HLA_95_01_01-3xHCD-1h-R4-unspecific/msms.txt<br>TUM_HLA_96_01_01-3xHCD-1h-R4-unspecific/msms.txt<br>TUM_HLA_85_01_01-3xHCD-1h-R4-unspecific/msms.txt<br>TUM_HLA_86_01_01-3xHCD-1h-R4-unspecific/msms.txt	
S12	See Fig. S11	PXD034056,<br>PXD005231
S13	b07_single_rep_rna_msms.txt<br>b07_single_rep_gencode_msms.txt<br>a02_single_rep_rna_msms.txt<br>a02_single_rep_rna_msms.txt	PXD034056
S14	b07_single_rep_rna_msms.txt<br>b07_single_rep_gencode_msms.txt<br>a02_single_rep_rna_msms.txt<br>a02_single_rep_rna_msms.txt	PXD034056

Table S5. Search result files. Details of the search result files from which each of the figures has been generated.

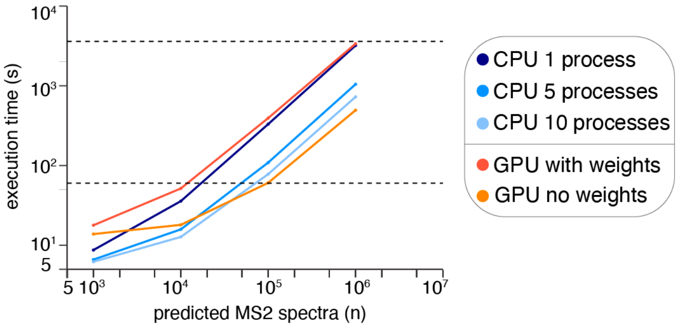

Figure S1. Timing of Prosit MS2 spectral prediction via inSPIRE (CPU) and the original Prosit library (GPU only). Execution time for Prosit prediction of 1,000 to 1,000,000 MS2 spectra for different hardware and software combinations. Execution times are shown for two variants of the original Prosit code running on GPU, one of which computes m/z values for all possible fragment ions and one of which reports the fragment ion name only. Execution times are also shown for CPU predictions which are made using the inSPIRE "predictSpectra" pipeline. They do not compute m/z values of the fragment ions, *i.e.*, report fragment ion names only. The dashed lines represent one minute and one hour of run time.

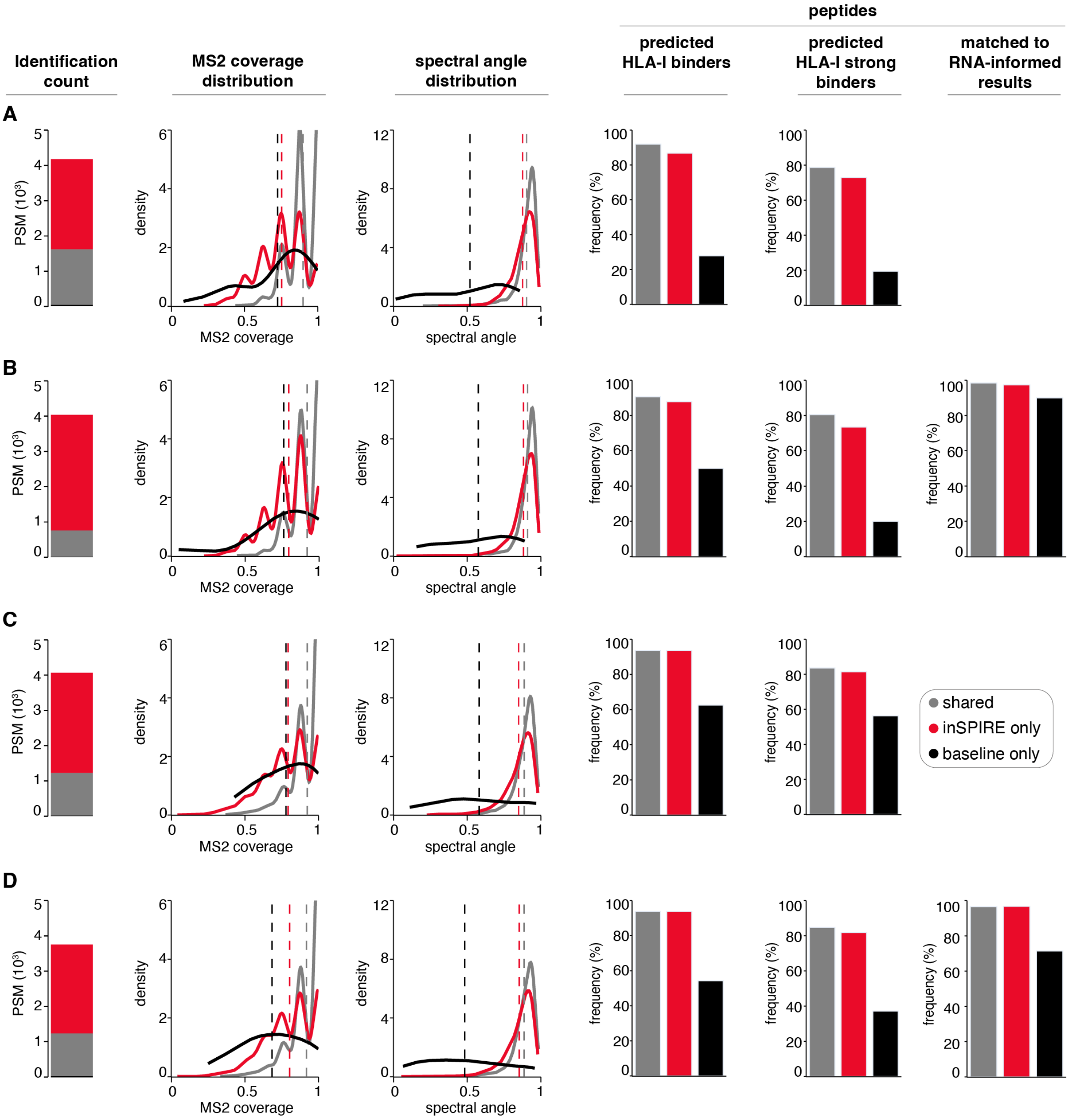

Figure S2. Incremental PSMs identified by inSPIRE and baseline rescoring pipelines. Comparison of the PSMs identified at 1% FDR by both inSPIRE and the baseline Percolator implementation, by inSPIRE only, and by the baseline implementation only, for MaxQuant search results of the HLA-I immunopeptidome of the K562-A*02:01 (**A-B**) and the K562-B*07:02 (**C-D**) cell lines. Results were obtained by searching an RNA-informed reference database (**A, C**) and the full Gencode reference database (**B, D**). Each panel shows the number of PSMs in each group, which is 743 - 1,581 PSMs shared, 2,556 - 3,283 PSMs for inSPIRE only, and 10 - 38 PSMs for the baseline only. The distribution of MS2 spectrum coverage and spectral angle, to provide insight into the reason for the identifications being made. As a validation two metrics are reported: (i-ii) the percentage of peptides predicted by NetMHCpan either to bind or to strongly bind the cognate HLA-I complexes; (iii) for search results that used the Gencode reference database, the percentage of identified peptides which were also identified when using the RNA-informed reference database. Dash line indicates mean value across all PSMs.

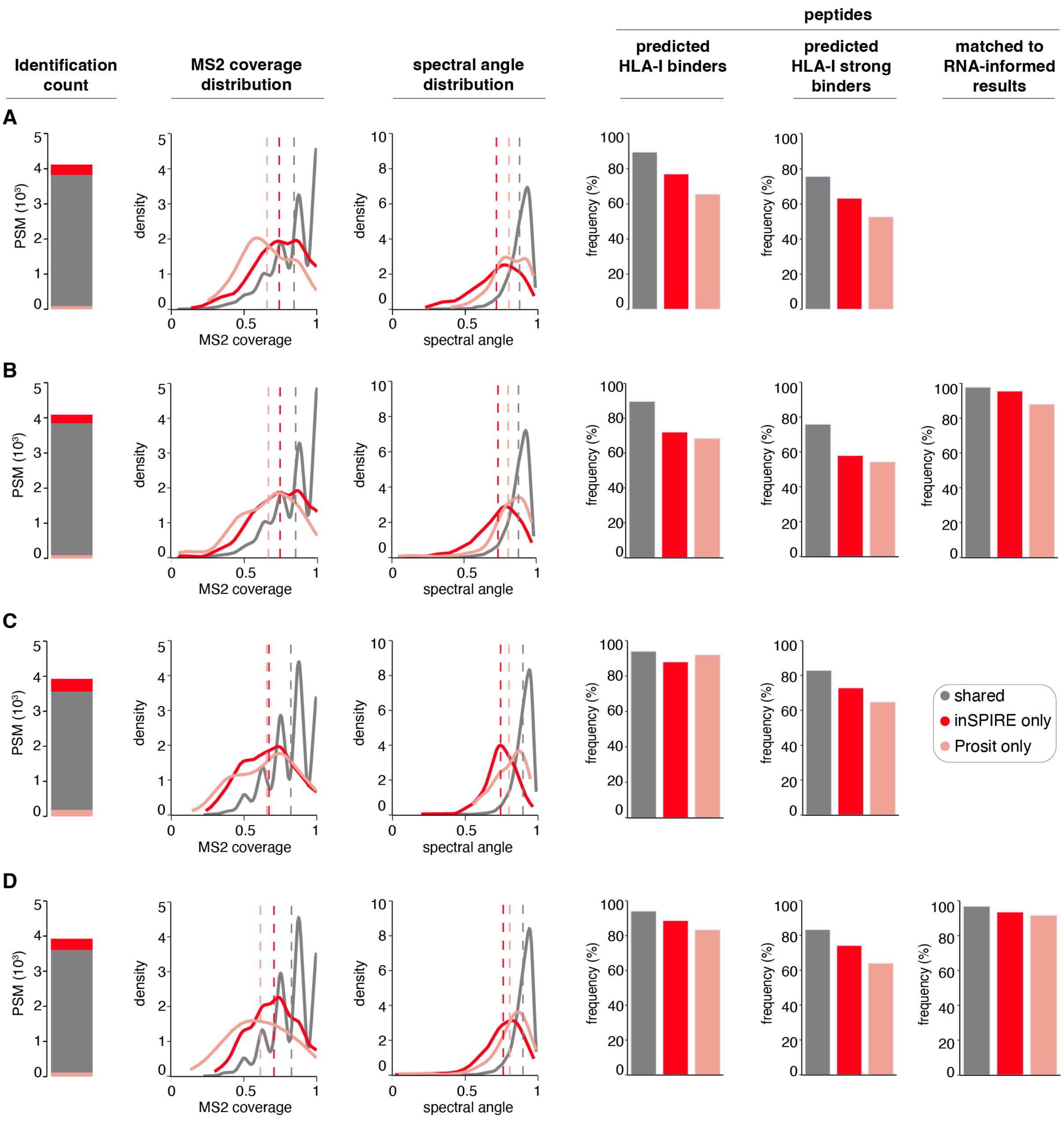

Figure S3. Incremental PSMs identified by inSPIRE and Prosit rescoring pipelines. Comparison of the PSMs identified at 1% FDR by both inSPIRE and the Prosit Rescoring pipeline, by inSPIRE only, and by the Prosit rescoring only, in the HLA-I immunopeptidome of the K562-A*02:01 (**A-B**) and the K562-B*07:02 (**C-D**) cell lines. Results were obtained by searching an RNA-informed reference database (**A, C**) and the full Gencode reference database (**B, D**). Each panel shows the number of PSMs in each group, which is: 3,372 - 3,873 PSMs shared, 264 - 366 PSMs for inSPIRE only, and 92 - 188 PSMs for the Prosit rescoring only. The distribution of MS2 spectrum coverage and spectral angle is also shown to provide insight into the reason for the identifications being made. As a validation two metrics are reported: (i-ii) the percentage of peptides predicted by NetMHCpan either to bind or to strongly bind the cognate HLA-I complexes; (iii) for search results that used the Gencode reference database, the percentage of identified peptides which were also identified when using the RNA-informed reference database. Dash line indicates mean value across all PSMs.

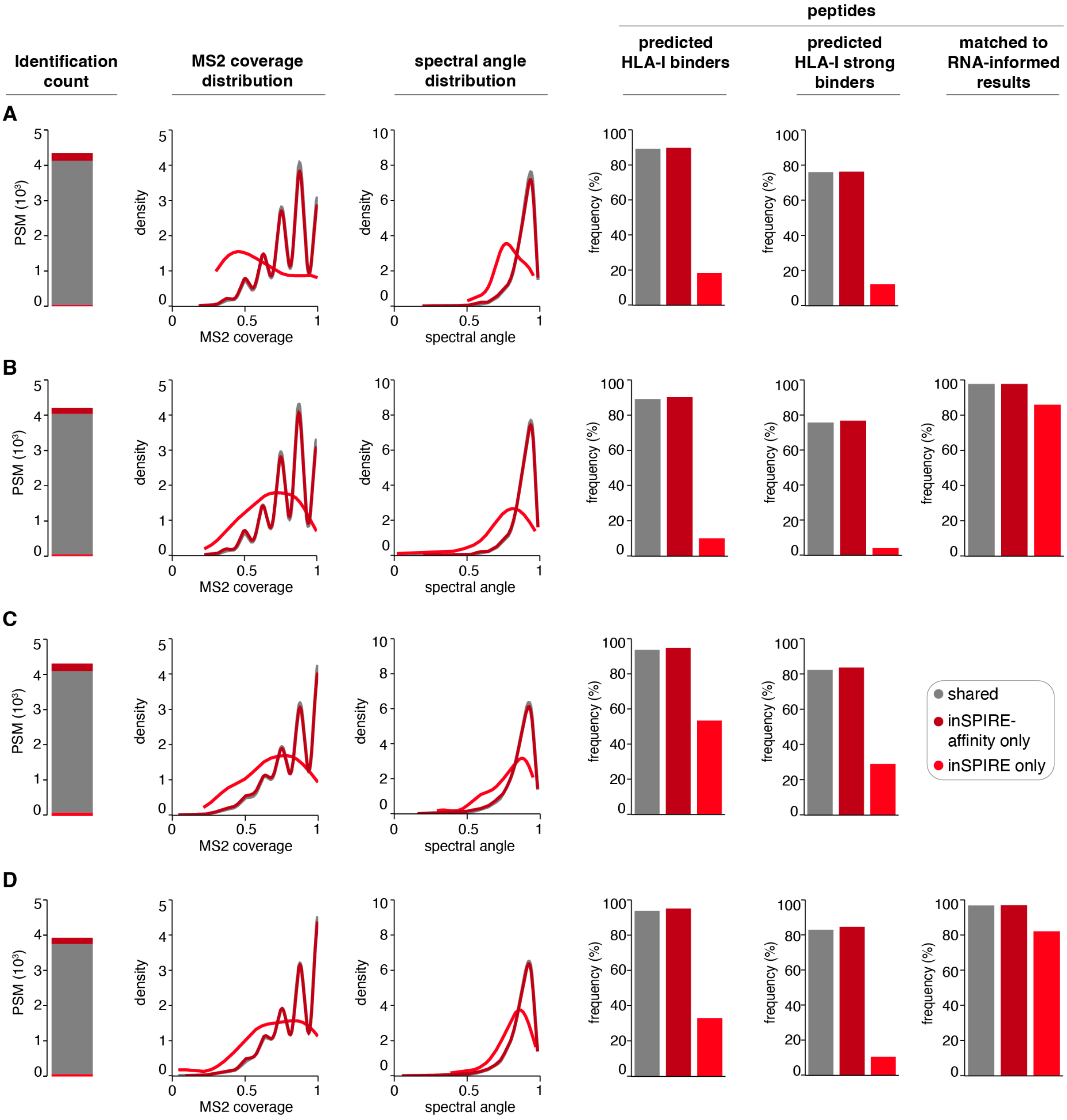

Figure S4. Incremental PSMs identified by inSPIRE-affinity and standard inSPIRE pipelines. Comparison of the PSMs identified at 1% FDR by both inSPIRE-affinity and standard inSPIRE, by inSPIRE-affinity only, and by standard inSPIRE only, for MaxQuant search results of the HLA-I immunopeptidome of the K562-A*02:01 (**A-B**) and the K562-B*07:02 (**C-D**) cell lines. Results were obtained by searching an RNA-informed reference database (**A, C**) and the full Gencode reference database (**B, D**). Each panel shows the number of PSMs in each group, which is: 3,734 - 4,137 PSMs shared, 168 - 214 PSMs for inSPIRE-affinity only, and 33 - 90 PSMs for the inSPIRE only. The distribution of MS2 spectrum coverage and spectral angle is also shown to provide insight into the reason for the identifications being made. As a validation two metrics are reported: (i-ii) the percentage of peptides predicted by NetMHCpan either to bind or to strongly bind the cognate HLA-I complexes; (iii) for search results that used the Gencode reference database, the percentage of identified peptides which were also identified when using the RNA-informed reference database. Dash line indicates mean value across all PSMs.

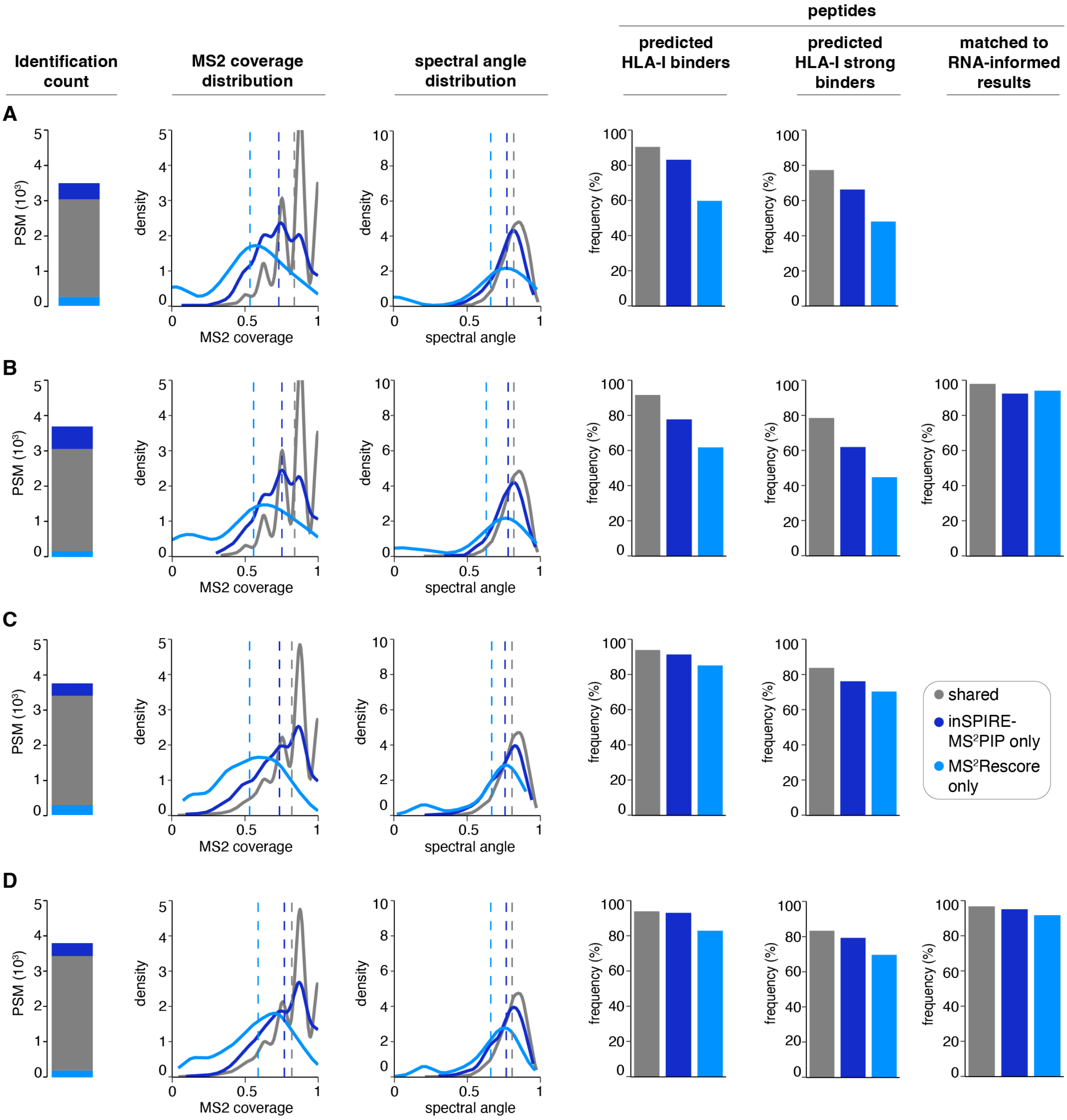

Figure S5. Incremental PSMs identified by inSPIRE-MS²PIP and MS²Rescore Pipelines. Comparison of the PSMs identified at 1% FDR by both inSPIRE-MS²PIP and MS²Rescore, by inSPIRE-MS²PIP only, and by MS²Rescore only, for MaxQuant search results of the HLA-I immunopeptidome of the K562-A*02:01 (**A-B**) and the K562-B*07:02 (**C-D**) cell lines. Results were obtained by searching an RNA-informed reference database (**A, C**) and the full Gencode reference database (**B, D**). Each panel shows the number of PSMs in each group, which is: 2,806 – 3,285 PSMs shared, 352 - 642 PSMs for inSPIRE-MS²PIP only, and 159 - 287 PSMs for the inSPIRE only. The distribution of MS2 spectrum coverage and spectral angle is also shown to provide insight into the reason for the identifications being made. As a validation two metrics are reported: (i-ii) the percentage of peptides predicted by NetMHCpan either to bind or to strongly bind the cognate HLA-I complexes; (iii) for search results that used the Gencode reference database, the percentage of identified peptides which were also identified when using the RNA-informed reference database. Dash line indicates mean value across all PSMs.

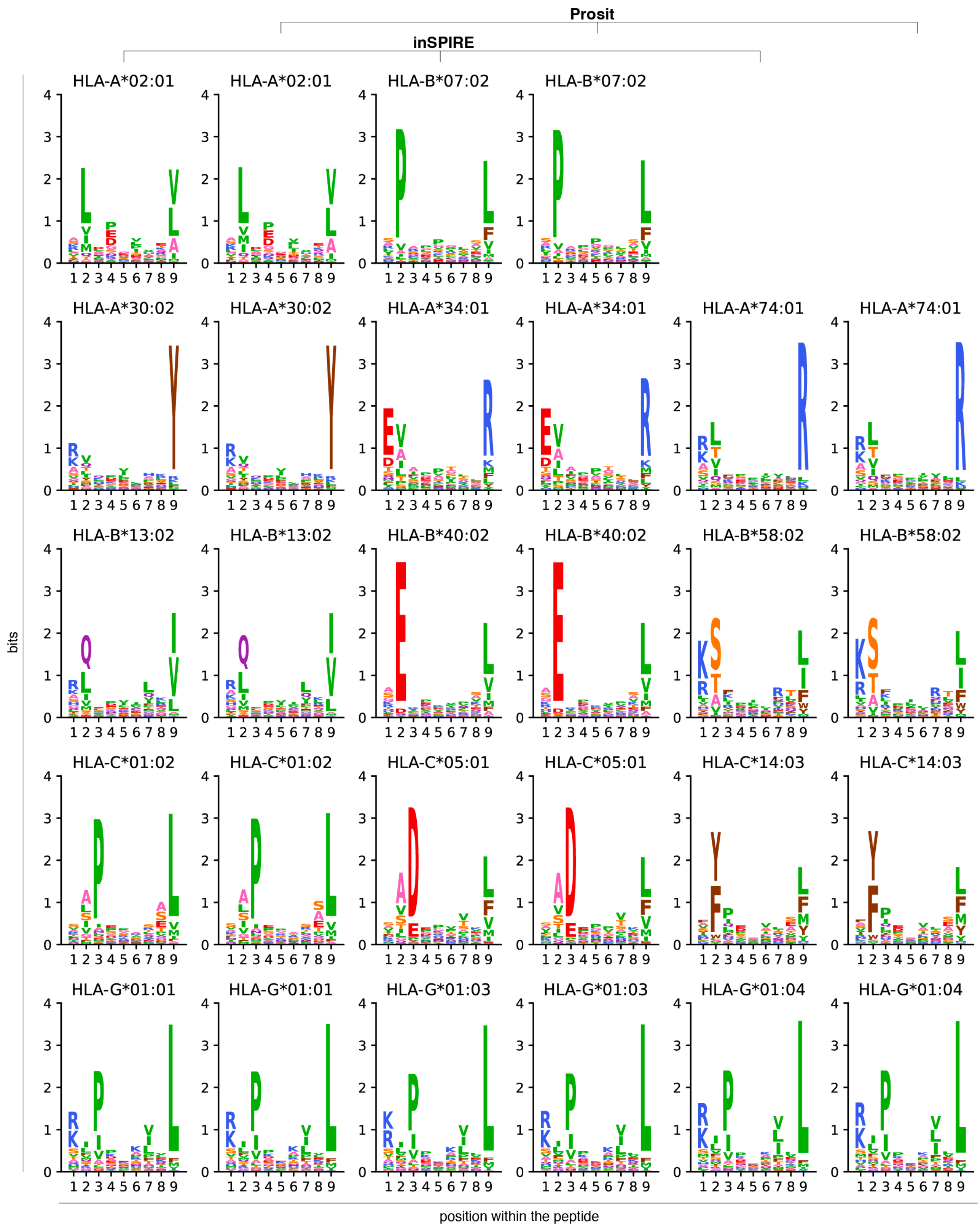

Figure S6. Sequence logo plots for 9 residue-long peptides Identified by inSPIRE and Prosit rescoring for all HLA-I Immunopeptidomes analyzed. Sequence logo plots are provided for peptides identified by inSPIRE and Prosit Rescoring at 1% FDR for all HLA-I immunopeptidomes analyzed. For consistency, all identifications are taken from rescoring of MaxQuant search results using the Gencode reference database. This data relates to the K562-B*07:02 and K562-A*02:01 cell lines used in **Fig. 2** and all HLA-I mono-allelic cell lines reanalyzed from Sarkizova *et al.* (55).

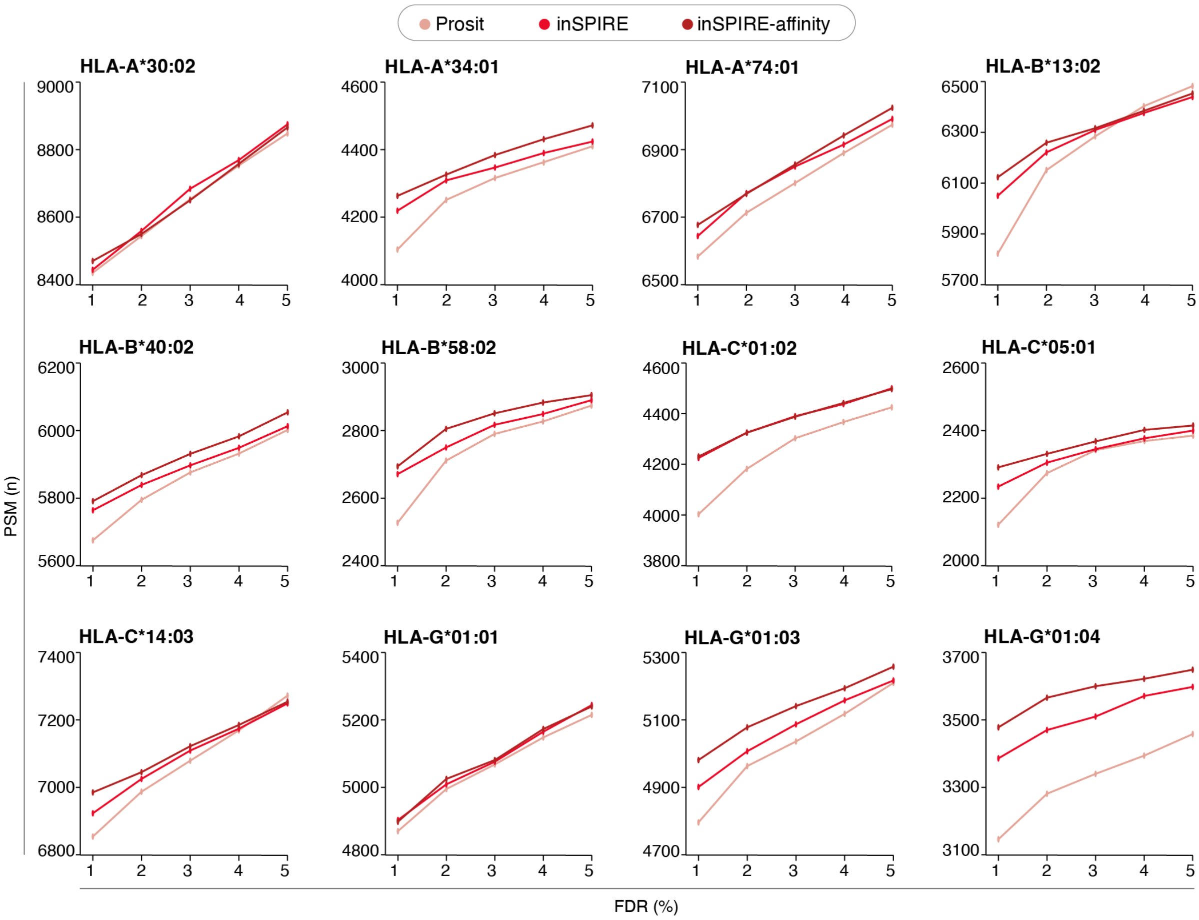

Figure S7. Number of PSMs identified by inSPIRE, inSPIRE-affinity and Prosit Rescoring across a variety of HLA-I monoallelic cell lines. Number of PSMs identified by Prosit rescoring pipeline, inSPIRE, and inSPIRE-affinity on MaxQuant search results of the HLA-I monoallelic immunopeptidomes HLA-A, HLA-B, HLA-C, HLA-G cell lines (n = 3 for each allele group) with an FDR range of 1-5%. This figure provides evidence of the inSPIRE software's performance across diverse HLA-I immunopeptidome datasets.

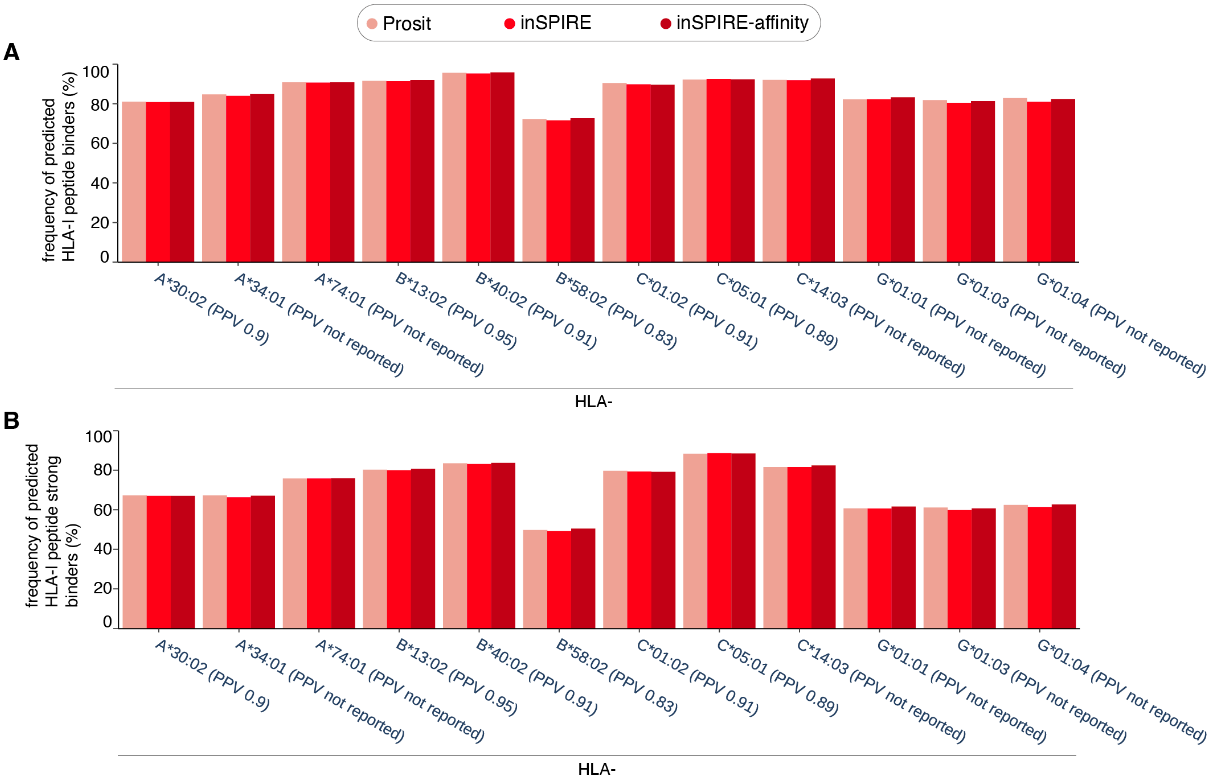

Figure S8. Percentage of peptides predicted to bind the cognate HLA-I complex, and identified by inSPIRE, inSPIRE-affinity and Prosit Rescoring in a variety of HLA-I monoallelic immunopeptidomes. (**A**,**B**) The percentage of peptides identified at 1% FDR by Prosit Rescoring, inSPIRE, and inSPIRE-affinity, which were predicted either to bind (**A**) or to strongly bind (**B**) the cognate HLA-I allele by NetMHCpan. For each HLA-I allele where the data was available, we provide the positive predicted value (PPV) reported by Reynisson *et al.* (37) to indicate the expected quality of the NetMHCpan predictions. This measure of PPV is defined as the number of positive binding peptides correctly predicted divided by 0.95 times the number of ligands predicted.

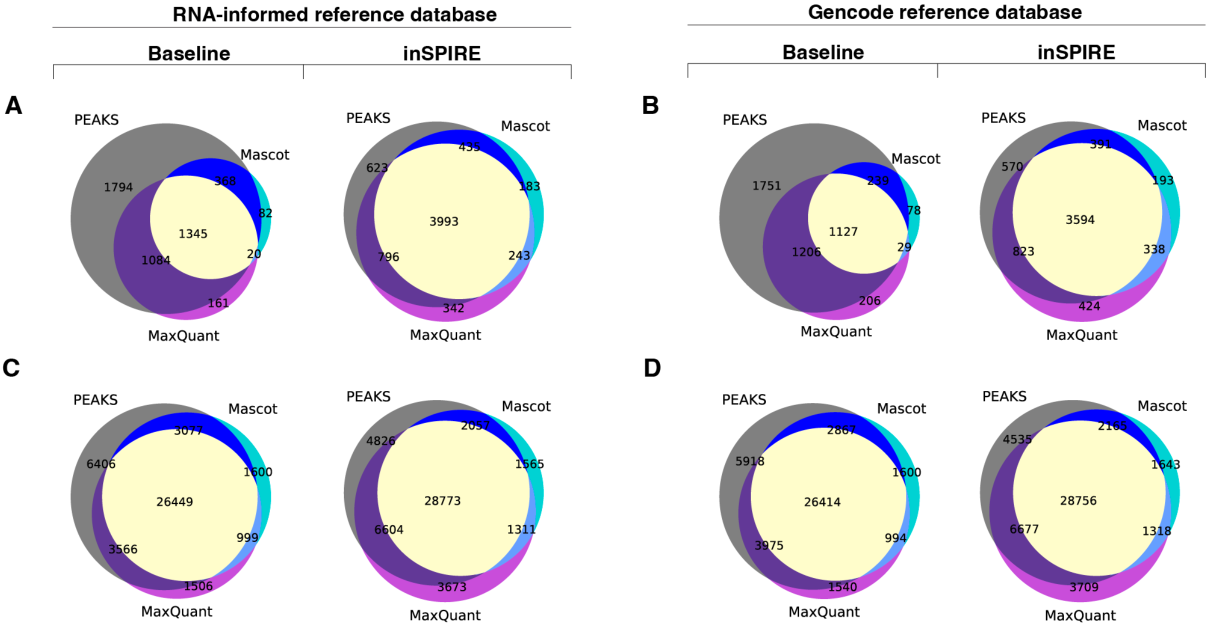

Figure S9. Overlap of peptides identified by Mascot, MaxQuant and PEAKS search engines with or without rescoring with inSPIRE. The Venn Diagrams illustrate the overlap in unique peptides identified using different search engines with and without rescoring by inSPIRE. Results are shown for the HLA-I immunopeptidome (**A-B**) and the tryptic proteome digestions (**C-D**) datasets. We used either RNA-informed reference database in (**A, C**) or the Gencode reference database in (**B,D**) in the analysis.

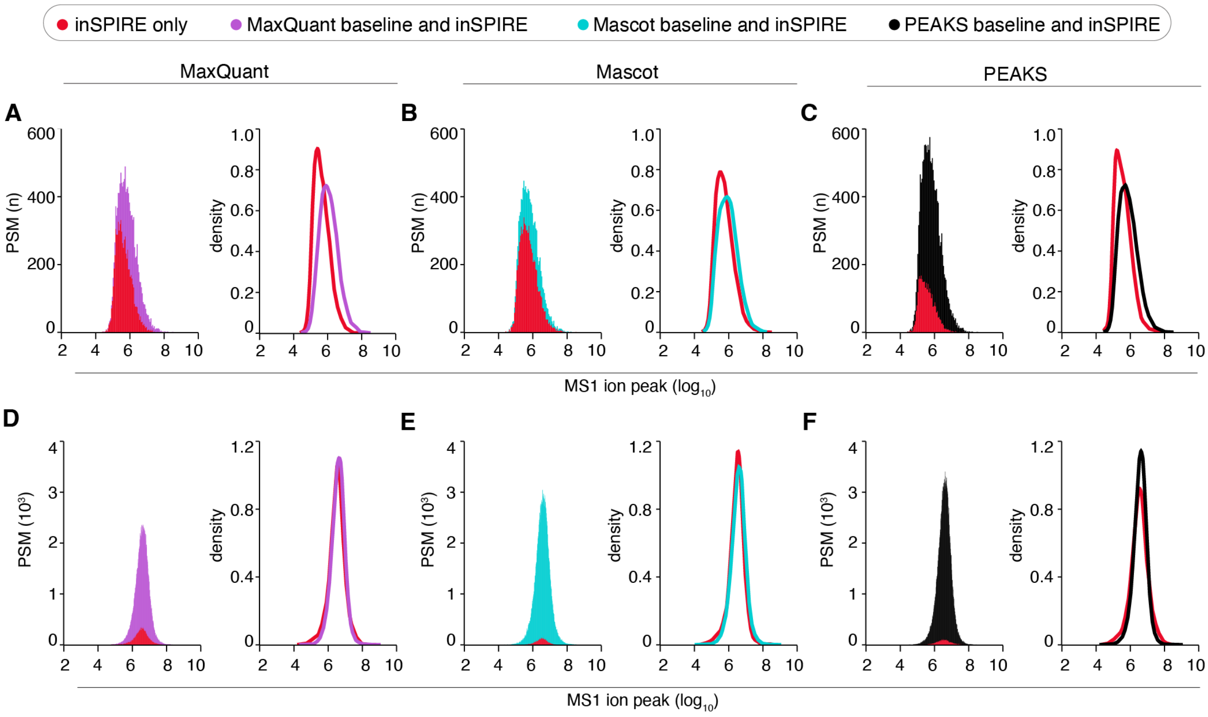

Figure S10. Distribution of MS1 ion peak intensity for PSMs identified by original search engine and inSPIRE, compared to PSMs identified by inSPIRE only. The distribution of MS1 ion peak intensity of the PSMs identified by both the baseline identification method and inSPIRE at 1% FDR, compared to those identified by inSPIRE only at 1% FDR. The results are shown for both HLA-I immunopeptidomes (**A-C**) and tryptic proteome digestions (**D-F**), using MaxQuant (**A, D**), Mascot (**B, D**), and PEAKS DB (**C, F**).

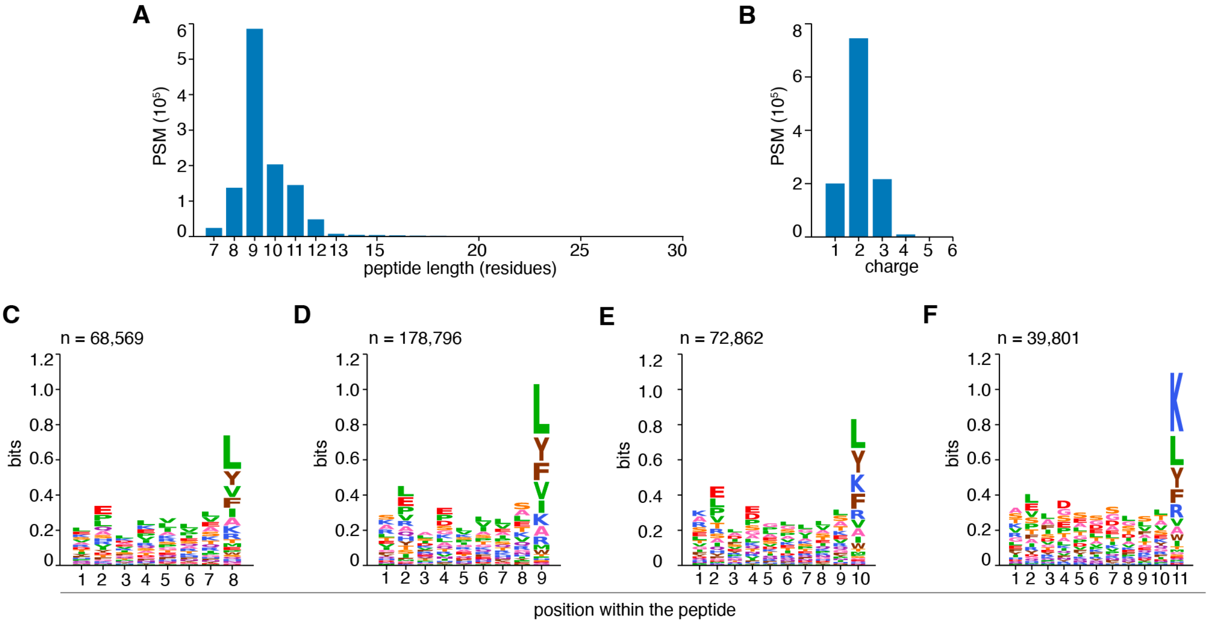

Figure S11. Distribution of sequence length, charge state and sequence motifs in the Prosit-*delta* training dataset. (**A-B**) The distribution of peptide length (**A**) and charge state (**B**) for PSMs used in the Prosit-*delta* training dataset are provided. (**C-F**) Sequence logo plots for the unique peptides of length 8 (**C**), 9 (**D**), 10 (**E**), and 11 (**F**) residues in the training dataset. It illustrates that the training dataset is not biased towards a specific sequence motif.

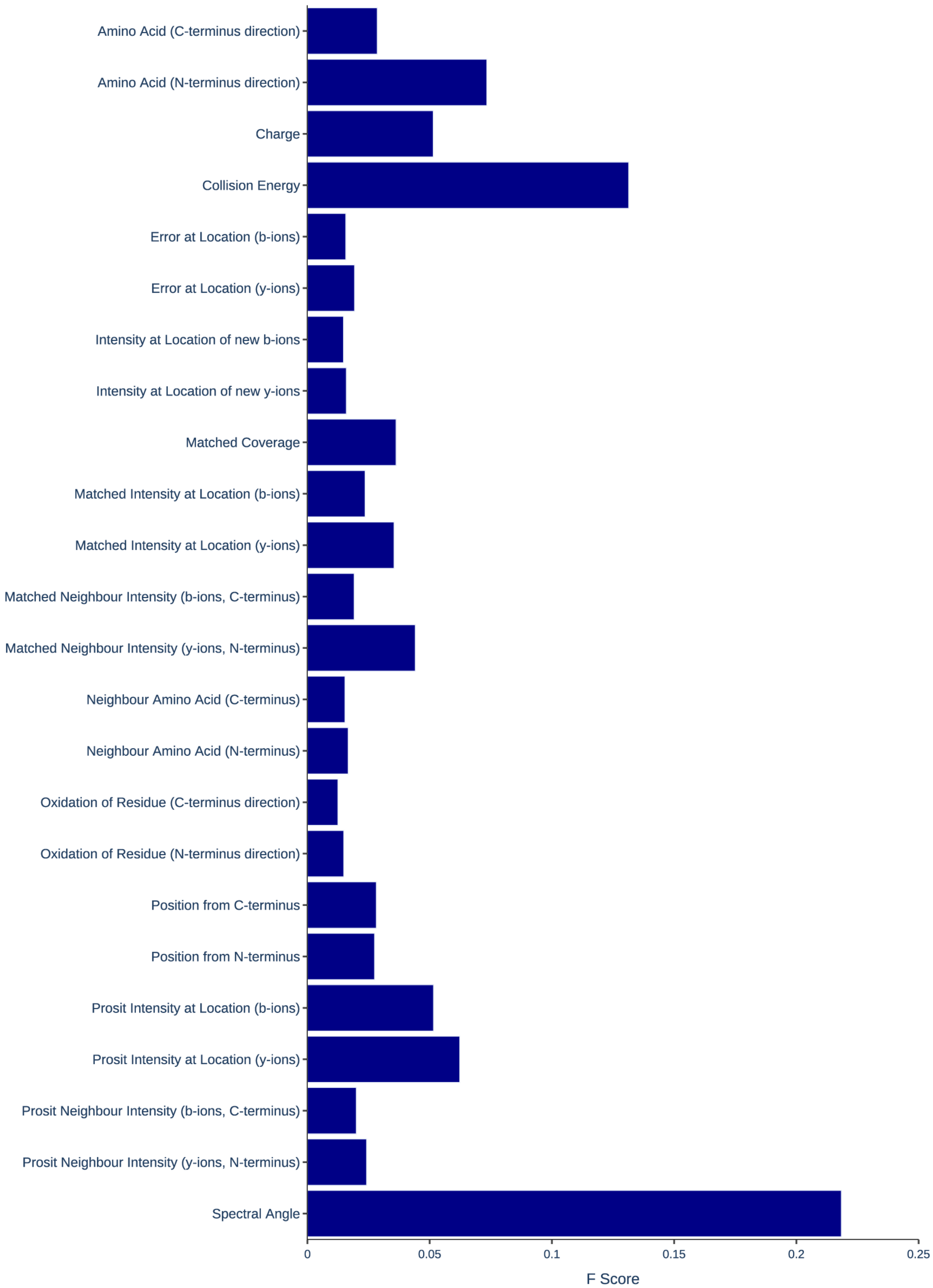

Figure S12. Feature importance values for the Prosit-*delta* regressor. Plotted is the F-score as reported by xgboost for all feature used to predict the Prosit-*delta* of a PSM. These features are further described in **Table S3**. This figure is provided to increase the understandability of the Prosit-*delta* model.

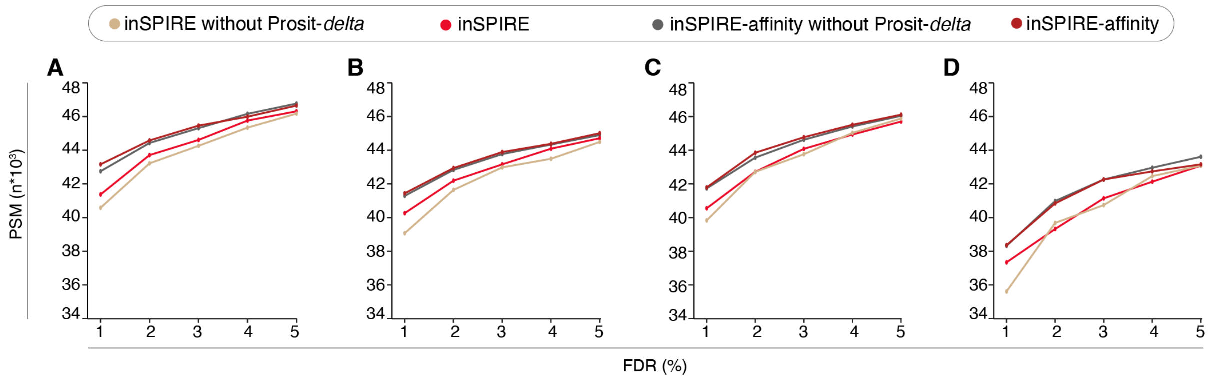

Figure S13. Impact of Prosit-*delta* feature on inSPIRE's PSM yield in HLA-I immunopeptidomics with an FDR range. Number of PSMs identified by inSPIRE with and without Prosit-*delta* features, and inSPIRE-affinity with and without Prosit-*delta* features on MaxQuant search results of the HLA-I immunopeptidomes of the K562-A*02:01 (**A,B**) and K562-B*07:02 (**C,D**) cell lines with an FDR range of 1-5%. This figure provides a comparison of the impact of different features comparing a search of RNA-informed (**A,C**) and the full Gencode (**B,D**) reference databases.

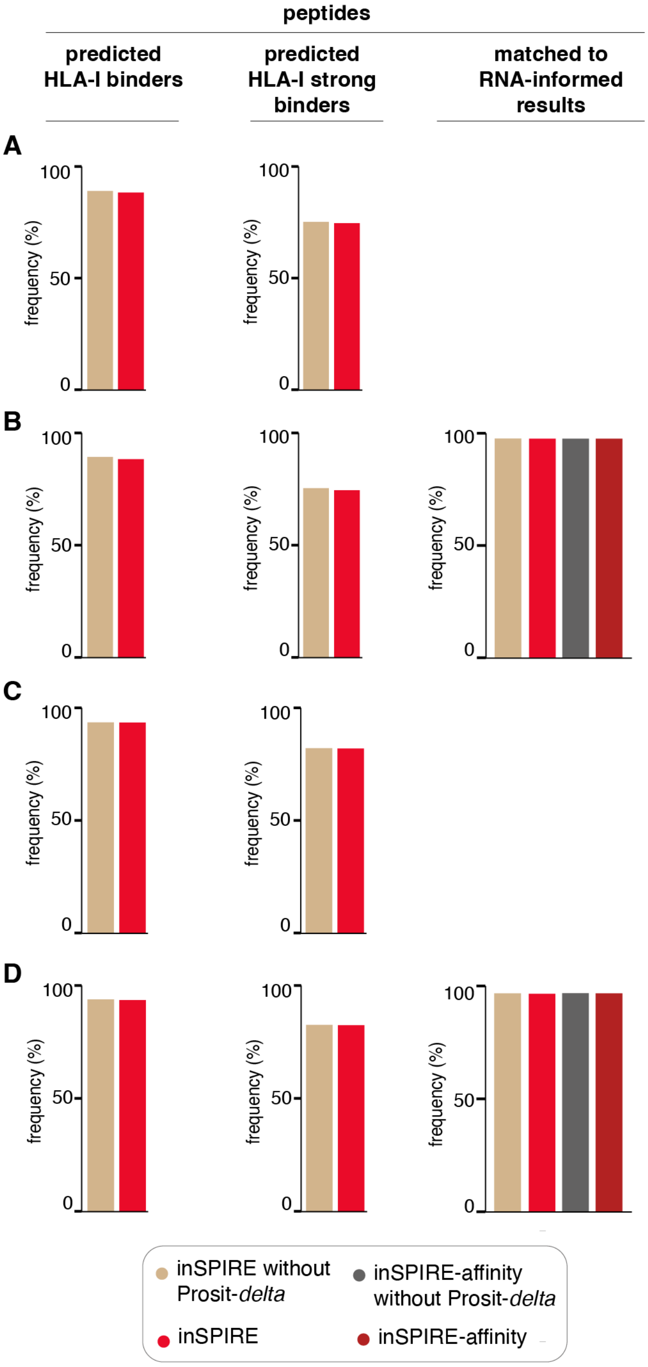

Figure S14. Validation of peptide identification by applying inSPIRE variants with or without Prosit-*delta* features. (**A-D**) The percentage of peptides predicted either to bind or to strongly bind the cognate HLA-I allele by NetMHCpan. Peptides were identified by applying either inSPIRE or inSPIRE without Prosit-*delta* features at 1% FDR for the HLA-I immunopeptidome of the K562-A*02:01 (**A,B**) and K562-B*07:02 (**C,D**) cell lines. Search results using either the RNA-informed (**A,C**) or full Gencode (**B,D**) reference databases are reported. For the peptides identified using the Gencode database, we also report the percentage of peptides which were also found when using the RNA-informed reference database for the analysis. For this latter analysis, we also include the results obtained by applying either inSPIRE-affinity or inSPIRE-affinity without Prosit-*delta* features.
